# Therapeutic outcomes of TP53-mutated diffuse large B cell lymphoma: A systematic review and meta-analysis

**DOI:** 10.1016/j.isci.2026.117049

**Published:** 2026-08-06

**Authors:** Xin Zhou, Yang Jiang, Ai Li, Dexiao Kong, Peipei Zhang, Ningning Shan

**Affiliations:** 1 Department of Hematology, The Second Qilu Hospital of Shandong University, 247 Beiyuan Road, Jinan, Shandong 250033, China; 2 Shandong Key Laboratory of Cancer Digital Medicine, Jinan, China

**Keywords:** diffuse large B cell lymphoma, TP53 mutations, chemotherapy, CAR-T

## Abstract

*TP53* mutations are associated with early progression and poor prognosis in diffuse large B cell lymphoma (DLBCL). This systematic review and meta-analysis evaluated remission outcomes of different therapies in *TP53*-mutated (TP53m) DLBCL. In total, 33,762 records were identified, and 31 studies comprising 1,164 patients with TP53m DLBCL were included. In the newly diagnosed (ND) setting, among patients treated with targeted therapy (TT) plus chemotherapy, the pooled complete remission (CR) rate was 60% (95% CI: 50%–69%; *n* = 552; *I*^2^ = 75.7%) and the pooled overall remission rate (ORR) was 80% (95% CI: 74%–86%; *n* = 202; *I*^2^ = 0%). In the relapsed/refractory (R/R) setting, among patients treated with chimeric antigen receptor T cell (CAR-T) therapy, the pooled CR rate was 39% (95% CI: 24%–54%; *n* = 139; *I*^2^ = 64.2%) and the pooled ORR was 77% (95% CI: 43%–99%; *n* = 131; *I*^2^ = 94.1%). TT plus chemotherapy and CAR-T-based therapy show encouraging activity in TP53-mutated DLBCL, but larger prospective studies are required to define their clinical roles.

## Highlights

• First meta-analysis of TP53-mutated DLBCL outcomes across ND and R/R settings
• TT plus chemotherapy and CAR-T therapy show potential benefit in TP53-mutated DLBCL
• Optimal treatment strategy for TP53-mutated DLBCL remains to be defined

## Graphical abstract

**Figure:**
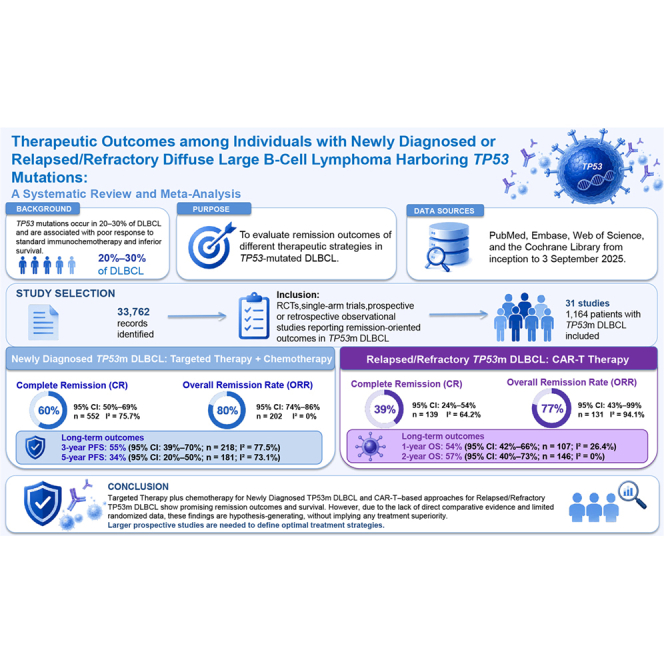

## Teaser

Health sciences; medicine; medical specialty; internal medicine; hematology

## Introduction

Diffuse large B cell lymphoma (DLBCL) is biologically heterogeneous, and molecular risk stratification has become increasingly important for identifying patients who are unlikely to benefit from standard immunochemotherapy.^1^^,^^2^ Among recurrent genetic abnormalities, *TP53* aberrations represent one of the most clinically relevant adverse molecular features. *TP53* encodes a central tumor-suppressor protein involved in the DNA-damage response, cell-cycle arrest, apoptosis, senescence, and the maintenance of genomic stability.^3^ Disruption of this pathway may promote genomic instability, treatment resistance, and aggressive disease behavior. Recent molecular classifications have illuminated the pivotal role of *TP53* pathway disruption, showing that *TP53* mutations and/or deletions are highly enriched in high-risk genetic categories, such as the A53 or *TP53*-mutated (TP53m) subtype.^4^^,^^5^ Large cohort studies and genomic analyses have consistently shown that *TP53* mutations are detected in approximately 20%–30% of cases with DLBCL. Meanwhile, *TP53* mutations are associated with inferior therapeutic responses, as well as shorter progression-free survival (PFS) and overall survival (OS).^6^^,^^7^^,^^8^ Recent evidence also suggests that the prognostic effect of *TP53* mutations may be context-dependent and influenced by coexisting molecular features, including the cell-of-origin, double-expression phenotype, copy-number alterations, and other recurrent mutations.^9^

The clinical management of patients with TP53m DLBCL remains particularly challenging. Standard rituximab + cyclophosphamide + hydroxydaunorubicin + oncovin + prednisone (R-CHOP) or R-CHOP-like immunochemotherapy frequently provides insufficient disease control in this population. In studies specifically enrolling patients with TP53m DLBCL, those receiving first-line standard immunochemotherapy demonstrate a median PFS of less than 12 months and a median OS ranging from approximately 1 to 2 years. In some high-risk subgroups, the 5-year OS was only as low as 15%–20%.^9^^,^^10^ These findings suggest that *TP53* mutation represents a prognostic marker and a clinically relevant indicator of early treatment failure and therapeutic resistance.

Despite this recognized high-risk biology, the optimal therapeutic strategy for TP53m DLBCL remains underexplored. Recently, improving therapeutic efficacy and survival among individuals with TP53m DLBCL has been increasingly explored in numerous clinical trials. Multiple novel therapeutic combinations are being incorporated into first-line trials targeting populations with TP53m DLBCL or other ultra-high-risk subtypes.^11^ These regimens include agents such as Bruton tyrosine kinase inhibitors (BTKi), BCL-2 inhibitors (BCL-2i), immunomodulatory drugs (IMiDs), chimeric antigen receptor T cell (CAR-T) therapy, and other emerging therapeutic approaches. These strategies seek to overcome the inadequate efficacy of conventional immunochemotherapy in the context of *TP53* abnormalities.^12^ Recent trials such as POLARIX and ZUMA-12, along with ongoing studies of frontline CAR-T and bispecific antibody combination strategies, have suggested that these emerging regimens might improve deep response rates and long-term disease control in biologically high-risk subgroups.^13^^,^^14^ However, current evidence is fragmented across randomized trials, single-arm studies, and retrospective cohorts, and data on TP53m cases are largely confined to subgroup analyses. Moreover, treatment outcomes may differ substantially between newly diagnosed (ND) and relapsed/refractory (R/R) disease. Consequently, it is difficult to elucidate the clinical value of different therapeutic approaches based on individual studies alone. Therefore, this systematic review and meta-analysis (SRMA) was conducted to comprehensively evaluate current induction strategies and their therapeutic efficacy in TP53m DLBCL.

## Results

### Characteristics of included studies and patients

**Figure 1:**
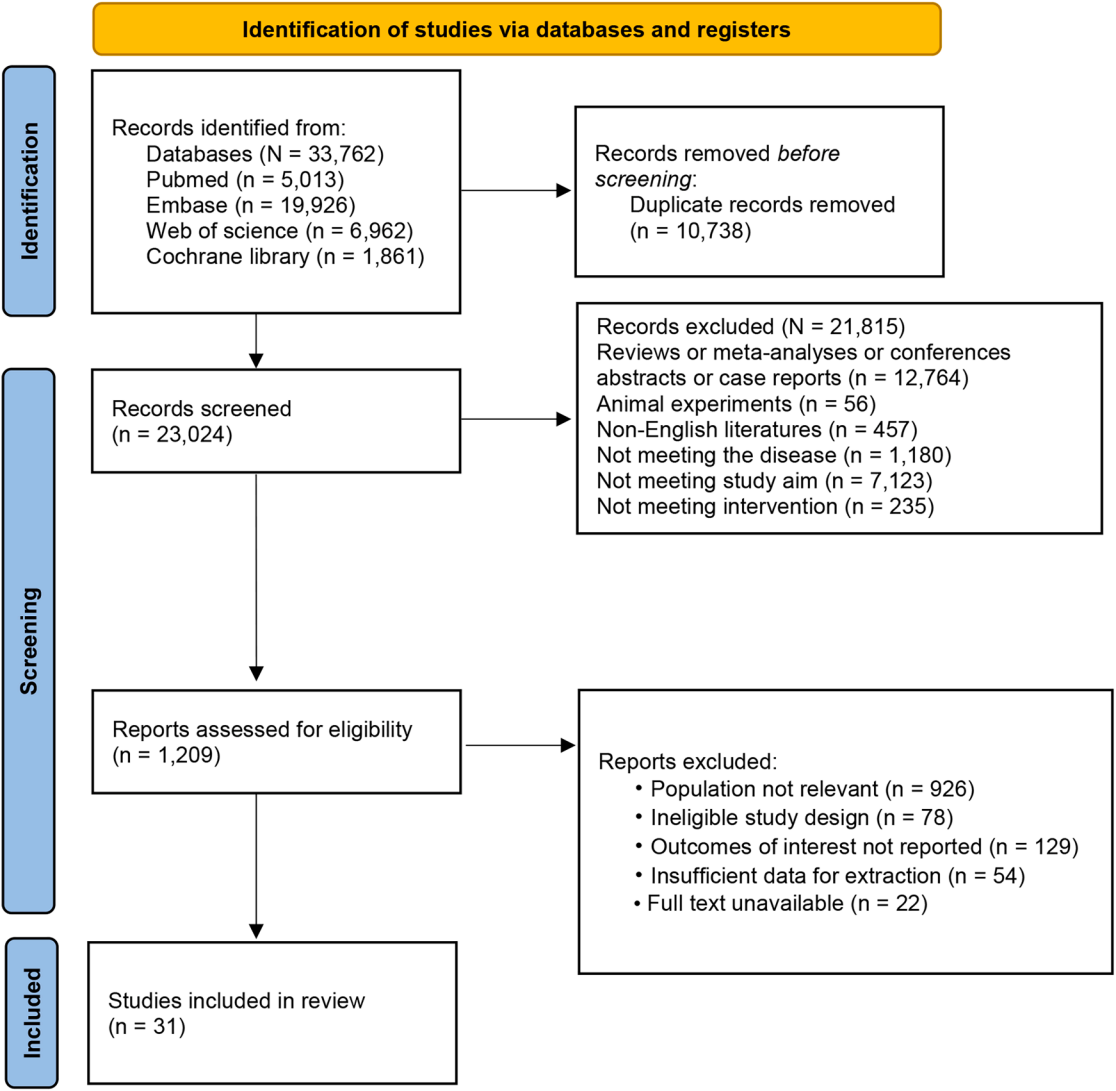
Flow diagram of study selection

The process of study selection is demonstrated in Figure 1. In total, 33,762 records were identified through the aforementioned four databases. After removal of 10,738 duplicates and exclusion of 21,815 records based upon title or abstract, 1,209 articles remained for eligibility assessment through complete-text review or abstract review. Ultimately, 31 studies were included in the SRMA,^7^^,^^8^^,^^10^^,^^11^^,^^15^^,^^16^^,^^17^^,^^18^^,^^19^^,^^20^^,^^21^^,^^22^^,^^23^^,^^24^^,^^25^^,^^26^^,^^27^^,^^28^^,^^29^^,^^30^^,^^31^^,^^32^^,^^33^^,^^34^^,^^35^^,^^36^^,^^37^^,^^38^^,^^39^^,^^40^^,^^41^ comprising 1,164 patients with TP53m DLBCL who met our prespecified inclusion criteria. Among these studies, 3 were randomized controlled trials (RCTs), 3 were single-arm clinical trials, 23 were retrospective observational studies, and 2 were prospective observational studies. The therapeutic methods evaluated for TP53m DLBCL included targeted therapy (TT), Chemo, TT + Chemo, allogeneic hematopoietic stem-cell transplantation (Allo-HSCT), CAR-T, and bispecific antibody therapy. Detailed characteristics of included studies and patients are demonstrated in Table 1. The treatment outcomes reported in the included studies are summarized in Table 2.

**Table 1:** Study characteristics of the 31 studies included in the systematic review and meta-analysis

Study	Design	Geographic region	Detection method	DLBCL histologic subtype(s) included	Treatment group	Specific TT agent	No. treated with each targeted agent	Treatment regimen	Treatment frequency	Treatment cycles/duration	Response assessment Time point	Total sample size, N	TP53m sample size, N	Age, median (range)	Male, n/N (%)	III-IV stage, n/N (%)	Mipi high risk, n/N (%)	Ecog PS ≥ 2, n/N	Levated LDH, n/N	Extranodal involvement, n/N	Bone marrow involvement, n/N
**Disease stage: ND DLBCL**																					

Leroy.et al.^15^	RO	Europe	Sanger sequencing	*de novo* DLBCL only; transformed lymphoma not included; HGBL/DHL/THL NR	Chemo	NA	NA	ACVBP/NCVBP	NR	4 cycles	NR	69	16	NR	NR	30/69 (43%)	0	2/16 (13%)	10/16 (63%)	NR	0/16 (0%)
Young.et al.^16^	RO	multicenter	Sanger sequencing	*de novo* DLBCL only; DLBCL-NOS vs. HGBL/DHL/THL/transformed/other LBCL NR	Chemo	NA	NA	CHOP	NR	NR	NR	477	102	NR	NR	NR	NR	31/45 (69%)	47/59 (80%)	≥2 extranodal sites: 10/61 (16%)	NR
Stefancikova.et al.^17^	RO	Europe	Sanger sequencing	*de novo* DLBCL and transformed DLBCL; transformations from FL, CLL/SLL, MZL, low-grade lymphoma NOS, and NLPHL; HGBL/DHL/THL NR	TT + Chemo	rituximab	13/13 (100%) in p53 mutation and/or deletion subgroup; 46/46 (100%) in analyzed *de novo* R-CHOP cohort	R-CHOP	NR	NR	NR	75	16	54 (16–85)	41/75 (55%)	50/75 (67%)	36/75 (48%)	NR	NR	NR	NR
Xu-Monette.et al.^18^	RO	multicenter	NR	*de novo* DLBCL only; PMBCL, cutaneous/CNS DLBCL, HIV-associated DLBCL, and transformed lymphoma excluded; HGBL/DHL/THL NR	TT + Chemo	rituximab	111/111 (100%)	R-CHOP	q21 d	6/8 cycles	NR	506	111	NR	296/506 (58%)	249/506 (49%)	158/506 (31%)	11/100 (11%)	76/103 (74%)	≥2 extranodal sites: 21/108 (19%)	NR
Schiefer.et al.^19^	RO	Europe	Sanger sequencing	*de novo* DLBCL and BCLU; transformed lymphoma included; DHL/triple-aberrant cases included; HIV/transplant-associated lymphomas excluded	TT + Chemo	rituximab	32/32 (100%) in TP53+ subgroup; 101/101 (100%) overall	TT + Chemo	NR	NR	NR	101	32	NR	60/101 (59%)	67/101 (66%)	60/101 (59%)	NR	23/32 (72%)	27/32 (84%)	8/32 (25%)
Zenz.et al.^20^	Prospective	Europe	Sanger sequencing	NR	TT + Chemo	rituximab	33/63 (52%) in TP53-mutated subgroup; 136/265 (51%) overall analyzed cohort	R-CHOP	q14 days	6/8 cycles	NR	265	63	67 (61–79)	136/265 (51%)	NR	NR	10/63 (16%)	41/63 (65%)	extranodal involvement >1 site: 14/63 (22%)	NR
Peroja.et al.^21^	RO	Europe	Sanger sequencing	*de novo* DLBCL-NOS only; DH/TH assessed; transformed lymphoma NR	TT + Chemo	rituximab	9/9 (100%) in TP53-mutated subgroup; 155/155 (100%) overall	R-CHOP	NR	NR	NR	155	9	NR	83/155 (53%)	81/155 (53%)	18/155 (12%)	2/9 (22%)	6/9 (67%)	>1 extranodal site: 4/9 (44%)	NR
Voropaeva.et al.^41^	RO	Europe	Sanger sequencing	*de novo* DLBCL; HGBL/DHL/THL/transformed/other LBCL NR	TT + Chemo	rituximab	12/12 (100%) in TP53mut subgroup; 74/74 (100%) overall	R-CHOP	q21 d	6/8 cycles	NR	74	12	NR	35/74 (47%)	67/74 (91%)	53/74 (72%)	4/12 (33%)	7/12 (58%)	8/12 (67%)	8/12 (67%)
Qin.et al.^7^	RO	Asia	next-generation sequencing	*de novo* DLBCL; exact DLBCL-NOS vs. HGBL/DHL/THL/transformed/other LBCL NR	TT + Chemo	rituximab	44/44 (100%)	R-CHOP	NR	NR	NR	214	47	56 (18–82)	105/214 (49%)	116/214 (54%)	64/214 (30%)	NR	23/38 (61%)	30/44 (68%)	NR
Qin.et al.^8^	RO	Asia	next-generation sequencing	*de novo* DLBCL; primary CNS DLBCL, prior indolent lymphoma/transformed disease, other primary malignancies, and HIV infection excluded; HGBL/DHL/THL NR	TT + Chemo	rituximab	50/50 (100%) in TP53-mutated R-CHOP/R-CHOP-like subgroup; 164/164 (100%) in R-CHOP/R-CHOP-like survival cohort	R-CHOP	NR	NR	NR	191	59	55 (11–82)	106/191 (55%)	112/191 (58%)	70/191 (37%)	NR	NR	NR	NR
Zhang.et al.^24^	single-arm	Asia	next-generation sequencing	*de novo* DLBCL; IPI ≥2; DLBCL-NOS vs. HGBL/DHL/THL/transformed/other LBCL NR	TT + Chemo	rituximab	5/5 (100%) in TP53-mutated subgroup	DR-CHOP	NR	NR	NR	49	5	55 (25–74)	22/49 (45%)	44/49 (90%)	15/49 (31%)	NR	NR	NR	NR
Chiappella.et al.^10^	RCT	Europe	Sanger sequencing	*de novo* DLBCL only; FL IIIb/PMBCL from parent trial excluded; DTH present in 11/105 evaluable cases; transformed lymphoma NR	TT + Chemo	rituximab	11/11 (100%) in TP53-mutated subgroup; 125/125 (100%) in analyzed cohort	R-CHOP	NR	NR	After 4 cycles	125	11	51 (39–57)	59/125 (47%)	NR	21/125 (17%)	NR	NR	NR	NR
Dodero.et al.^25^	RO	Europe	next-generation sequencing	*de novo* DEL and HG-BCL/DH/TH; TP53-mutated subgroup: DEL/no rearrangement 8/16, DEL-BCL2 3/16, DEL-MYC 3/16, DEL-DH/TH 2/16	TT + Chemo	rituximab	16/16 (100%) in TP53-mutated subgroup; 122/122 (100%) overall	DA-EPOCH-R	q21 d	6 cycles	NR	122	16	59 (24–79)	75/122 (61%)	95/122 (78%)	67/122 (55%)	NR	NR	NR	NR
Zhu.et al.^27^	RCT	Asia	next-generation sequencing	*de novo* non-GCB DLBCL; ABC-enriched by GEP; DLBCL-NOS vs. HGBL/DHL/THL/transformed/other LBCL NR	TT + Chemo	ibrutinib	12/14 (86%) in TP53-mutated subgroup	ibrutinib+R-CHOP	NR	NR	NR	104	12	59 (25–77)	57/104 (55%)	69/104 (66%)	5/104 (5%)	NR	NR	>1 extranodal site reported only for overall treatment arms	NR
					TT + Chemo + Placebo	rituximab	14/14 (100%) in TP53-mutated subgroup;	R-CHOP	NR	NR	NR	96	2	59 (19–80)	58/96 (60%)	58/96 (60%)	9/104 (9%)				
Fang.et al.^29^	RO	Asia	next-generation sequencing	*de novo* DLBCL; PMBCL and primary CNS DLBCL excluded; DLBCL-NOS vsHGBL/DHL/THL/transformed/other LBCL NR	TT + Chemo	rituximab	146/146 (100%) in TP53-mutated subgroup;	R-CHOP	NR	NR	NR	576	96	53.5 (17–84)	358/576 (62%)	309/576 (53%)	193/576 (34%)	NR	NR	NR	NR
				–	TT + Chemo	decitabine	50/146 (34%) in TP53-mutated subgroup	DR-CHOP	NR	NR	NR	91	50	NR	NR	NR	NR				
Landsburg.et al.^31^	RO	America	next-generation sequencing	*de novo* DLBCL/HGBL; TP53-mutated subgroup: HGBL 13/42 (31%), prior indolent lymphoma 6/42 (14%)	TT + Chemo	rituximab	42/42 (100%) in TP53-mutated subgroup; 117/117 (100%) overall	R-CHOP/R-EPOCH	NR	NR	NR	117	42	63	NR	NR	49/117 (42%)	NR	NR	NR	NR
Zhang.et al.^33^	RCT	Asia	next-generation sequencing	*de novo* DLBCL; DLBCL-NOS vs. HGBL/DHL/THL/transformed/other LBCL NR; MYC/BCL6 rearrangement 1/128, MYC/BCL2 rearrangement 0/128	TT + Chemo	rituximab	rituximab: 21/21 (100%) in TP53mut subgroup; decitabine: 11/21 (52%) in TP53mut subgroup	R-CHOP+decitabine	q21 d	NR	NR	128	21	64 (25–74)	67/128 (52%)	99/128 (77%)	83/128 (65%)	NR	NR	≥2 extranodal sites reported only for overall cohort	NR
Jiang.et al.^36^	prospective	Asia	NR	*de novo* DLBCL; DLBCL-NOS vs. HGBL/DHL/THL/transformed/other LBCL NR	TT	zanubrutinib; rituximab	for reported TP53-mutated ZR2 subgroup: zanubrutinib + rituximab + lenalidomide 5/5 (100%); overall: ZR2 30/90 (33%), R-CHOP-like 60/90 (67%)	BTKi+R2	q21 d	6 cycles	After 4/6 cycles	90	5	71 (65–94)	56/90 (62%)	51/90 (56%)	42/90 (46%)	NR	NR	≥2 extranodal sites reported only by treatment arm	NR
Jin.et al.^37^	RO	Asia	next-generation sequencing	*de novo* DLBCL; DLBCL-NOS vs. HGBL/DHL/THL/transformed/other LBCL NR	TT + Chemo	rituximab	TP53-mutated subgroup: likely 44/44 (100%) received R-CHOP/R-CHOP-like, but exact treatment denominator not separately reported	R-CHOP	q21 d	NR	NR	123	44	57 (12–83)	66/123 (53%)	69/123 (56)	50/123 (41)	NR	NR	NR	NR
Chen.et al.^40^	RO	Asia	next-generation sequencing	*de novo* DLBCL; primary CNS/cutaneous DLBCL, PMBCL, EBV-positive DLBCL, DLBCL-NOS vs. HGBL/DHL/THL/transformed LBCL NR	TT + Chemo	rituximab	170/170 (100%) in all TP53-altered cases; 139/139 (100%) in TP53-mutant/CNL clinical-data subgroup	R-CHOP/R-EPOCH	NR	NR	NR	551	139	56 (16–82)	297/551 (54%)	268/551 (48%)	144/551 (26%)	11/139 (8%)	50/138 (36%)	primary extranodal site: 87/139 (63%); ≥2 extranodal sites: 35/139 (25%)	NR

**Disease stage: rr DLBCL**																					

Porpaczy.et al.^22^	RO	Europe	next-generation sequencing	R/R DLBCL and DHL/THL; transformed lymphoma included; CAR-T cohort included DHL/THL 5/29 and transformed lymphoma 5/29	CAR-T	NA	NA	anti-CD19 CAR-T	NR	NR	NR	29	10	55 (31–79)	NR	10/29 (34%)	NR	NR	NR	overall cohort extranodal involvement: 109/170 (64%)	NR
					TT + Chemo	zanubrutinib	NR	salvage therapy without CAR-T	NR	NR	NR	141	31	60 (19–95)	NR	97/141 (69%)	NR	NR	NR	–	NR
Wilson.et al.^23^	single-arm	America	next-generation sequencing	R/R DLBCL; part 1 allowed transformed DLBCL/coexisting histologies/PMBCL; part 2 restricted to non-GCB DLBCL; RP2D cohort included *de novo* 24/26, transformed 1/26, PMBCL 1/26; HGBL/DHL/THL NR	TT + Chemo	ibrutinib;rituximab	ibrutinib 35/35 (100%), rituximab/DA-EPOCH-R 35/35 (100%), lenalidomide 32/35 (91%); RP2D: ibrutinib + lenalidomide + DA-EPOCH-R 26/26 (100%); reported TP53-mutated cases in mutation table: 6/6 received ibrutinib + lenalidomide + DA-EPOCH-R	ibrutinib + Lenalidomide + DA-EPOCH-R	q21 d	6 cycles	NR	35	5	57.5 (28–79)	NR	NR	NR	NR	NR	NR	NR
Yuan.et al.^11^	RO	Asia	next-generation sequencing	R/R CD20-positive DLBCL; double-hit status 4/27; CNS lymphoma, HIV-positive DLBCL, PTLD, and prior BTKi exposure excluded; transformed/HGBL/THL/other LBCL NR	TT + Chemo	zanubrutinib	zanubrutinib: 27/27 (100%); rituximab: 18/27 (67%); in TP53-mutated poor-responder subgroup: zanubrutinib 7/7 (100%) and CD19-CAR-T 7/7 (100%)	BTKi+TT + Chemo	NR	NR	NR	27	7	59 (15–72)	20/27 (74%)	20/27 (74%)	NR	overall cohort: 15/27 (56%)	overall cohort: 19/27 (70%)	overall cohort: 18/27 (67%)	NR
Shouval.et al.^26^	RO	America	next-generation sequencing	R/R LBCL; transformed FL, other transformed low-grade lymphoma, and HGBL included; DHL/THL present; TP53-altered subgroup histologic breakdown NR	CAR-T	NA	NA	anti-CD19 CAR-T	NR	NR	After 28/90 days	153	30	66 (57–72)	101/153 (66%)	106/153 (70%)	NR	NR	NR	NR	NR
Wei.et al.^28^	single-arm	Asia	next-generation sequencing	R/R aggressive B-NHL; TP53-altered subgroup included DLBCL-NOS, transformed FL, HGBL DH/TH, HGBL-NOS, Burkitt lymphoma, MCL, and other B-cell lymphoma subtypes	CAR-T	NA	NA	anti-CD19/22 CAR-T	NR	NR	NR	65	31	NR	43/65 (66%)	56/65 (86%)	NR	22/60 (37%)	40/60 (67%)	NR	NR
Gao.et al.^30^	RO	Asia	next-generation sequencing	R/R DLBCL; transformed lymphoma and PMBCL excluded; DHL/THL excluded from TP53mut survival analyses; DLBCL-NOS vs. HGBL-NOS NR	CAR-T	NA	NA	CAR-T	NR	NR	NR	96	46	NR	49/96 (51%)	85/96 (88%)	73/96 (76%)	Overall TP53mut: 13/40 (33%); CAR-T TP53mut: 11/32 (34%)	NR	NR	NR
Qin.et al.^32^	RO	Asia	next-generation sequencing	R/R DLBCL; CNS/testicular/mediastinal lymphoma excluded; GCB 14/36, non-GCB 22/36; DHL/THL/transformed lymphoma NR	PD-1 +TT	rituximab + PD-1 monoclonal antibody: toripalimab, sintilimab, pembrolizumab, or nivolumab	overall: rituximab + PD-1 mab 36/36 (100%); PD-1 agents: toripalimab 27/36 (75%), sintilimab 4/36 (11%), pembrolizumab 3/36 (8%), nivolumab 2/36 (6%); TP53-mutated NGS subgroup: 13/13 (100%) received PD-1 mab + rituximab	PD-1 +TT	q21 d	6 cycles	After 6 cycles	36	13	62 (27–78)	22/36 (61%)	24/36 (66%)	19/36 (53%)	overall cohort: 15/34 (44%) evaluable, or 15/36 (42%) including unknowns	overall cohort: 18/32 (56%) evaluable, or 18/36 (50%) including unknowns	overall cohort: any extranodal involvement 30/36 (83%); ≥2 extranodal organs 18/36 (50%)	NR
Shi.et al.^34^	RO	Asia	Next-generation sequencing	R/R DLBCL; transformed FL/MALT/CLL included; DH/TH included; GCB 31/105 and non-GCB 74/105	CAR-T	NA	NA	anti-CD19 CAR-T	NR	NR	NR	80	40	49 (13–79)	40/80 (50%)	71/80 (88%)	NR	overall cohort: 52/105 (50%)	overall cohort: 74/96 (77%)	overall cohort reported >1 extranodal site: 50/105 (48%)	overall cohort: 13/103 (13%)
Xue.et al.^35^	RO	Asia	Next-generation sequencing	R/R DLBCL; DEL/TP53 stratified; DLBCL-NOS vs. HGBL/DHL/THL/transformed lymphoma NR	CAR-T	NA	NA	anti-CD19 CAR-T	NR	NR	NR	73	28	59 (49–67)	36/73 (49%)	63/73 (86%)	28/73 (38%)	10/28 (36%)	NR	≥2 extranodal organs: 15/28 (54%)	NR
Xu.et al.^38^	RO	Asia	NR	R/R DLBCL; GCB 2/17, non-GCB 15/17; HGBL/DHL/THL/transformed lymphoma NR	CAR-T	zanubrutinib + anti-CD19 CAR T cell therapy; some patients also received ASCT + CAR-T	overall: zanubrutinib + anti-CD19 CAR-T 17/17 (100%); TP53-altered subgroup: zanubrutinib + anti-CD19 CAR-T 6/6 (100%)	anti-CD19 CAR-T	NR	NR	NR	17	6	43 (25–69)	12/17 (71%)	16/17 (94%)	12/17 (71%)	overall cohort: 4/17 (24%)	overall cohort: 12/17 (71%)	NR	overall cohort: 4/17 (24%)
Xue.et al.^39^	RO	Asia	next-generation sequencing	R/R DLBCL; GCB 2/17, non-GCB 15/17; HGBL/DHL/THL/transformed lymphoma NR	CAR-T	zanubrutinib + anti-CD19 CAR T cell therapy; subset received ASCT + CAR-T	overall: zanubrutinib + anti-CD19 CAR-T 17/17 (100%); TP53-altered subgroup: 6/6 (100%)	anti-CD20 CAR-T	NR	NR	NR	19	7	54 (40–72)	11/19 (58%)	17/19 (90%)	14/19 (74%)	overall cohort: 4/17 (24%)	overall cohort: 12/17 (71%)	NR	overall cohort: 4/17 (24%)

Note: NA: not applicable; NR: not reported.

**Table 2:** Treatment outcomes of the 31 studies included in the systematic review and meta-analysis

Study	Treatment group	CR rate, n/N (%)	ORR, n/N (%)	OS		PFS	
				Median (95% CI)	OS rate, n/N (%)	Median (95% CI)	PFS rate, n/N (%)
**Disease stage: ND DLBCL**							

Leroy.et al.^15^	Chemo	11/16 (69%)	NR	NR	6-year OS 7/16 (44%)	NR	NR
Young.et al.^16^	Chemo	34/60 (57%)	44/60 (73%)	15.6 m (NR)	5-year OS 14/60 (23%)	NR	NR
Stefancikova.et al.^17^	TT + Chemo	NR	NR	NR	NR	NR	2-year PFS 5/16 (31%)
Xu-Monette.et al.^18^	TT + Chemo	NR	90/111 (81%)	52.9 m (NR)	5-year OS 53/111 (48%)	51.95 m(NR)	5-year PFS 51/111 (46%)
Schiefer.^19^	TT + Chemo	32/101 (32%)	NR	12 m (NR)	NR	NR	NR
Zenz.et al.^20^	TT + Chemo	39/63 (62%)	NR	NR	3-year OS 31/63 (49%)	NR	3-year PFS 26/63 (41%)
Peroja.et al.^21^	TT + Chemo	NR	6/9 (67%)	NR	3-year OS 6/9 (67%)	NR	3-year PFS 6/9 (67%)
Voropaeva.et al.^41^	TT + Chemo	7/12 (58%)	NR	NR	3-year OS 5/12 (42%)	NR	NR
Qin.et al.^7^	TT + Chemo	NR	NR	46 m (12.3–79.7)	NR	9 m (5–13)	NR
Qin.et al.^8^	TT + Chemo	NR	NR	NR	5-year OS 27/59 (46%)	NR	5-year PFS 15/59 (25%)
Zhang.et al.^24^	TT + Chemo	5/5 (100%)	5/5 (100%)	NR	NR	NR	NR
Chiappella.et al.^10^	TT + Chemo	4/11 (36%)	NR	NR	5-year OS 4/11 (36%)	NR	5-year PFS 3/11 (27%)
Dodero.et al.^25^	TT + Chemo	NR	NR	NR	2-year OS 10/16 (63%)	NR	2-year PFS 9/16 (56%)
Zhu.et al.^27^	TT + Chemo	10/12 (83%)	NR	NR	NR	NR	NR
	TT + Chemo + Placebo	1/2 (50%)	NR	NR	NR	NR	NR
Fang.et al.^29^	TT + Chemo	NR	NR	NR	3-year OS 62/96 (65%)	NR	3-year PFS 44/96 (46%)
	TT + Chemo	NR	NR	NR	3-year OS 40/50 (80%)	NR	3-year PFS 36/50 (72%)
Landsburg.et al.^31^	TT + Chemo	23/42 (55%)	30/42 (71%)	NR	2-year OS 29/42 (69%)	NR	2-year PFS 24/42 (57%)
Zhang.et al.^33^	TT + Chemo	15/21 (71%)	NR	NR	NR	NR	NR
Jiang.et al.^36^	TT	3/5 (60%)	NR	NR	NR	NR	NR
Jin.et al.^37^	TT + Chemo	14/28 (50%)	21/28 (75%)	26.2 m (NR)	NR	19.4 m (NR)	NR
Chen.et al.^40^	TT + Chemo	95/139 (68%)	NR	NR	NR	NR	NR

**Disease stage: rr DLBCL**							

Porpaczy.et al.^22^	CAR-T	NR	NR	30.9 m (NR)	NR	NR	NR
	TT + Chemo	NR	NR	12.6 m (9.4–15.9)	NR	NR	NR
Wilson.et al.^23^	TT + Chemo	1/5 (20%)	3/5 (60%)	NR	NR	NR	NR
Yuan.et al.^11^	TT + Chemo	4/7 (57%)	6/7 (85.6%)	NR	NR	NR	NR
Shouval.et al.^26^	CAR-T	10/29 (34%)	NR	NR	1-year OS 12/29 (41%)	NR	NR
Wei.et al.^28^	CAR-T	14/31 (45%)	27/31 (87%)	NR	1-year OS 20/31 (65%)	NR	1-year PFS 17/31 (55%)
Gao.et al.^30^	CAR-T	7/32 (22%)	18/32 (56%)	24.5 m (NR)	2-year OS 17/32 (53%)	6.8 m (NR)	NR
Qin.et al.^32^	PD-1 +TT	3/36 (8%)	19/36 (53%)	19.6 m (12.7–26.5)	NR	2.8 m (0.89–4.71)	NR
Shi.et al.^34^	CAR-T	13/40 (33%)	40/40 (100%)	NR	1-year OS 20/40 (50%)	NR	NR
Xue.et al.^35^	CAR-T	NR	12/28 (43%)	NR	NR	NR	NR
Xu.et al.^38^	CAR-T	NR	NR	27 m (NR)	NR	12 m (NR)	NR
Xue.et al.^39^	CAR-T	6/7 (86%)	NR	NR	2-year OS 5/7 (71%)	NR	2-year PFS 5/7 (71%)

Note: NR: not reported.

### TP53-related molecular profiles

*TP53*-related information was extracted from all included studies or eligible cohorts to clarify the operational definition and molecular heterogeneity of TP53m disease (Table S1). All 31 studies/cohorts provided an operational definition of TP53m disease, although these definitions varied. Information on variant classes, including missense, nonsense, frameshift, splice-site, truncating, or in-frame variants, was reported in 24 studies. Details regarding DNA-binding-domain involvement, mutation location, or hotspot residues were reported in 19 studies, while functional or pathogenicity annotation was available in 17 studies. In contrast, VAF information or VAF thresholds were reported in only 3 studies, and clonal/subclonal status was fully or partially described in 2 studies. *TP53* deletion, 17p loss, loss of heterozygosity (LOH), or copy-number loss were reported in 10 studies. Data regarding biallelic or multi-hit *TP53*-related information were available in 15 studies. However, only 2 studies explicitly defined or reported biallelic *TP53* aberrations. Furthermore, cell-of-origin (COO) subtype was reported in 28 studies, double-hit lymphoma/triple-hit lymphoma (DHL/THL) status in 17 studies, double-expressor status in 17 studies, and relevant co-mutations or broader genomic alterations in 22 studies.

### Quality assessment of included studies

Results of the risk-of-bias assessment for each included study are presented in Figure S1 and Table S2. Among the three RCTs assessed using the revised Cochrane risk-of-bias tool for randomized trials (RoB 2), one study was judged to have a low overall risk of bias,^33^ whereas two studies were rated as having some concerns. These concerns were mainly related to incomplete reporting of the randomization process, deviations from intended interventions, or selection of the reported result. Among the 28 non-randomized studies assessed using the risk of bias in non-randomized studies of interventions tool (ROBINS-I), four studies were judged to have a high overall risk of bias,^8^^,^^19^^,^^24^^,^^41^ mainly due to inadequate control of confounding, potential selection bias in participant inclusion, incomplete reporting of missing data or follow-up, and/or limitations in outcome measurement or selective reporting. The remaining non-randomized studies were judged to have a moderate overall risk of bias, although several exhibited domain-specific concerns. Any discrepancies were resolved through discussions or by consulting a third reviewer (NS).

### Meta-analysis

#### Complete remission

**Figure 2:**
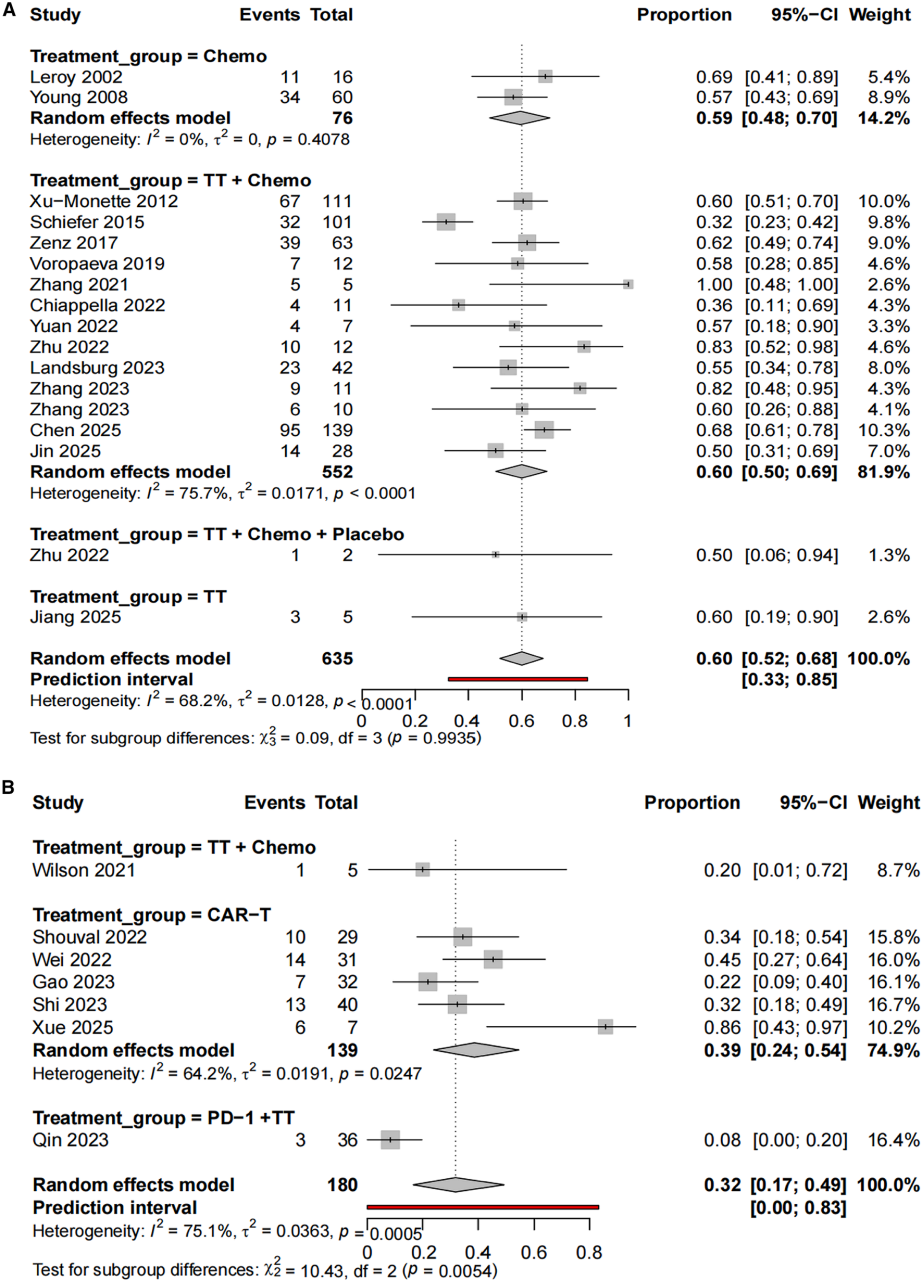
CR rate of newly diagnosed or relapsed/refractory patients with TP53m DLBCL treated with current remission strategies (A) CR rate of newly diagnosed patients with TP53m DLBCL. (B) CR rate of relapsed/refractory patients with TP53m DLBCL. Squares represent the estimated proportions from individual studies, with the size of each square indicating the corresponding study weight. Horizontal lines indicate 95% confidence intervals (CIs). Diamonds represent pooled estimates derived from random-effects models, and red lines indicate prediction intervals. Statistical heterogeneity was assessed using the I² statistic and Cochran’s Q test.

Fifteen studies reported data on complete remission (CR) for individuals with ND TP53m DLBCL. The aggregated CR rate under current therapeutic approaches was 60% (95% CI: 52%–68%; 95% prediction interval, 33%–85%; *n* = 635; *I*^2^ = 68.2%) (Figure 2A). Two studies reported outcomes among individuals receiving chemotherapy (Chemo) alone, with a CR rate of 59% (45/76) (95% CI: 48%–70%; *n* = 76). The pooled CR rate among individuals receiving TT + Chemo was 60% (95% CI: 50%–69%; *n* = 552; *I*^2^ = 75.7%). One study^11^ documented a CR rate of 60% (3/5) in patients receiving TT, BTKi combined with rituximab (anti-CD20), and lenalidomide (95% CI: 19%–90%; *n* = 5). Sensitivity analysis utilizing a leave-one-out approach (Figure S2A) showed that no individual study exerted significant effects on the aggregated CR rate. Egger’s test (*p* = 0.4798) indicated no significant publication bias (Figure S2B). No heterogeneity was observed across subgroups stratified by study design, region, sample size, or methods for detecting *TP53* mutations (Figures S3–S6).

Seven studies reported data on CR for individuals with R/R TP53m DLBCL. The aggregated CR rate with current therapeutic approaches was 32% (95% CI: 17%–49%; 95% prediction interval, 0%–83%; *n* = 180; *I*^2^ = 75.1%) (Figure 2B). The CR rate in individuals receiving CAR-T was 39% (95% CI: 24%–54%; *n* = 139; *I*^2^ = 64.2%). One study documented a CR rate of 20% (1/5) (95% CI: 1%–72%; *n* = 5) among individuals receiving TT + Chemo. Another study documented a CR rate of 8% (3/36) (95% CI: 0–20%; *n* = 36) among individuals receiving programmed cell death protein 1 (PD-1) inhibitor + TT. Sensitivity analysis using a leave-one-out approach (Figure S7) showed that no individual study exerted significant effects on the aggregated CR rate. No heterogeneity was observed across subgroups stratified by study design, region, or sample size (Figures S8–S10).

#### Overall remission rate

**Figure 3:**
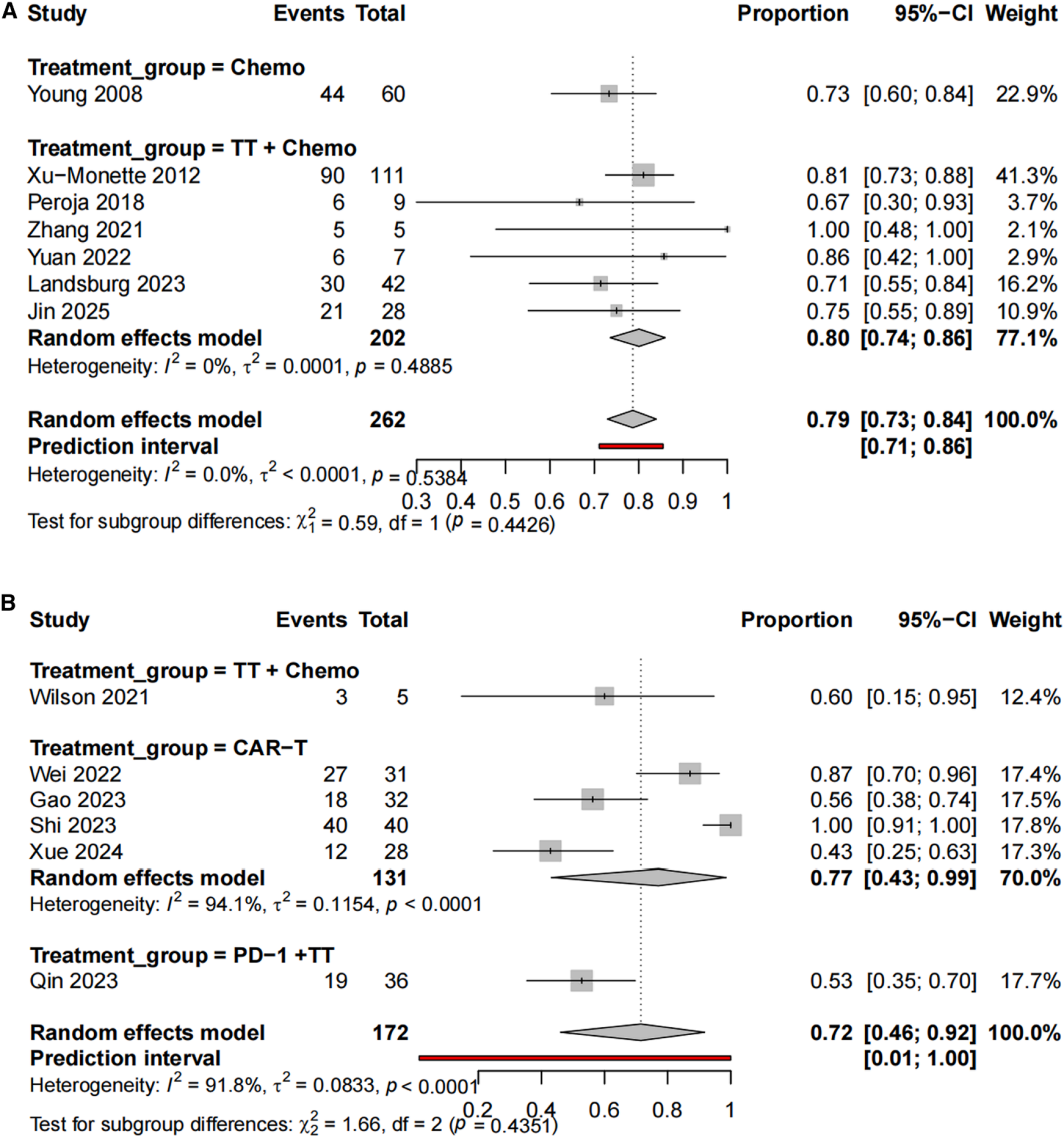
ORR of newly diagnosed or relapsed/refractory patients with TP53m DLBCL treated with current remission strategies (A) ORR of newly diagnosed patients with TP53m DLBCL. (B) ORR of relapsed/refractory patients with TP53m DLBCL. Squares represent the estimated proportions from individual studies, with the size of each square indicating the corresponding study weight. Horizontal lines indicate 95% confidence intervals (CIs). Diamonds represent pooled estimates derived from random-effects models, and red lines indicate prediction intervals. Statistical heterogeneity was assessed using the I² statistic and Cochran’s Q test.

Seven studies reported data on overall remission rate (ORR) for individuals with ND TP53m DLBCL. Solely one study documented an ORR of 73% (44/60) (95% CI: 60%–84%; *n* = 60) among individuals receiving Chemo alone. The remaining six studies evaluated Chemo combined with TT, and the ORR was 80% (95% CI: 74%–86%; *n* = 202; *I*^2^ = 0) (Figure 3A). Among TT + Chemo regimens, combined anti-CD20 and BTKi showed a relatively high ORR in the available studies. In one study, anti-CD20 + CD38 monoclonal antibody daratumumab achieved an ORR of 100% (95% CI: 48%–100%; *n* = 5). Sensitivity analysis using a leave-one-out approach (Figure S11) showed that no individual study exerted significant effects on the aggregated ORR. No heterogeneity was observed across subgroups stratified by study design, region, sample size, or methods for detecting *TP53* mutations (Figures S12–S15).

Six studies reported data on ORR for individuals with R/R TP53m DLBCL (Figure 3B). The aggregated ORR of TP53m DLBCL under current therapeutic approaches was 72% (95% CI: 46%–92%; *n* = 172; *I*^2^ = 91.8%). The ORR of individuals receiving CAR-T was 77% (95% CI: 43%–99%; *n* = 131; *I*^2^ = 94.1%). One study documented a pooled ORR of 60% (95% CI: 15%–95%; *n* = 5) among individuals receiving TT combined with Chemo. Another study documented an ORR of 53% (19/36) (95% CI: 35%–70%; *n* = 36) among individuals receiving PD-1 inhibitor combined with TT. Sensitivity analysis using a leave-one-out approach (Figure S16) showed that no individual study exerted significant effects on the aggregated ORR. No heterogeneity was observed across subgroups stratified by study design, region, or sample size (Figures S17–S19).

#### Overall survival

**Figure 4:**
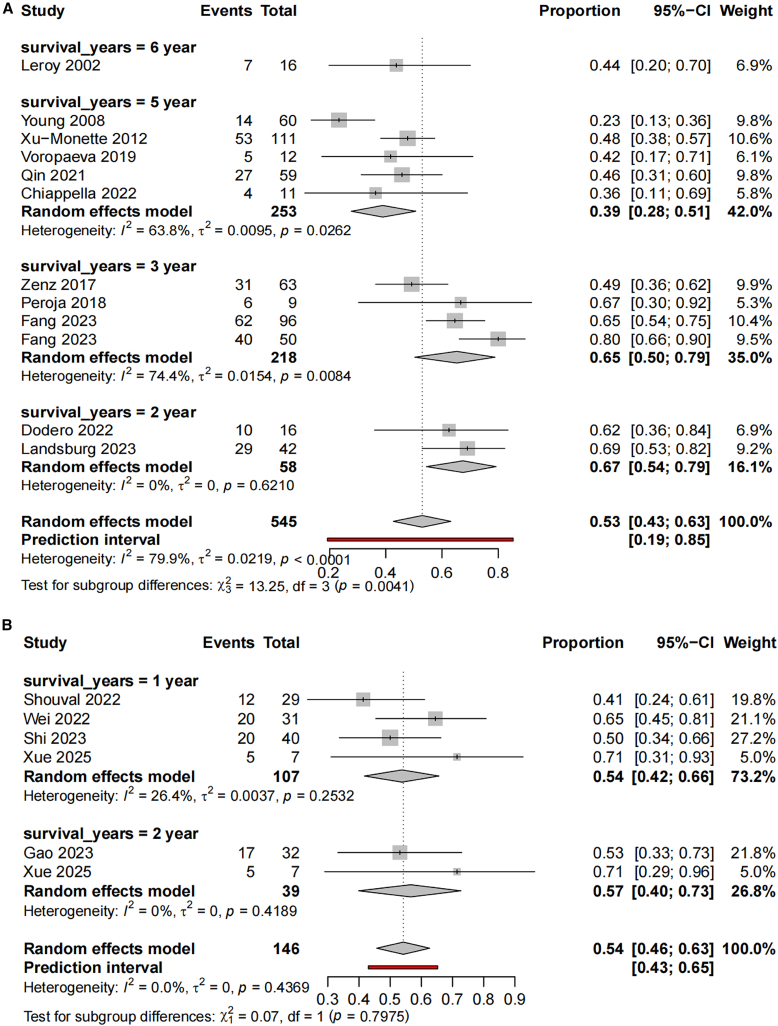
OS rate of newly diagnosed or relapsed/refractory patients with TP53m DLBCL treated with current remission strategies (A) OS rate of newly diagnosed TP53m DLBCL. (B) OS rate of relapsed/refractory TP53m DLBCL. Squares represent the estimated proportions from individual studies, with the size of each square indicating the corresponding study weight. Horizontal lines indicate 95% confidence intervals (CIs). Diamonds represent pooled estimates derived from random-effects models, and red lines indicate prediction intervals. Statistical heterogeneity was assessed using the I² statistic and Cochran’s Q test.

For individuals with ND TP53m DLBCL, five studies reported mean survival times, ranging from 12 to 52.9 months. However, only one study documented data on median OS for the TT + Chemo regimen. The median OS was 46 months (95% CI: 12.26–79.74; *n* = 47). Two studies documented data on the 2-year OS rate among individuals with ND TP53m DLBCL receiving TT + Chemo, with a 2-year OS rate of 67% (95% CI: 54%–79%; *n* = 58; *I*^2^ = 0%). Three studies documented data on the 3-year OS rate among individuals with ND TP53m DLBCL receiving TT + Chemo, with a 3-year OS rate of 65% (95% CI: 50%–79%; *n* = 218; *I*^2^ = 74.4%). Five studies documented data on the 5-year OS rate among individuals with ND TP53m DLBCL receiving TT + Chemo, with a 5-year OS rate of 39% (95% CI: 28%–51%; *n* = 253; *I*^2^ = 63.8%) (Figure 4A). Solely the study by Leroy provided a 6-year OS rate of 44% (95% CI: 20%–70%; *n* = 16) for individuals receiving Chemo alone. Sensitivity analysis using a leave-one-out approach (Figure S20A) showed that no individual study exerted significant effects on the aggregated OS rate. Egger’s test (*p* = 0.9847) indicated no significant publication bias (Figure S20B). Significant heterogeneity was observed across subgroups stratified by region and methods for detecting *TP53* mutations. However, no heterogeneity was observed across subgroups stratified by study design, sample size, or treatment regimen (Figures S21–S25).

For individuals with R/R TP53m DLBCL, two studies documented data on median OS in the TT regimen. Porpaczy et al.^22^ reported a median OS of 12.26 months (95% CI: 9.35–15.85; *n* = 31) in the salvage treatment group excluding CAR-T. Qin et al.^32^ reported a median OS of 19.6 months (95% CI: 12.69–26.51; *n* = 36) among individuals receiving PD-1 inhibitor + TT. Four studies documented data on the 1-year OS rate among individuals with R/R TP53m DLBCL receiving CAR-T. Pooled analysis exhibited a 1-year OS rate of 54% under current therapeutic approaches (95% CI: 42%–66%; *n* = 107; *I*^2^ = 26.4%). Two studies documented data on the 2-year OS rate among individuals with R/R TP53m DLBCL receiving CAR-T. The aggregated 2-year OS rate under current therapeutic approaches was 57% (95% CI: 40%–73%; *n* = 146; *I*^2^ = 0%) (Figure 4B). Sensitivity analysis using a leave-one-out approach (Figure S26) exhibited that no individual study exerted significant effects on the aggregated OS rate. No heterogeneity was observed across subgroups stratified by study design, region, or sample size (Figures S27–S29).

#### Progression-free survival

**Figure 5:**
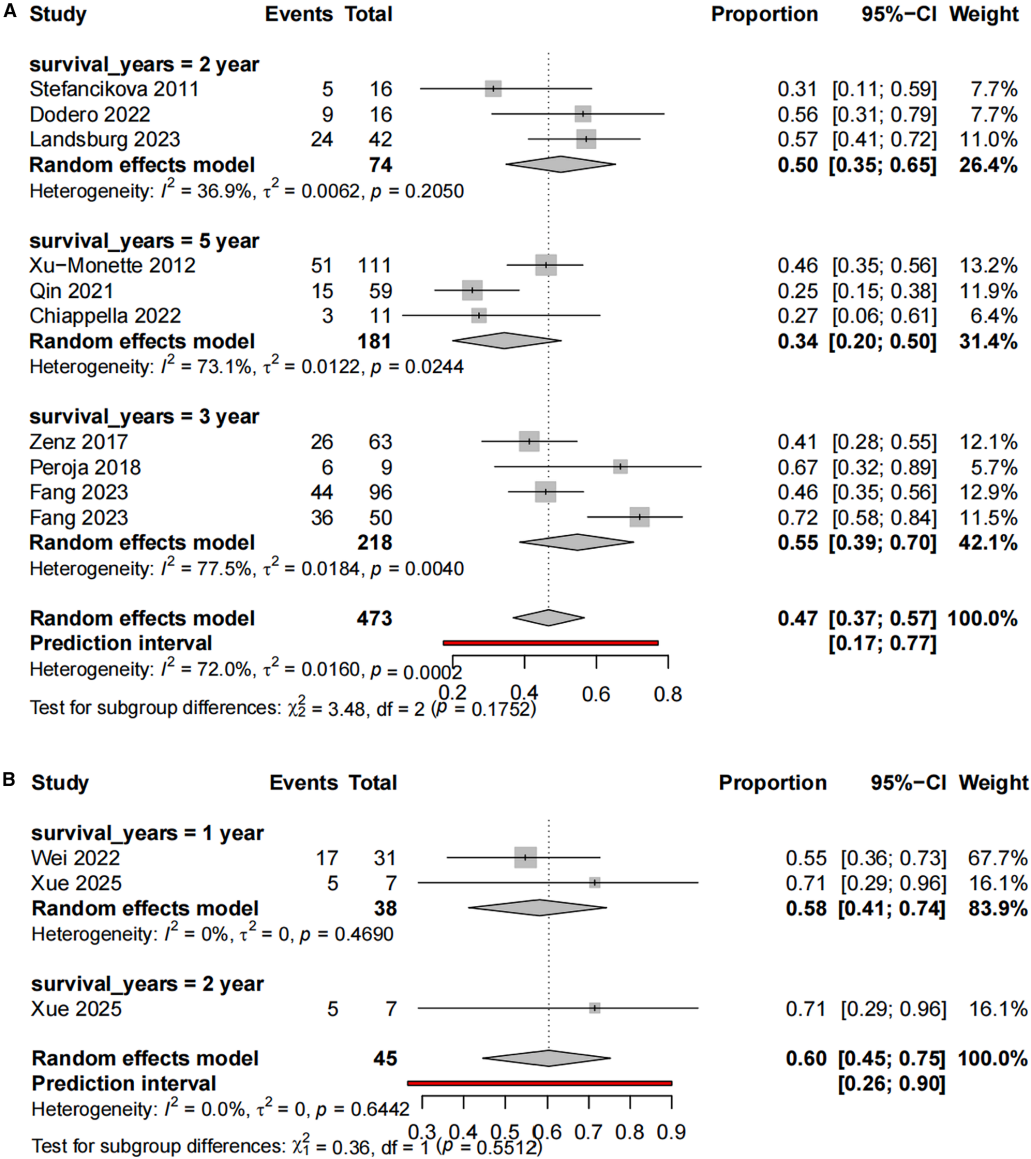
PFS rate of newly diagnosed or relapsed/refractory patients with TP53m DLBCL treated with current remission strategies (A) PFS rate of newly diagnosed TP53m DLBCL. (B) OS rate of relapsed/refractory TP53m DLBCL. Squares represent the estimated proportions from individual studies, with the size of each square indicating the corresponding study weight. Horizontal lines indicate 95% confidence intervals (CIs). Diamonds represent pooled estimates derived from random-effects models, and red lines indicate prediction intervals. Statistical heterogeneity was assessed using the I² statistic and Cochran’s Q test.

For individuals with ND TP53m DLBCL, one study provided data on PFS in the TT + Chemo regimen, with a median PFS of 9 months (95% CI 5–13; *n* = 47). Three studies reported 2-year PFS among individuals with ND TP53m DLBCL receiving TT + Chemo. Pooled analysis exhibited a 2-year PFS rate of 50% (95% CI 35%–65%; *n* = 74; *I*^2^ = 36.9%). Three studies reported 3-year PFS among individuals with ND TP53m DLBCL receiving TT + Chemo, with an aggregated 3-year PFS rate of 55% (95% CI 39%–70%; *n* = 218; *I*^2^ = 77.5%). Three studies reported 5-year PFS among individuals with ND TP53m DLBCL receiving TT + Chemo, with an aggregated 5-year PFS rate of 34% (95% CI 20%–50%; *n* = 181; *I*^2^ = 73.1%) (Figure 5A). Sensitivity analysis using a leave-one-out approach (Figure S30A) showed that no individual study exerted significant effects on the aggregated PFS rate. Egger’s test (*p* = 0.8602) indicated no significant publication bias (Figure S30B). No heterogeneity was observed across subgroups stratified by study design, sample size, region, or methods for detecting *TP53* mutations (Figures S31–S34).

For individuals with R/R TP53m DLBCL, one study provided data on PFS in the PD-1 inhibitor + TT regimen. The aggregated median PFS was 2.80 months (95% CI 0.89–4.71; *n* = 36). One study documented the 1-year PFS rate among individuals with R/R TP53m DLBCL receiving CAR-T. The one-year PFS rate was 71% (95% CI 29%–96%; *n* = 7). Two studies documented the 2-year PFS rate among individuals with R/R TP53m DLBCL receiving CAR-T, with a PFS rate of 58% (95% CI 41%–74%; *n* = 38; *I*^2^ = 0%) (Figure 5B). Sensitivity analysis using a leave-one-out approach (Figure S35) showed that no individual study exerted significant effects on the aggregated PFS rate. No heterogeneity was observed across subgroups stratified by study design and sample size (Figures S36–S37).

## Discussion

Despite the substantial advances achieved in the treatment of DLBCL, the subgroup of patients harboring *TP53* mutations remains a major clinical challenge in terms of prognosis. This SRMA comprehensively integrates the available evidence and evaluates the efficacy of multiple therapeutic strategies among individuals with ND and R/R TP53m DLBCL.

Pooled data indicate that TT + Chemo achieved a CR rate of 60% and an ORR of 80% in patients with ND TP53m DLBCL. Though these remission rates appear moderate, the long-term survival outcomes are particularly noteworthy. The 2-, 3-, and 5-year OS rates were 67%, 65%, and 39%, respectively. The available 2- and 3-year PFS rates were 50% and 55%, respectively. Relative to historical cohorts receiving standard Chemo, our results suggest that TT + Chemo needs to be further investigated. Nevertheless, outcomes remain suboptimal relative to the general DLBCL population. Combining novel agents (like BTKi, BCL-2i, or other molecularly targeted agents) with Chemo appears to deepen initial remission and may partially overcome TP53 inactivation-mediated mechanisms of resistance, thereby achieving more durable disease control.^42^^,^^43^ Exploratory studies have also been conducted at the epigenetic level.^44^ In this context, an exploratory cohort study of TP53m DLBCL reports that 12 patients treated with R-CHOP plus azacitidine achieve a complete response rate of 91.7%,^45^ providing preliminary support for biomarker-guided combination strategies in this ultra-high-risk population. This is particularly important given the interaction between this mutation and COO subtypes. Though the frequency of TP53 mutations is comparable between ABC and GCB-DLBCL,^46^ they may exert distinct biological and prognostic effects through subtype-specific oncogenic programs. In a large retrospective cohort published by Zhang et al.^45^ in 2026, *TP53* mutations are associated with significantly inferior OS and PFS in GCB-DLBCL, whereas no significant prognostic effect is observed in non-GCB disease. These findings suggest that the clinical impact of *TP53* mutations is context-dependent and should be interpreted within the molecular background of the tumor.

However, in-depth analysis of long-term data reveals the persistent challenge posed by this disease. In patients with ND TP53m DLBCL, the OS rate declined from 67% at 2 years to 39% at 5 years. Meanwhile, similar decrease was observed in the PFS rate. This finding indicates that despite favorable initial efficacy, most patients ultimately experience disease progression and subsequent death. This pattern suggests that, while TT + Chemo can induce relatively high initial response rates, it might fail to eradicate minimal residual disease or prevent late relapse, which is propelled by the severe genomic instability associated with TP53 loss.^47^ Therefore, it is necessary to adopt consolidation strategies or implement maintenance therapy after induction treatment, thereby improving the durability of remission in these high-risk populations.^48^^,^^49^^,^^50^ Previous studies on high-risk DLBCL have indicated that allo-HSCT may be explored as an intensive therapeutic option for individuals with TP53m DLBCL who exhibit high risks of relapse after induction or salvage therapy but have achieved measurable tumor reduction and remain eligible for intensive therapy.^51^

In the therapeutic context of R/R DLBCL, our findings support CAR-T-based approaches as an important treatment strategy for R/R TP53m DLBCL based on available observational evidence. This analysis shows a pooled CR rate of 39% with CAR-T, while the ORR reached a promising 77%. The 2-year OS and PFS rates were 57% and 58%. Given the historically dismal prognosis of R/R TP53m DLBCL, conventional salvage Chemo offers only suboptimal and transient benefit. Unlike conventional Chemo, which directly induces DNA damage, CAR-T activity is initiated through antigen recognition.^52^ Nevertheless, CAR-T efficacy in TP53m DLBCL cannot be considered simply independent of TP53-related resistance biology, as downstream tumor-cell killing and resistance may be shaped by antigen density, antigen loss, apoptotic competence, immune-escape pathways, the tumor microenvironment, and CAR-T expansion and persistence.^26^^,^^53^

Nevertheless, the therapeutic outcomes of CAR-T require cautious interpretation. A CR rate of 39% indicates that most patients still fail to achieve complete metabolic remission, and a high ORR of 77% does not consistently translate into long-term survival for all patients. Furthermore, the substantial heterogeneity detected in the ORR analysis (I^2^ = 94.1%) suggests marked variations in treatment efficacy. This might be attributed to factors like prior lines of therapy, tumor burden, and features of the CAR-T products that were not consistently reported across studies.^54^^,^^55^ In a single-center study, Shouval et al.^26^ report that *TP53* alterations are associated with inferior CR and OS after CD19-directed CAR-T therapy in DLBCL The 1-year OS was 44% in *TP53*-altered disease compared to 76% in *TP53* wild-type disease. By contrast, Porpaczy et al.^22^ have found that the adverse prognostic effect of *TP53* mutation observed with conventional therapy is not clearly retained among patients receiving CAR-T therapy. Dual-target CAR-T may represent a potential strategy in this setting, as Wei et al.^28^ have reported an ORR of 87.1%, a CR rate of 45.2%, and an estimated 24-month OS of 56.3% with CD19/CD22 CAR-T therapy in *TP53*-altered R/R aggressive B cell lymphoma. Subsequent long-term follow-up has suggested that dual CD19/CD22 CAR-T, particularly when integrated with sequential ASCT, may provide durable disease control in selected high-risk patients.^56^ Additional molecular features, including *TP53*/DDX3X co-mutation.^30^^,^^34^ and combined double-expression status with *TP53* alterations,^35^ may further modify CAR-T responsiveness. These findings indicate that TP53m DLBCL is biologically heterogeneous and should not be regarded as a uniform therapeutic entity. Consequently, it is necessary to adopt novel strategies to enhance its efficacy, such as combining with other targeted agents,^57^ developing dual-target CAR-T,^58^ or implementing post-CAR-T consolidation to eradicate residual disease.^59^^,^^60^ Within this framework, for individuals with TP53m DLBCL achieving partial remission or CR after CAR-T yet maintaining an extremely high biological risk of relapse, allo-HSCT can also be explored as the consolidation or salvage strategy after CAR-T if organ function is adequate and a suitable donor is available.^61^

Recent advances in DLBCL treatments serve as a crucial reference for interpreting outcomes in TP53m disease. In the frontline setting, POLARIX has established pola-R-CHP as a relevant comparator to R-CHOP, whereas ZUMA-7, TRANSFORM, and BELINDA have redefined the role of second-line CD19-directed CAR-T therapy in early R/R LBCL. Notably, discrepant trial results highlight the influence of product characteristics, trial design, bridging therapy, disease kinetics, and patient selection.^12^^,^^62^^,^^63^ ZUMA-12 has further extended CAR-T therapy into the frontline setting for high-risk LBCL.^14^ Concurrently, clinical trials of CD20×CD3 bispecific antibodies, including glofitamab, epcoritamab, odronextamab, and mosunetuzumab, provide additional insights for heavily pretreated R/R DLBCL.^13^^,^^64^^,^^65^ However, detailed outcomes for TP53m subgroups, high-risk molecular features, and molecular taxonomy are insufficiently reported across these pivotal trials.

Modern molecular taxonomies indicate that *TP53* alterations occur in distinct biological contexts and that genetically defined DLBCL subtypes differ in gene-expression profiles, immune microenvironments, clinical outcomes, and potential therapeutic vulnerabilities.^43^^,^^66^^,^^67^ In the LymphGen framework, the A53 subtype is characterized by *TP53* mutations and/or deletions and is frequently associated with aneuploidy and genomic instability. In the Chapuy classification, the C2 cluster is enriched for biallelic TP53 inactivation and broad copy-number alterations. In a 2026 large-scale integrative molecular analysis of patients with ND DLBCL,^68^ 43.9% of TP53m cases have a VAF >50%, and higher VAF is associated with inferior PFS and OS. However, these predicted structural or functional alterations alone could not fully explain the survival heterogeneity among patients with TP53m DLBCL.

The future management of TP53m DLBCL requires a more precise and biomarker-stratified therapeutic strategy. Well-designed prospective trials, ideally randomized when feasible, are needed to evaluate novel combination regimens against established standards in molecularly defined TP53m populations. Future studies should mandate standardized *TP53* reporting. In addition, TP53m DLBCL should be prospectively stratified by COO subtype, DH/TH status, MYC/BCL2 double-expression status, and relevant co-mutations. Such biomarker-guided analyses should be conducted within contemporary molecular classification frameworks, including LymphGen and Chapuy subtypes, to clarify whether *TP53* alterations exert similar prognostic and predictive effects across different biological contexts. For cellular therapies, future studies should further report and analyze outcomes at the product and strategy levels. These efforts are essential to define which TP53m DLBCL subgroups are most likely to benefit from TT, cellular therapy, or rational combination strategies.

In conclusion, this SRMA highlights the limitations of current therapeutic strategies for TP53m DLBCL, while summarizing the available evidence on TT + Chemo in ND TP53m DLBCL and CAR-T-based approaches in R/R TP53m DLBCL. Although these strategies show measurable antitumor activity in selected cohorts, the evidence remains largely descriptive because of limited comparative data, heterogeneous molecular characterization, and variability in treatment regimens. Consequently, the optimal management of TP53m DLBCL should be further explored.

### Limitations of the study

This research has several limitations. Firstly, this analysis is primarily based on single-arm trials and retrospective observational studies. RCTs specifically designed for the TP53m DLBCL population are notably scarce. This inherent limitation might introduce selection bias and confounders, and the absence of direct control arms precludes robust determination of a superior therapeutic strategy. Furthermore, the sample size for specific treatment modalities is generally small, which might result in imprecise effect estimates and possibly overestimation of therapeutic benefits. Moreover, substantial statistical heterogeneity was observed across several key endpoints, which may be attributed to differences in study design, populations, targeted agents, duration of follow-up, and the timing of response assessment across studies. Secondly, CAR-T-based interventions also varied substantially across studies, including target antigen, CAR-T construct, product type, costimulatory domain, single- and dual-target strategy, combination with targeted agents, and consolidation approaches. These differences may influence clinical outcomes. However, subgroup analyses stratified by specific products or treatment strategies were not feasible due to limited sample sizes and incomplete reporting. Another important source of heterogeneity is likely the distribution of COO subtypes in included studies. The prognostic weight and therapeutic responses of TP53 mutations can be profoundly modulated by the COO-based context.^9^^,^^69^ Owing to inconsistent reporting in the original studies, this research cannot perform COO-based subgroup analyses. This also represents a major limitation of the current study, hindering deeper insight into therapeutic efficacy. Lastly, although this study focused on TP53m DLBCL, mutation-level reporting was heterogeneous across the included studies. Therefore, we were unable to perform robust subgroup analyses according to mutation class, disruptive and non-disruptive mutations, VAF, clonal status, or allelic status.

## Resource availability

### Lead contact

Further information and requests for resources should be directed to and will be fulfilled by the lead contact, Ningning Shan (snning@126.com).

### Materials availability

This study did not generate new unique reagents.

### Data and code availability

• This study is a systematic review and meta-analysis. The data analyzed in this study were extracted from previously published studies and publicly available resources.
• No custom code was generated; all analyses were performed using publicly available software as detailed in the key resources table.
• Any additional information required to reanalyze the data reported in this paper is available from the lead contact upon request.

## Acknowledgments

This work was supported by Taishan Youth Scholar Foundation of Shandong Province (tsqn201812140), 10.13039/501100001809National Natural Science Foundation of China of China (no. 81570104).

## Author contributions

X.Z.: data curation, writing – original draft preparation; Y.J.: conceptualization, methodology, software; A.L.: visualization, investigation; D.K.: supervision; P.Z.: software, validation; N.S.: writing – reviewing and editing, funding acquisition, and all authors commented on previous versions of the manuscript. All authors read and approved the final manuscript.

## Declaration of interests

The authors declare no competing interests.

## Declaration of generative AI and AI-assisted technologies in the writing process

Not applicable.

## STAR★Methods

### Key resources table

**Table:** 

REAGENT or RESOURCE	SOURCE	IDENTIFIER
**Deposited data**		

PubMed	U.S. National Library of Medicine	https://pubmed.ncbi.nlm.nih.gov/
EMBASE	Elsevier	https://www.embase.com/
Web of Science	Clarivate	https://www.webofscience.com/wos/author/search
Cochrane Library	Wiley	https://www.cochranelibrary.com/search?cookiesEnabled
International prospective register of systematic reviews	PROSPERO	https://www.crd.york.ac.uk/PROSPERO/

**Software and algorithms**		

R statistical software (Version 4.4.1)	CRAN	https://www.r-project.org/

### Method details

This SRMA was implemented based upon the Preferred Reporting Items for Systematic Reviews and Meta-Analyses (PRISMA) guidelines^70^ (Supplementary Material 1). The study protocol was prospectively registered with the International Prospective Register of Systematic Reviews (PROSPERO# CRD420251174299).

#### Search strategy

Two reviewers (DK and PZ) searched PubMed, Embase, Web of Science, and the Cochrane Library up to September 3, 2025, without language restrictions. Before the final formal search, the search strategies proposed by both reviewers were integrated, jointly checked and verified to ensure reproducibility and consistency, and the final search was then conducted using the agreed strategy. Detailed search strategies are demonstrated in Table S3. Moreover, relevant review articles and their reference lists were screened to ascertain other potentially eligible studies.^6^^,^^71^^,^^72^

#### Selection criteria

Inclusion criteria were outlined below: (i) studies evaluating remission-oriented therapeutic strategies among individuals with ND or R/R TP53m DLBCL. TP53m DLBCL was defined as the presence of at least one somatic *TP53* mutation detected by sequencing-based methods. The target population was TP53m DLBCL, primarily DLBCL not otherwise specified (DLBCL-NOS). However, because some original studies used broader diagnostic terms such as large B-cell lymphoma or aggressive B-cell lymphoma, cohorts could include high-grade B-cell lymphoma, double-hit/triple-hit lymphoma, transformed DLBCL, or other related large B-cell lymphoma subtypes. When subtype-specific data were available, DLBCL or TP53m DLBCL data were extracted preferentially. Studies were retained when TP53m DLBCL-specific outcomes were reported or when the included cohort was considered clinically consistent with DLBCL/large B-cell lymphoma as defined by the original study. Eligible *TP53* variants included non-synonymous mutations (missense, nonsense, frameshift, and splice-site) as reported in the original studies. *TP53* deletion (e.g., 17p loss) without sequencing-confirmed mutation was not classified as TP53m and was excluded from the analysis; (ii) studies reporting at least one primary efficacy endpoint, including CR, ORR, OS, or PFS; (iii) eligible study designs encompassed RCTs, single-arm trials, prospective observational studies, and retrospective studies. For studies involving mixed populations, solely those providing data on subgroups of TP53m DLBCL were included.

Exclusion criteria were outlined below: (i) animal or cell experiments, case reports, study protocols, reviews, letters, editorials, and conference abstracts; (ii) studies with missing data or serious data errors; (iii) duplicate publications; (iv) studies with unavailable complete texts.

Two reviewers (XZ and YJ) independently screened studies for eligibility based upon the predefined criteria. Titles and abstracts of all retrieved studies were first screened to exclude obviously irrelevant studies. Complete texts of the remaining articles were then attained and subjected to a second round of assessment by both reviewers. Any divergences were resolved through discussions or by consulting a third researcher (NS).

#### Data extraction and quality assessment

Two reviewers (YJ and AL) independently extracted data from every included study utilizing a specifically designed electronic spreadsheet. Extracted information comprised study characteristics, features of patients, details of treatment, methods of TP53 mutation analysis, study outcomes, and methodological quality.

Two reviewers (XZ and PZ) independently assessed the methodological quality and risk of bias of the included studies. RCTs were evaluated using the revised Cochrane risk-of-bias tool for randomized trials (RoB 2),^73^ which assesses bias arising from the randomization process, deviations from intended interventions, missing outcome data, measurement of outcomes, and selection of the reported result. Non-randomized studies were assessed using the ROBINS-I.^74^ This tool covered bias due to confounding, selection of participants, classification of interventions, deviations from intended interventions, missing data, measurement of outcomes, and selection of the reported result. Each study was judged according to the corresponding tool-specific domains. Meanwhile, an overall risk of bias was assessed. In cases of missing or ambiguous data, the original authors were contacted for clarification. Any discrepancies were resolved through discussion or consultation with a third reviewer (NS).

### Quantification and statistical analysis

The pre-specified therapeutic strategies evaluated in this research comprised six categories: TT, Chemo, combined TT and Chemo (TT + Chemo), Allo-HSCT, CAR-T, and bispecific antibody therapy. TT was defined as the use of non-cytotoxic agents directed against specific molecular pathways or immune targets, including Bruton tyrosine kinase inhibitors, BCL-2 inhibitors, immunomodulatory agents, anti-CD38 monoclonal antibodies, immune checkpoint inhibitors, and other molecularly targeted agents, as reported in the original studies. However, after the review of the eligible studies, no trials involving allo-HSCT or bispecific antibodies were identified.

The primary endpoints encompassed CR, ORR (ascertained as partial remission [PR] or CR based upon the Lugano classification),^75^ OS (defined as the time from initiation of treatment to all-cause mortality [ACM]), and PFS (defined as the time from initiation of treatment to disease progression or ACM). Regarding CR and ORR, this research extracted the number of events and the total sample size for every therapeutic regimen. Regarding OS and PFS, this research extracted the survival rates at all reported time points for therapeutic regimen, along with the median OS and PFS and their 95% confidence intervals (CI). If an included study reported more than one eligible arm, we extracted outcome data separately for each eligible arm and treated each arm as an independent cohort in the corresponding treatment category. When two arms shared overlapping patients, only the arm aligning with the predefined treatment category was retained to prevent double counting.

Statistical analyses were implemented utilizing R (v 4.4.1). We calculated aggregated CR rate, ORR, median OS, 1-, 2-, 3-, and 5-year OS rates, median PFS, and 1-, 2-, 3-, and 5-year PFS rates.

The original rates from each study were transformed utilizing the Freeman–Tukey double arcsine method and then pooled utilizing the meta package.^76^ The median OS and PFS and their 95% CIs were synthesized using the meta-mean package.^77^^,^^78^ Between-study heterogeneity was estimated leveraging the chi-square test, with *p* < 0.10 signifying significant heterogeneity. The degree of heterogeneity was quantified leveraging the *I*^2^ statistic (*I*^2^ = 0–25%: low heterogeneity; 25–50%: moderate heterogeneity; 50–75%: substantial heterogeneity; 75–100%: considerable heterogeneity). Random-effects models were applied to all pooled estimates to account for between-study variability.

Sensitivity analyses were implemented utilizing a leave-one-out approach. When the number of included studies exceeded 10, publication bias was estimated utilizing funnel plots. Egger’s test was applied for statistical assessment (*p* < 0.05 signified statistical significance).^79^ Subgroup analyses were implemented according to study design (retrospective or prospective), region (Asia, North America, Europe, or Oceania), sample size (≤10 or >10), disease status (ND or R/R), methods for detecting TP53 mutations (Sanger sequencing, next-generation sequencing, or unknown), and therapeutic methods (TT, Chemo, TT + Chemo, CAR-T). Distinctions between subgroups were appraised by meta-regression analysis, with the level of significance set at *p* < 0.05.

## Supplemental information

Document S1. Figures S1–S37, Table S2, and Data S1

Table S1. Single Extraction Table for TP53-Related Information in TP53-Mutated DLBCL Studies

Table S3. Detailed search strategies
