# Supplementary material for: Therapeutic outcomes of TP53-mutated diffuse large B cell lymphoma: A systematic review and meta-analysis
Source: iScience. 2026 Aug 6;29(8):117049. doi: 10.1016/j.isci.2026.117049 (PMC13475677; doi:10.1016/j.isci.2026.117049)
Supplement: Supplementary file 1 [file mmc1.pdf]

iScience, Volume *29*

Supplemental information

Therapeutic outcomes of TP53-mutated diffuse large B cell lymphoma: A systematic review and meta-analysis

Xin Zhou, Yang Jiang, Ai Li, Dexiao Kong, Peipei Zhang, and Ningning Shan

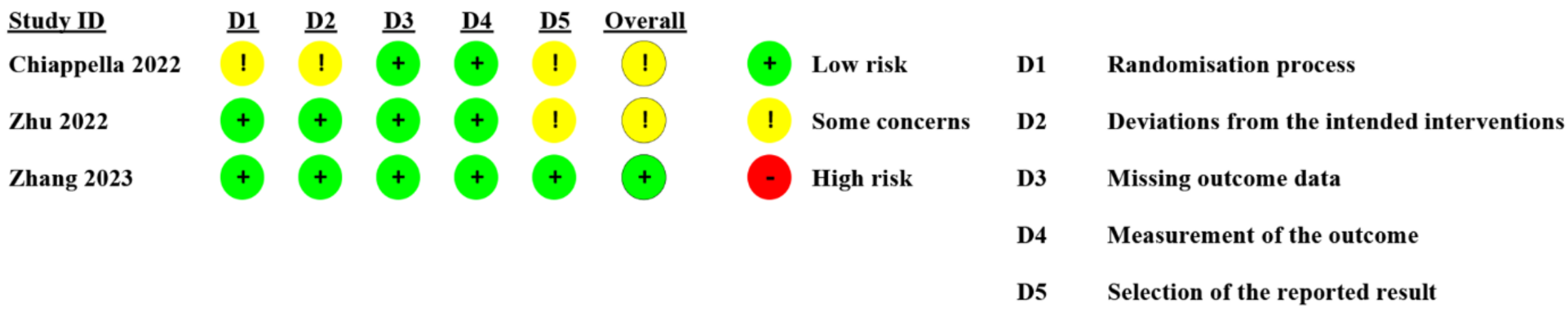

Fig. S1 Quality assessment results of each included randomized study.

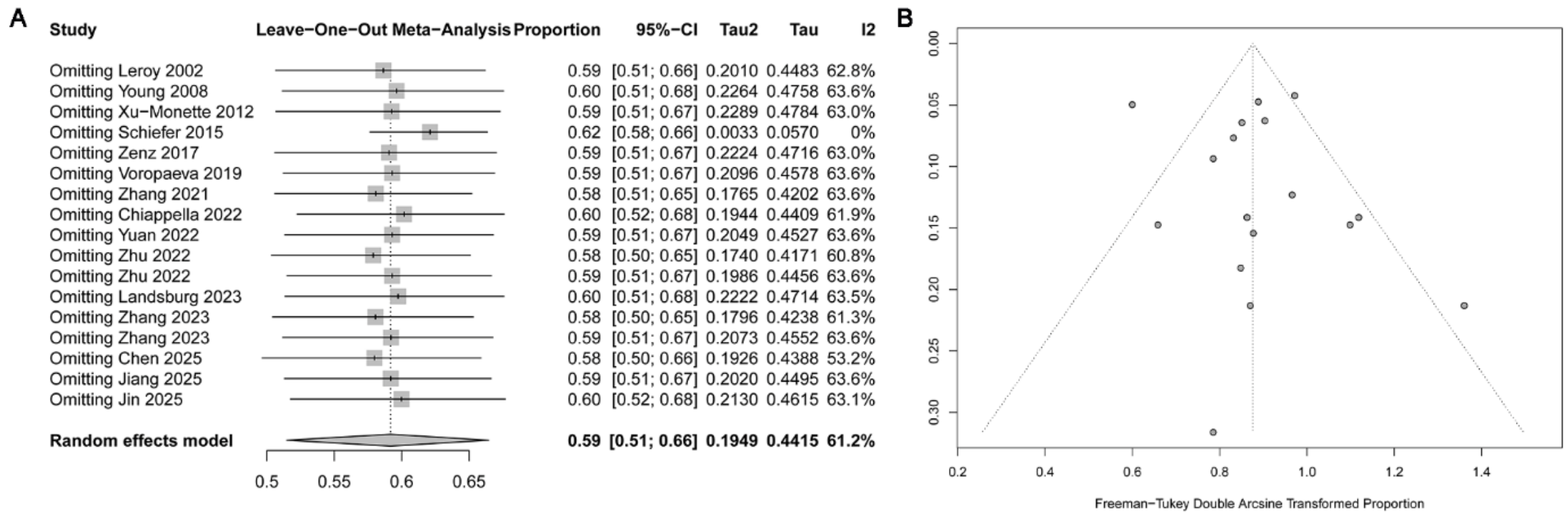

Fig. S2 Sensitivity analysis and publication bias assessment of CR rate in newly diagnosed TP53-mutated DLBCL. (A) Sensitivity analysis of the CR rate by removing one study at a time. (B) Funnel plot of the CR rate. Each dot represents an individual study included in the meta-analysis.

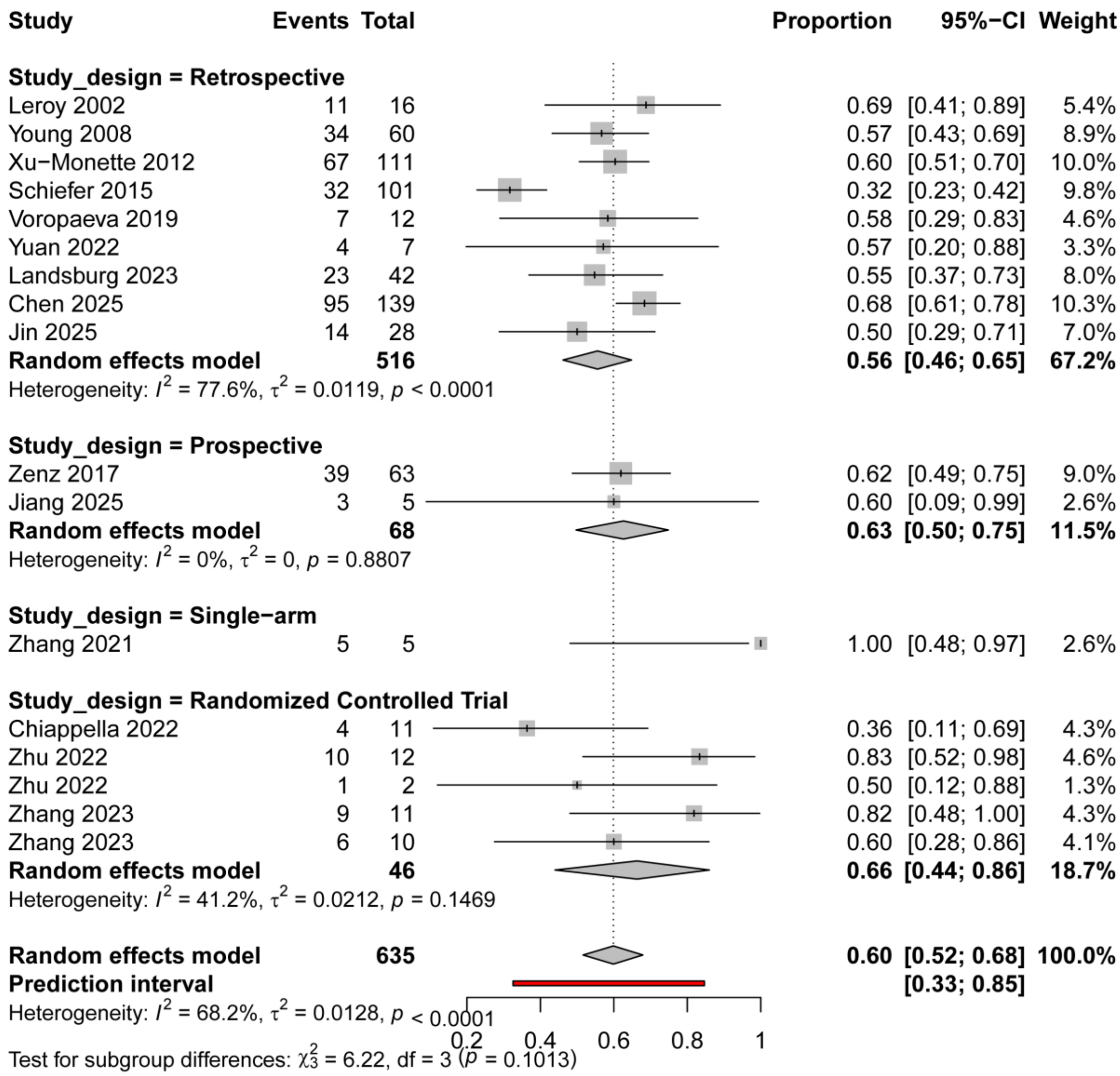

Fig. S3 Subgroup analysis of CR rate in nd DLBCL according to current remission strategies, stratified by study design.

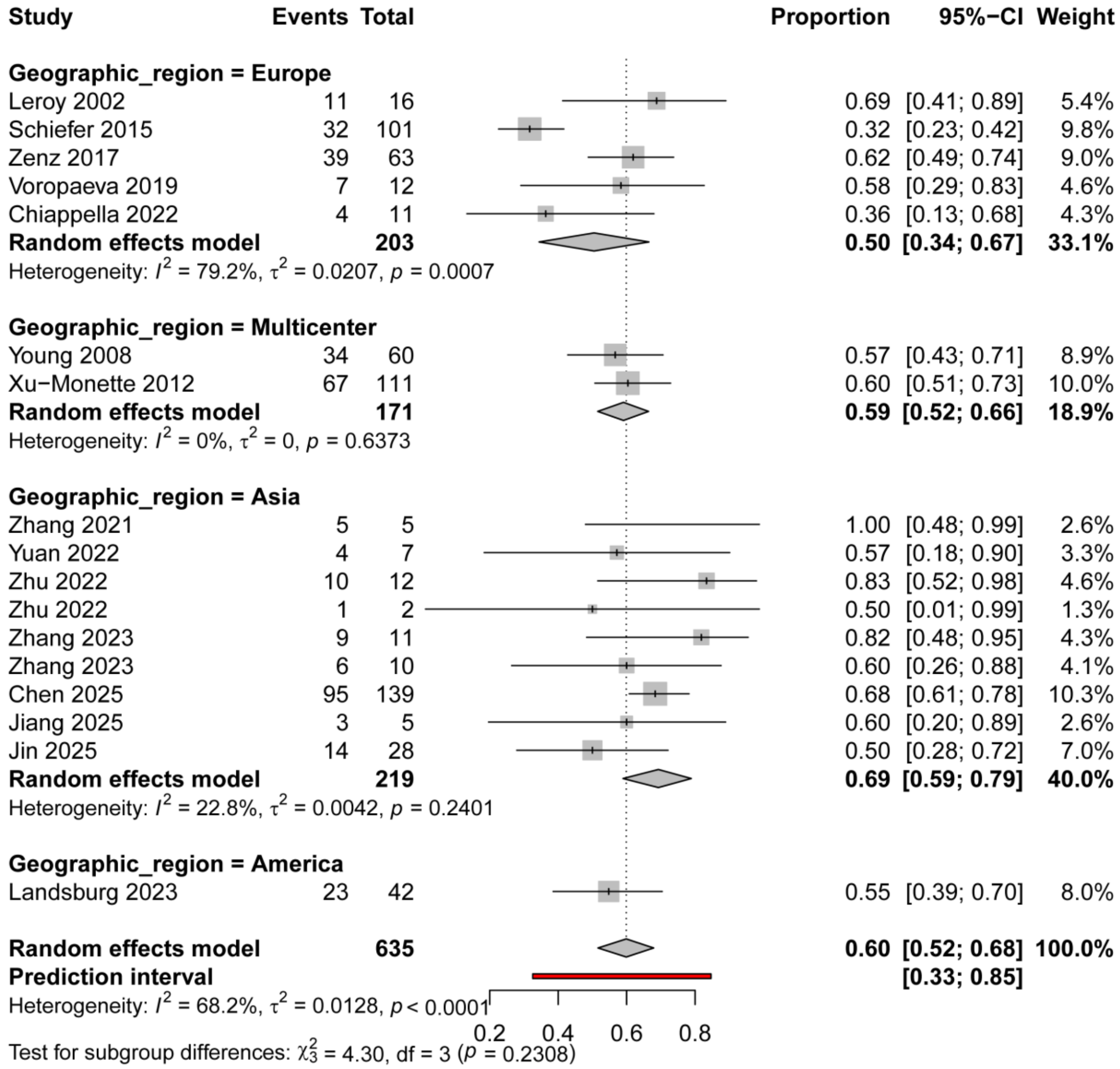

Fig. S4 Subgroup analysis of CR rate in nd DLBCL according to current remission strategies, stratified by region.

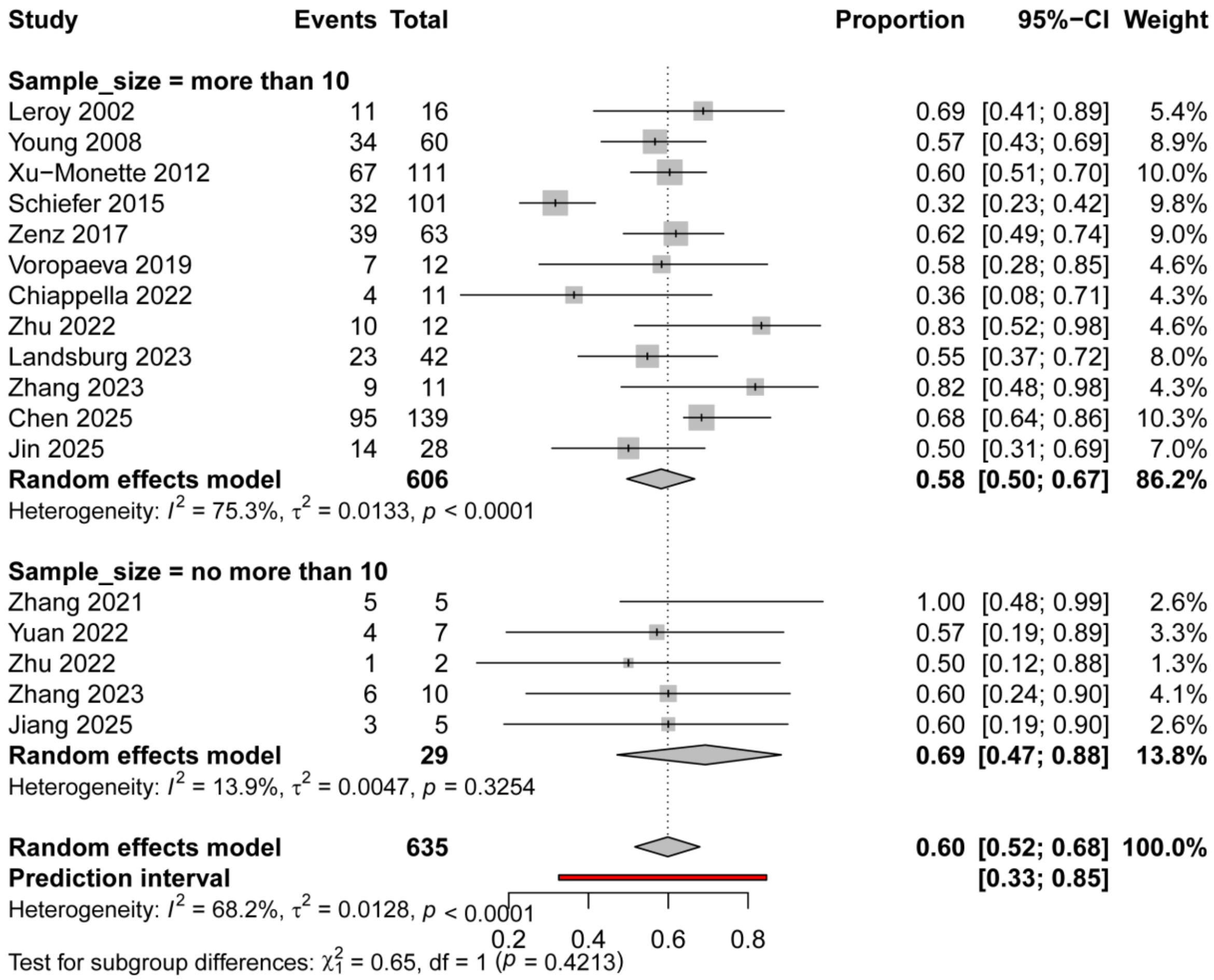

Fig. S5 Subgroup analysis of CR rate in nd DLBCL according to current remission strategies, stratified by sample size.

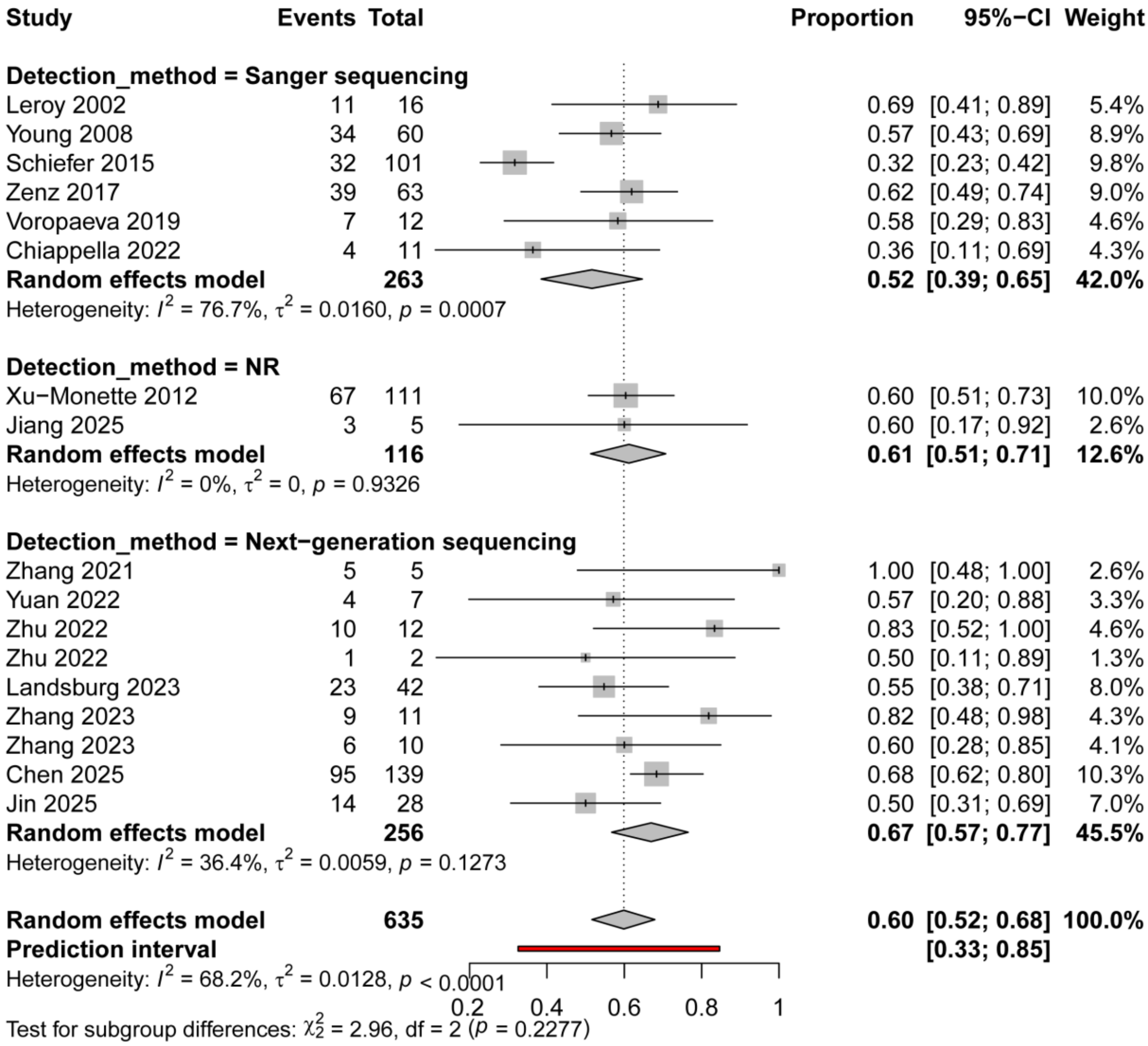

Fig. S6 Subgroup analysis of CR rate in nd DLBCL according to current remission strategies, stratified by detection method.

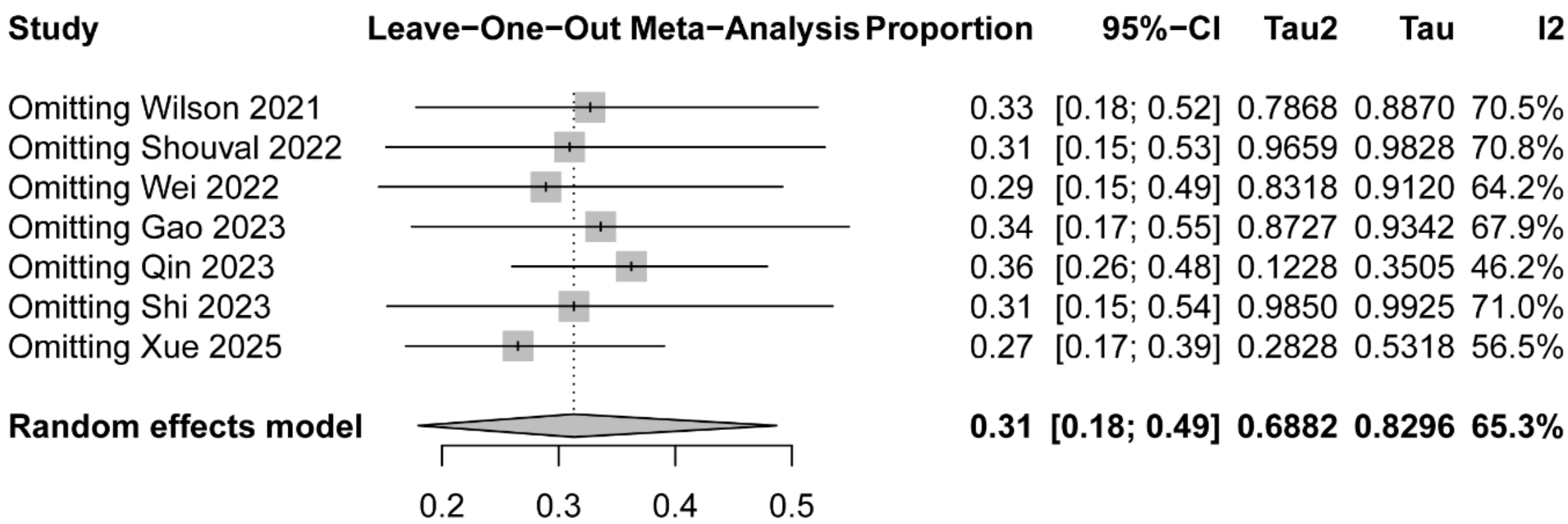

Fig. S7 Sensitivity analysis of CR rate in rr DLBCL by removing one study at a time.

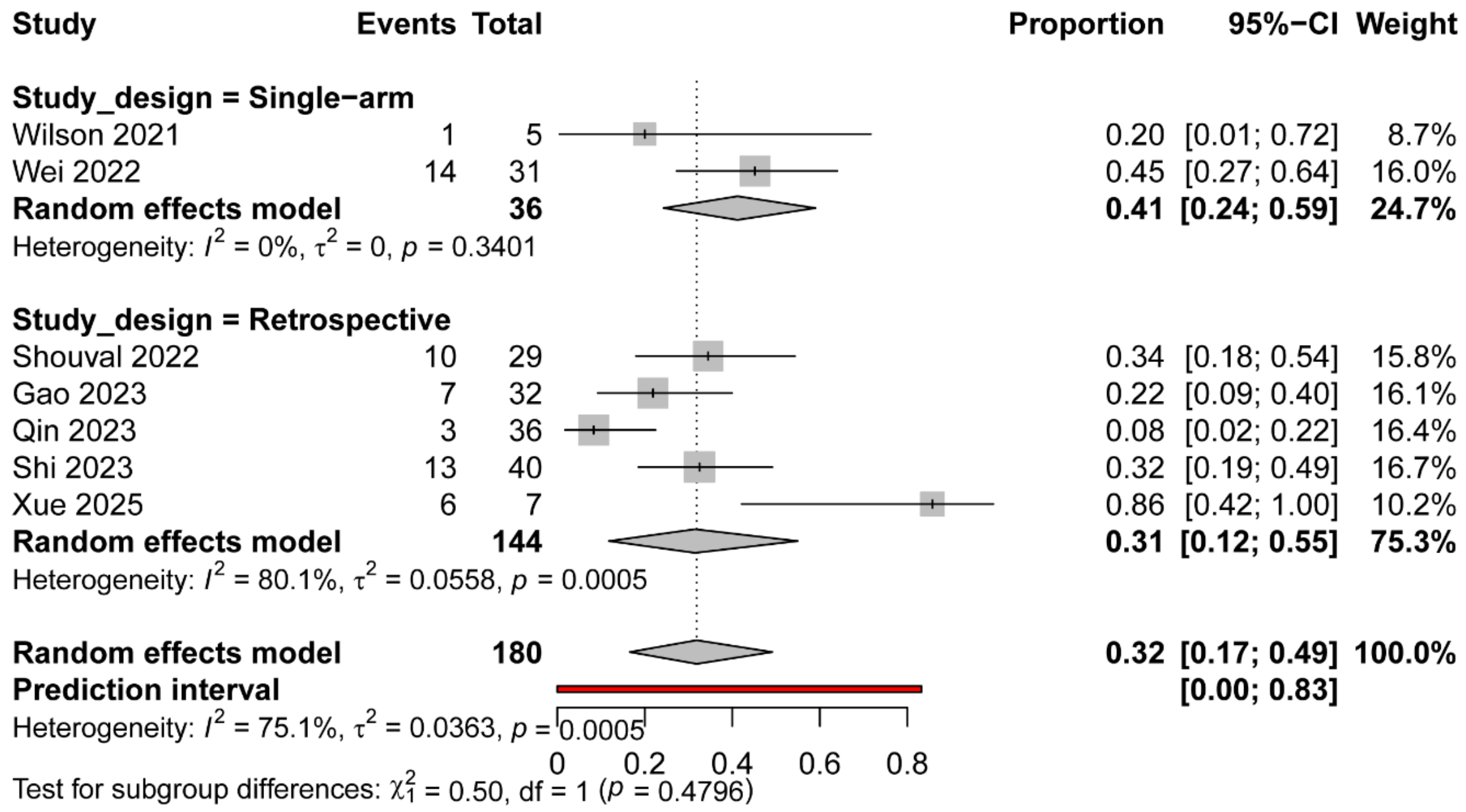

Fig. S8 Subgroup analysis of CR rate in rr DLBCL according to current remission strategies, stratified by study design.

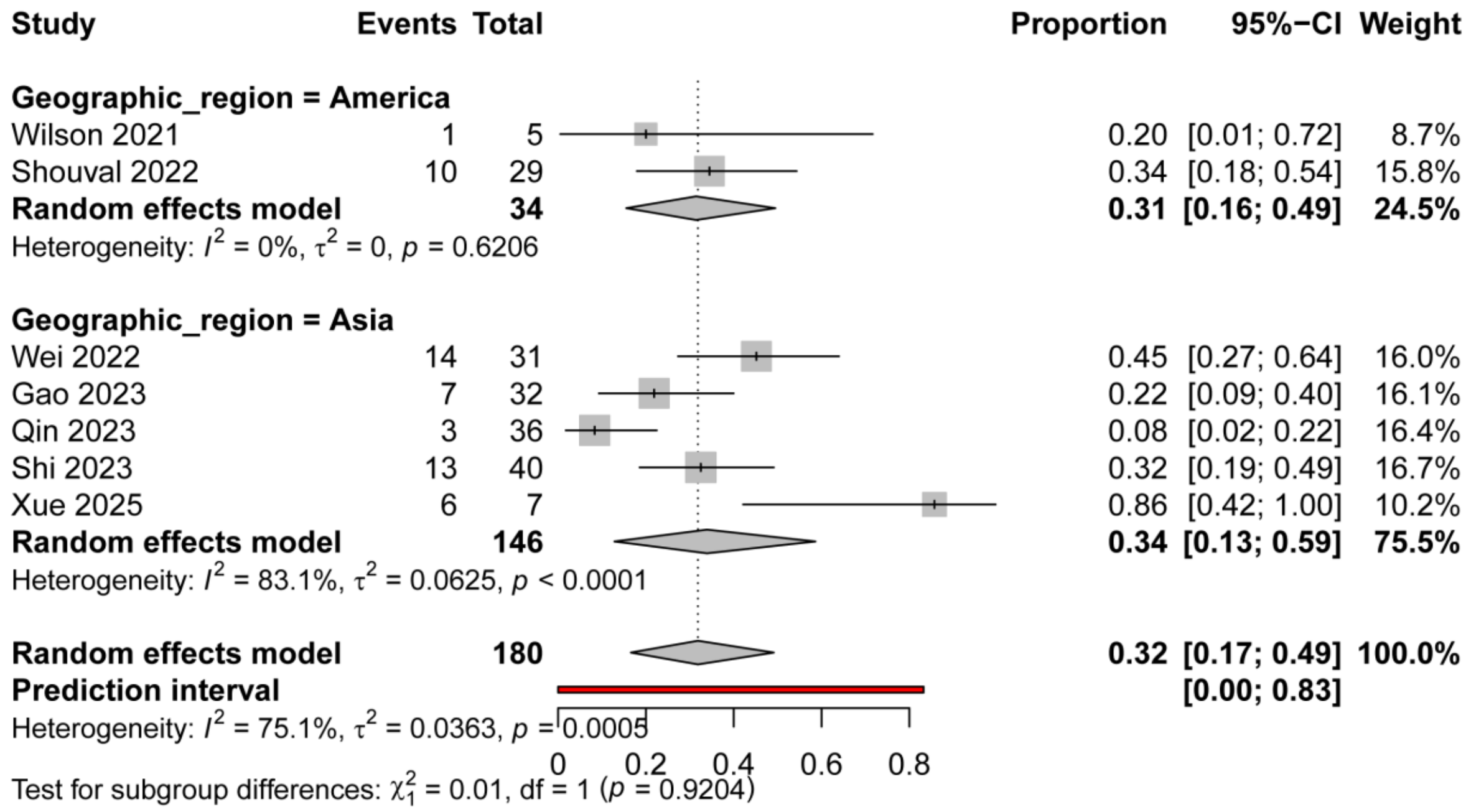

Fig. S9 Subgroup analysis of CR rate in rr DLBCL according to current remission strategies, stratified by region.

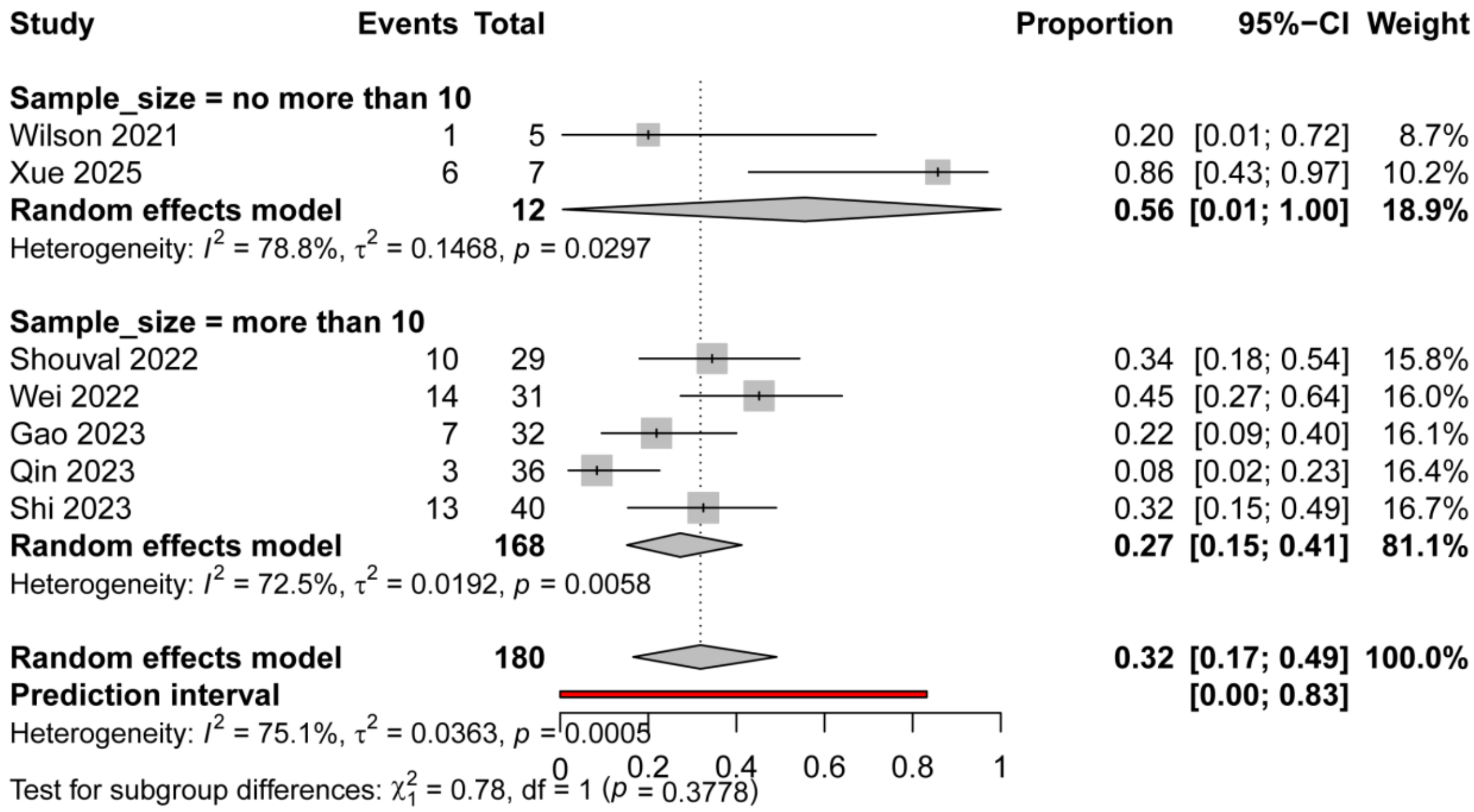

Fig. S10 Subgroup analysis of CR rate in rr DLBCL according to current remission strategies, stratified by sample size.

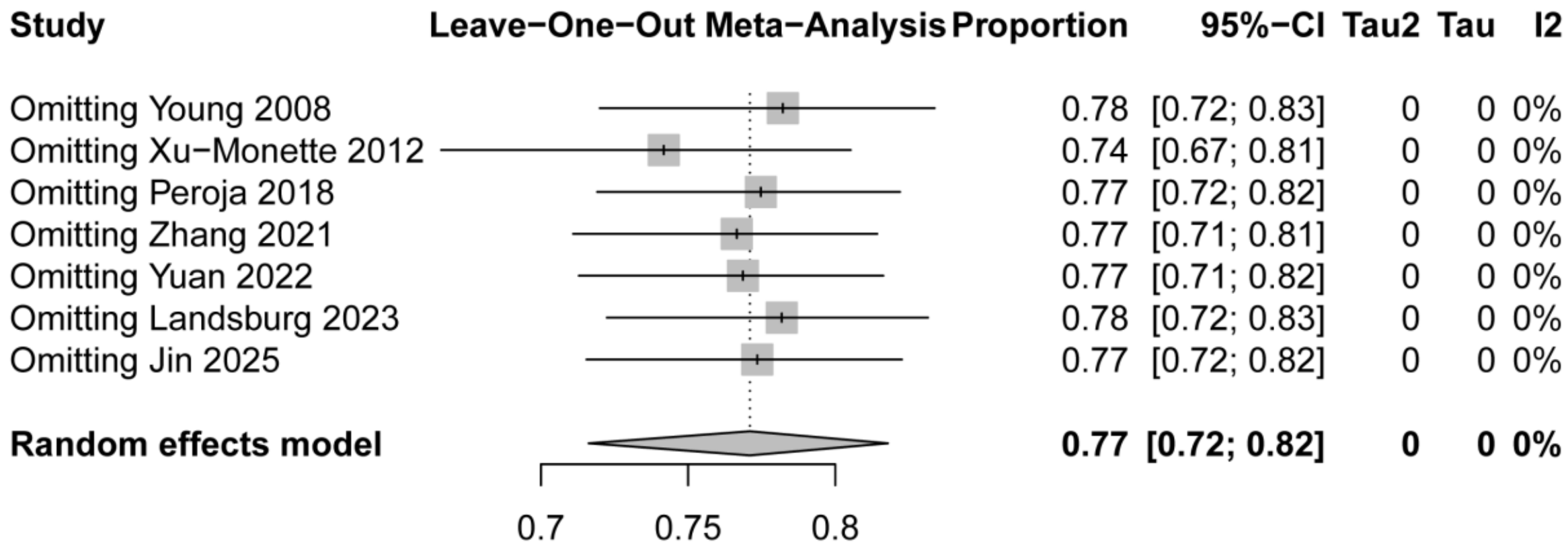

Fig. S11 Sensitivity analysis of ORR in nd DLBCL by removing one study at a time.

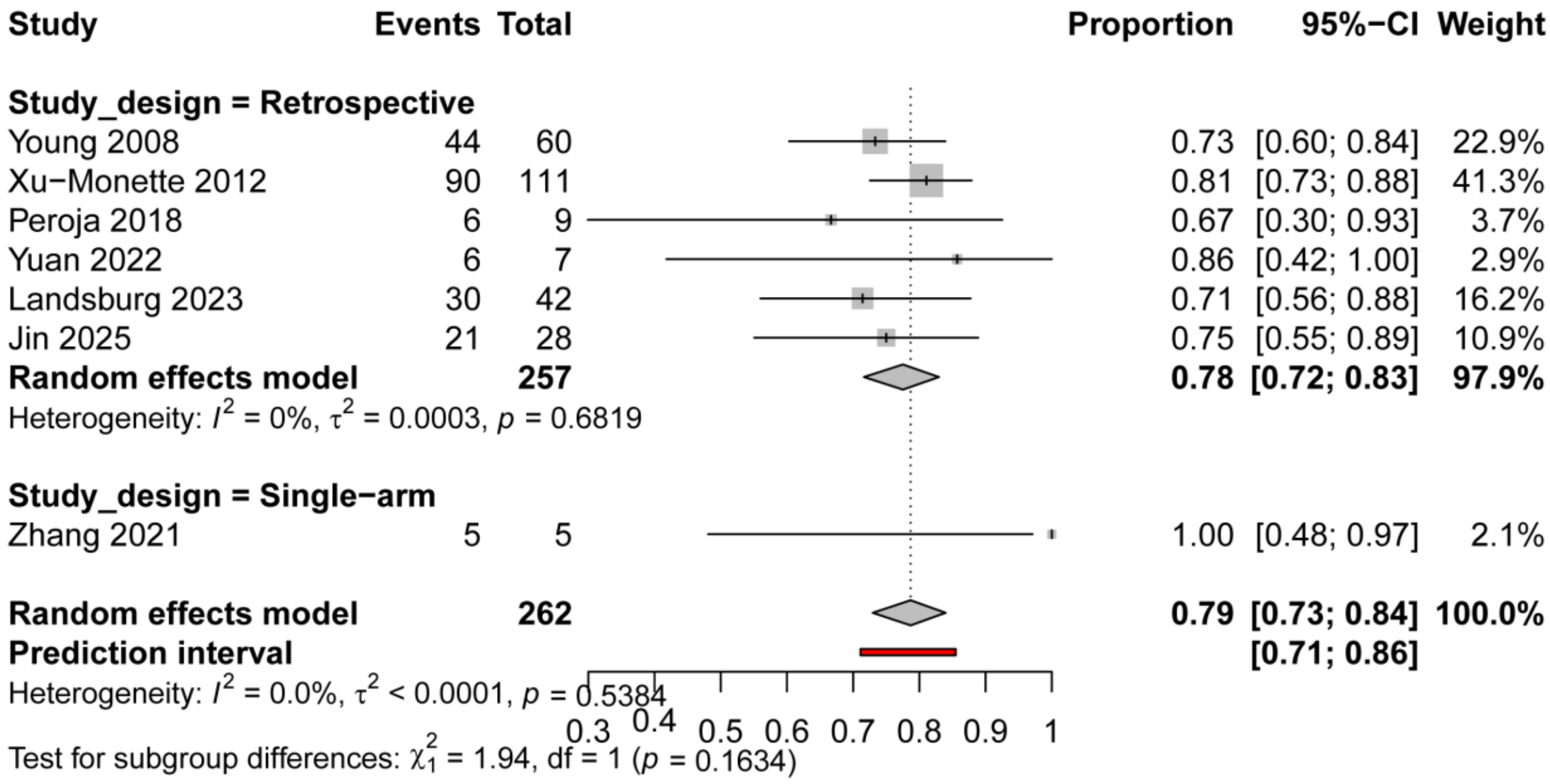

Fig. S12 Subgroup analysis of ORR in nd DLBCL according to current remission strategies, stratified by study design.

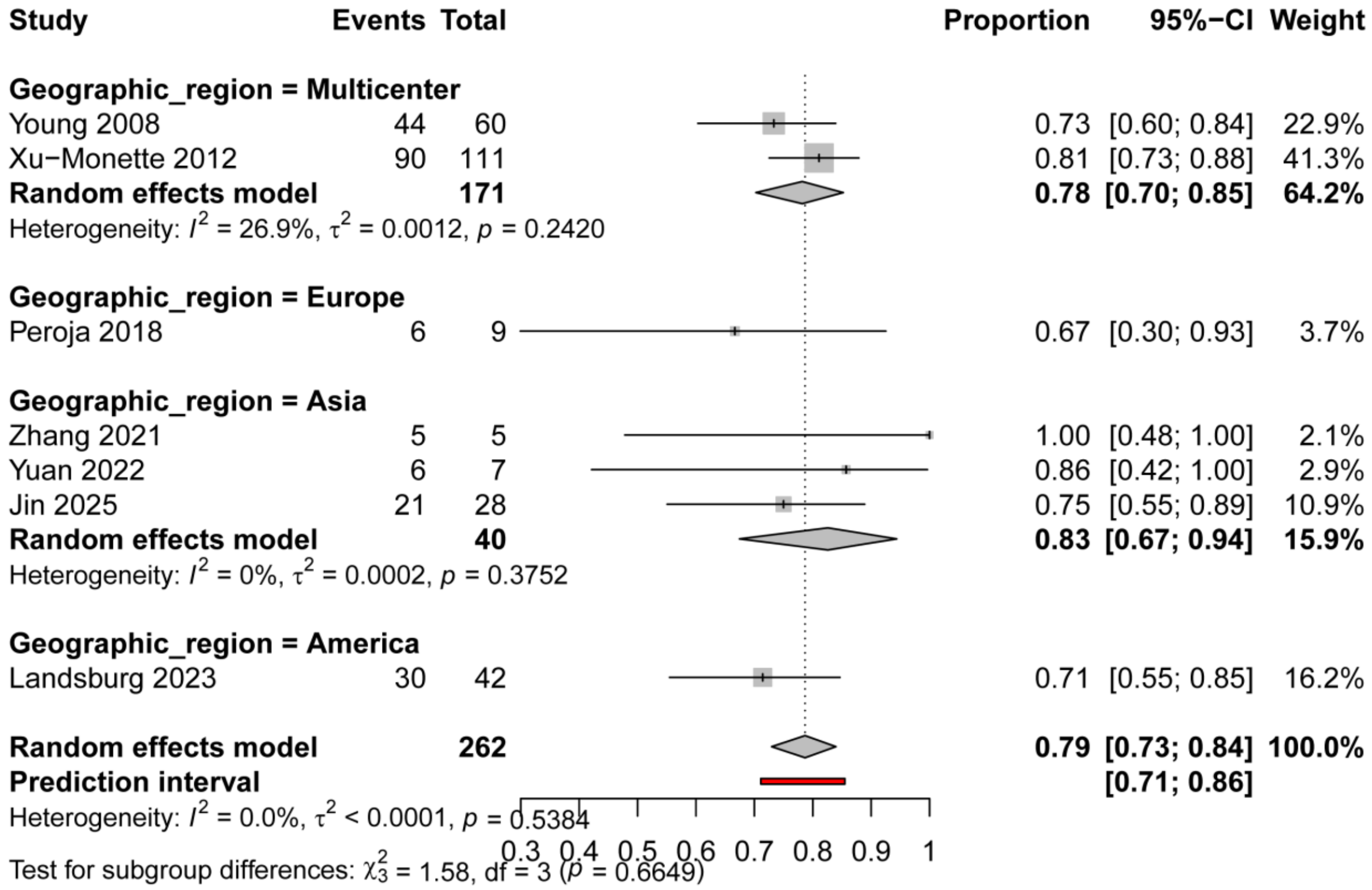

Fig. S13 Subgroup analysis of ORR in nd DLBCL according to current remission strategies, stratified by region.

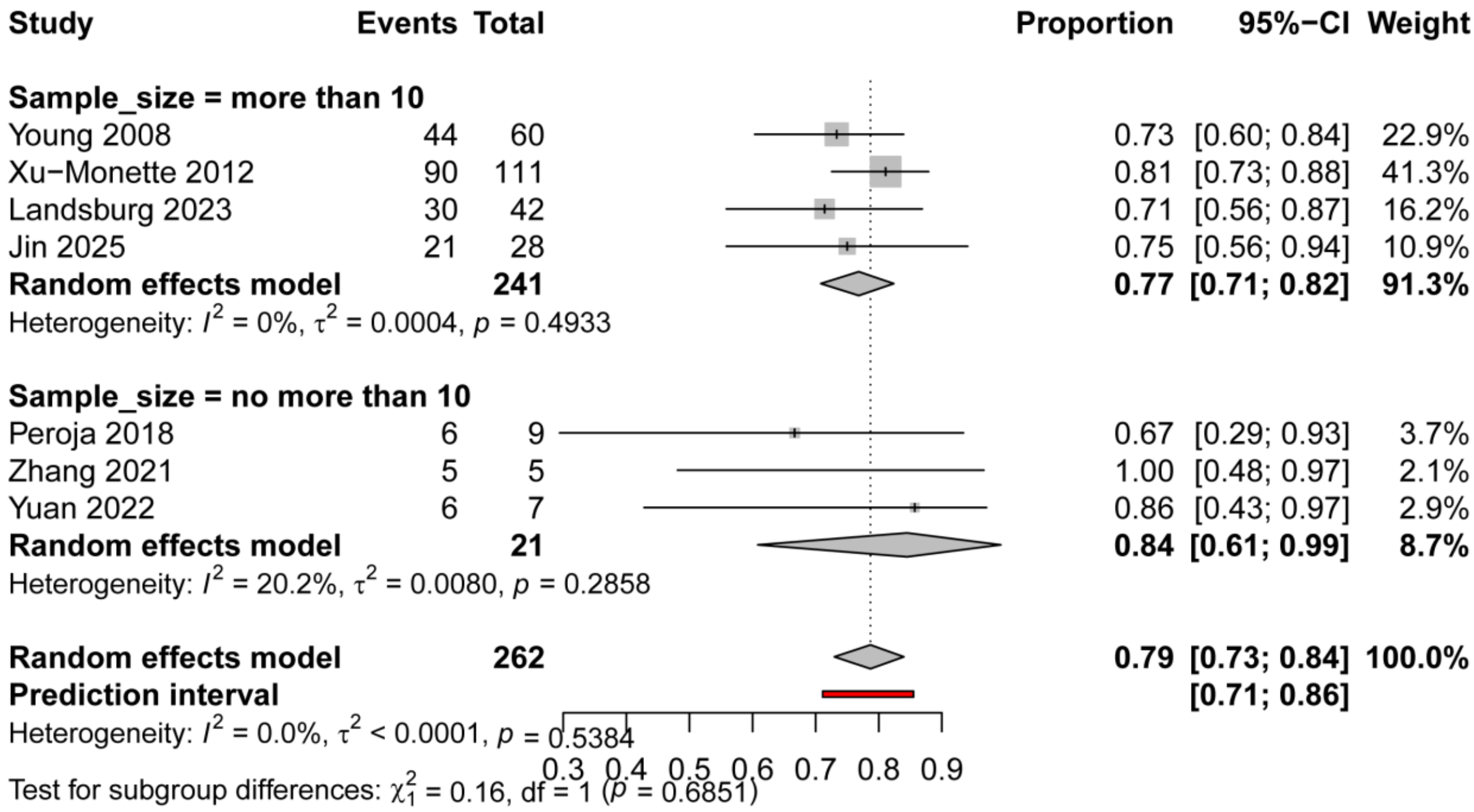

Fig. S14 Subgroup analysis of ORR in nd DLBCL according to current remission strategies, stratified by sample size.

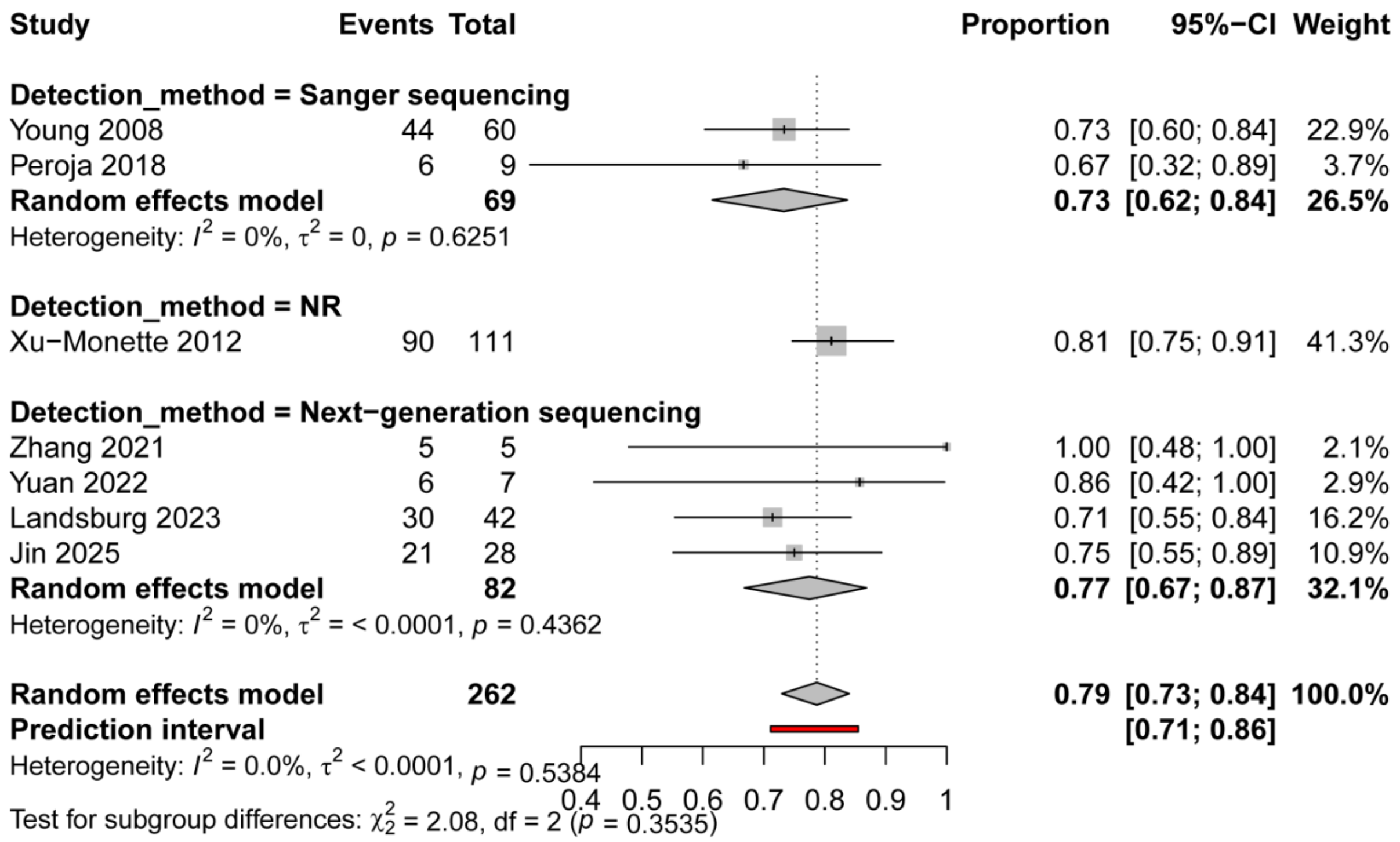

Fig. S15 Subgroup analysis of ORR in nd DLBCL according to current remission strategies, stratified by detection method.

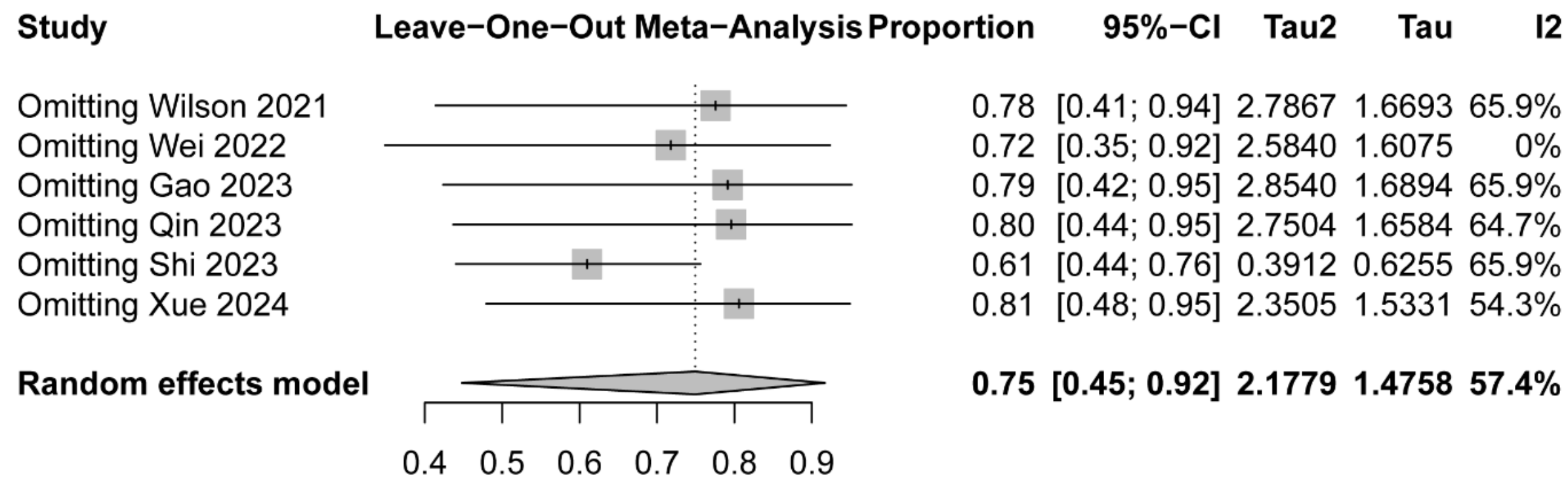

Fig. S16 Sensitivity analysis of ORR in rr DLBCL by removing one study at a time.

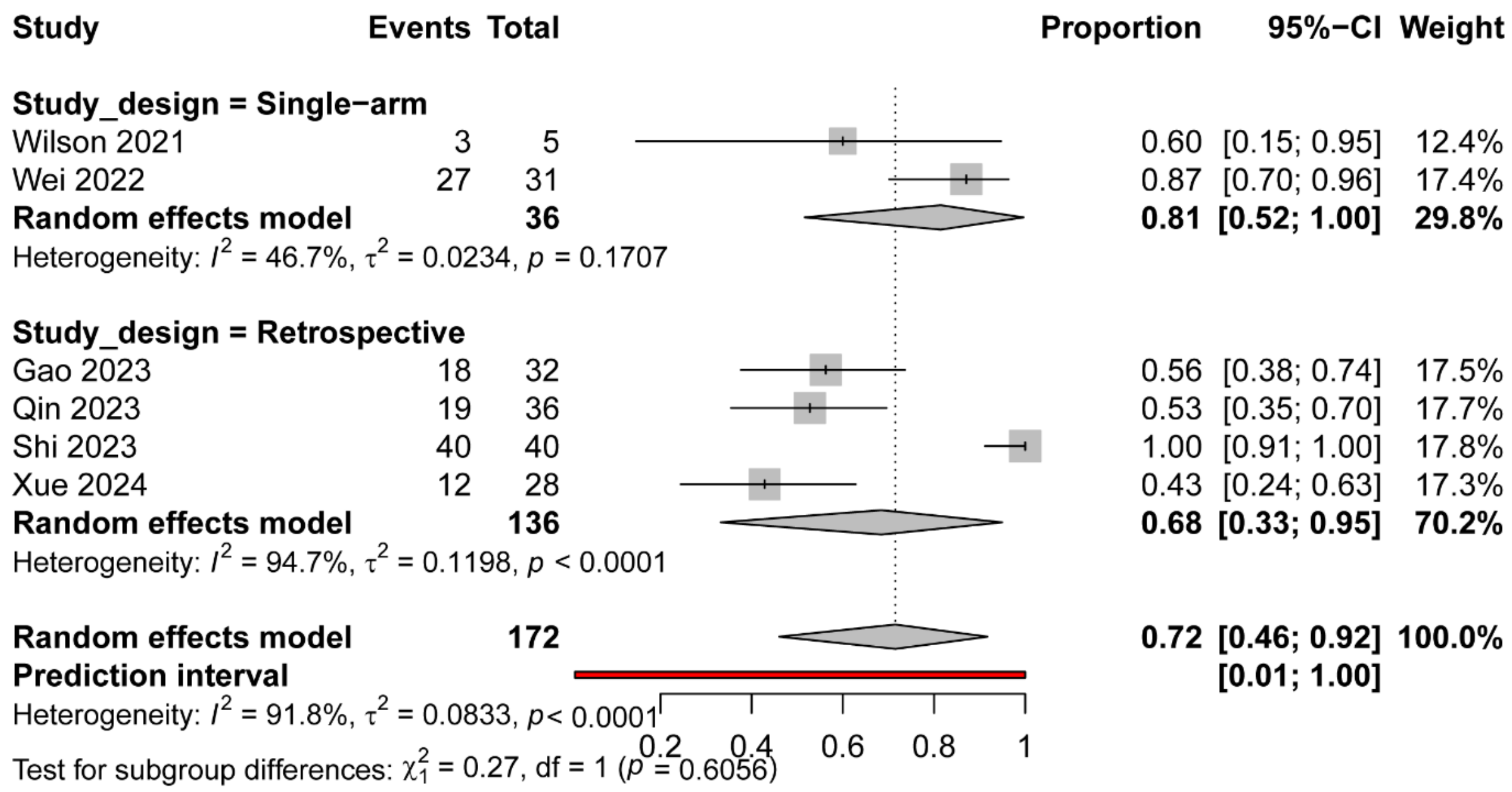

Fig. S17 Subgroup analysis of ORR in rr DLBCL according to current remission strategies, stratified by study design.

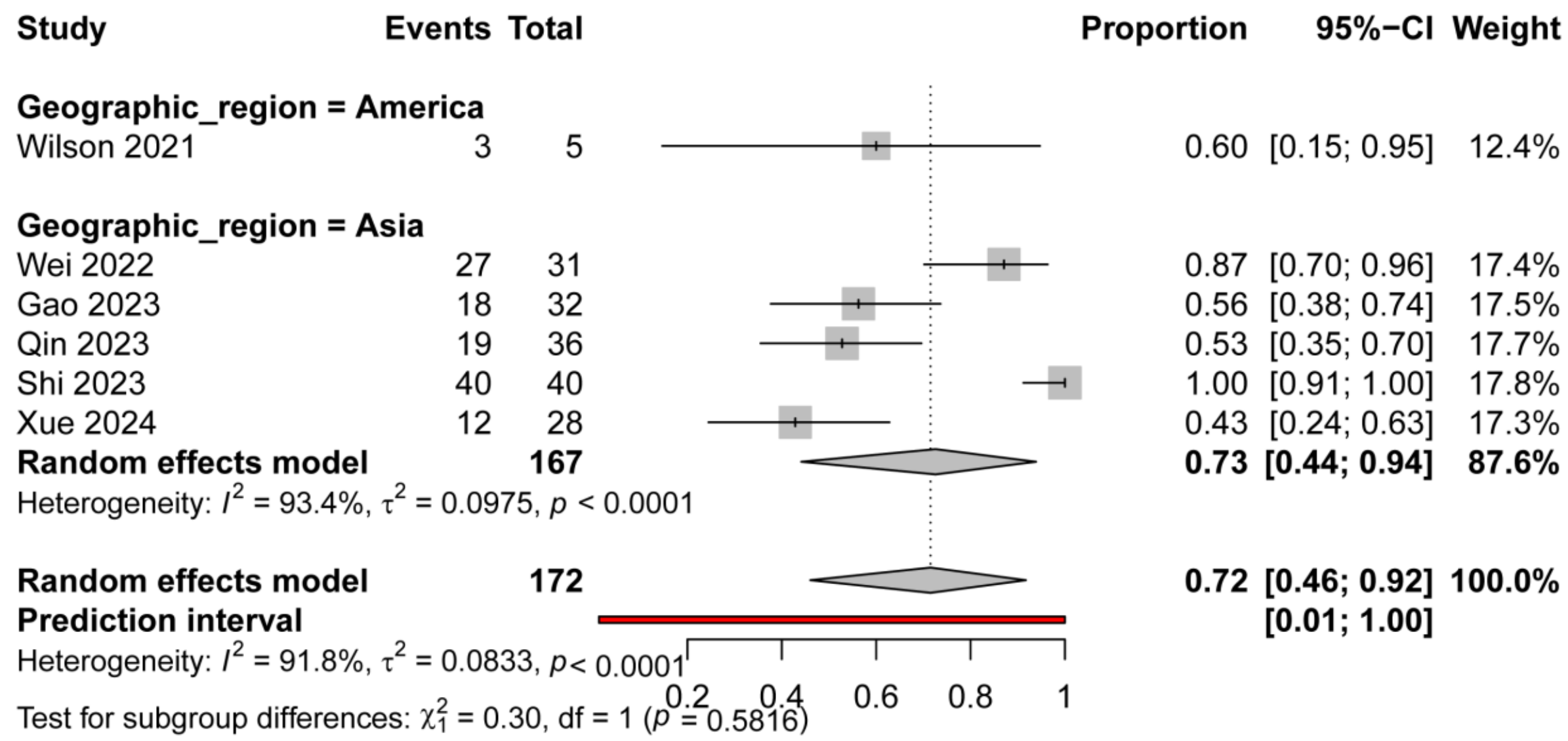

Fig. S18 Subgroup analysis of ORR in rr DLBCL according to current remission strategies, stratified by region.

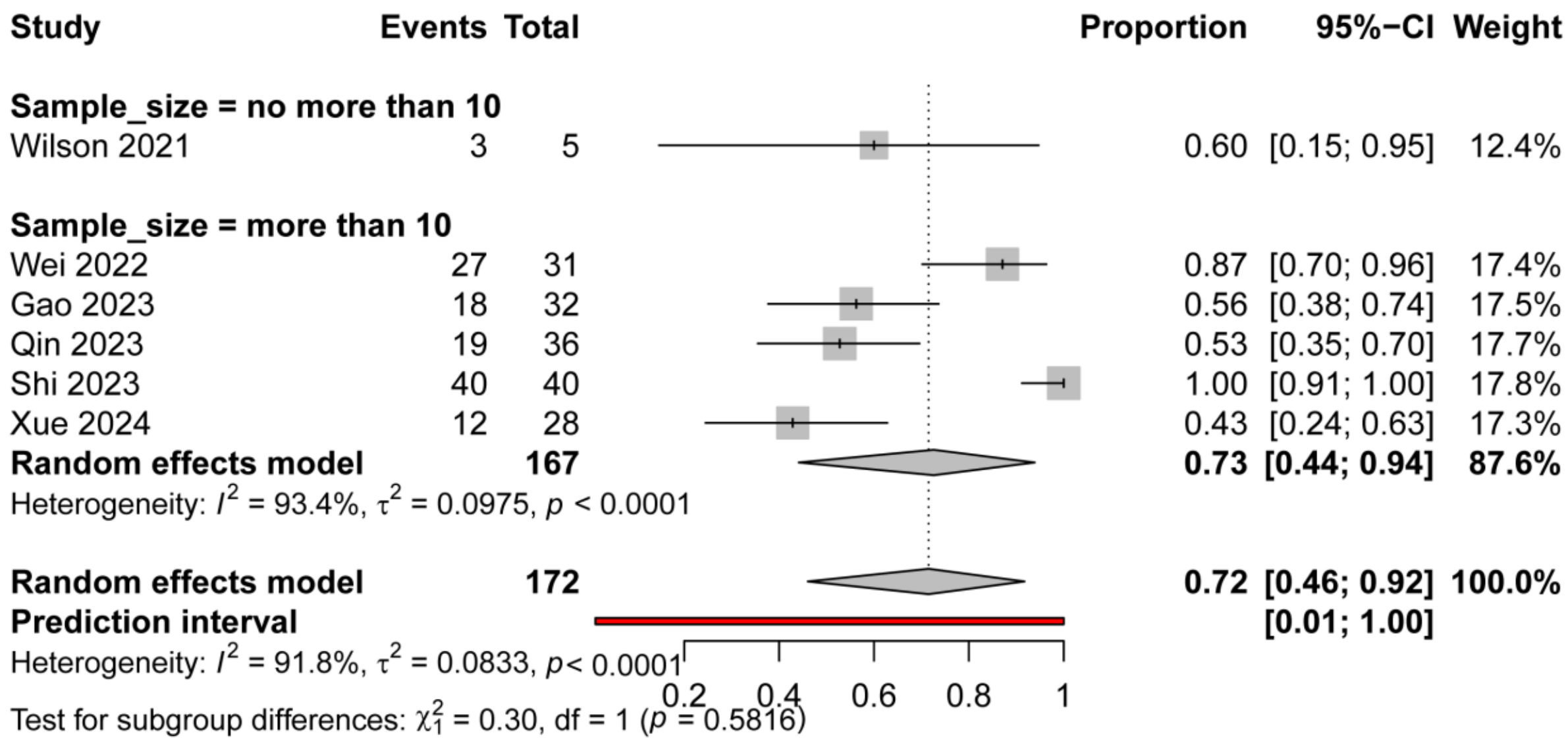

Fig. S19 Subgroup analysis of ORR in rr DLBCL according to current remission strategies, stratified by sample size.

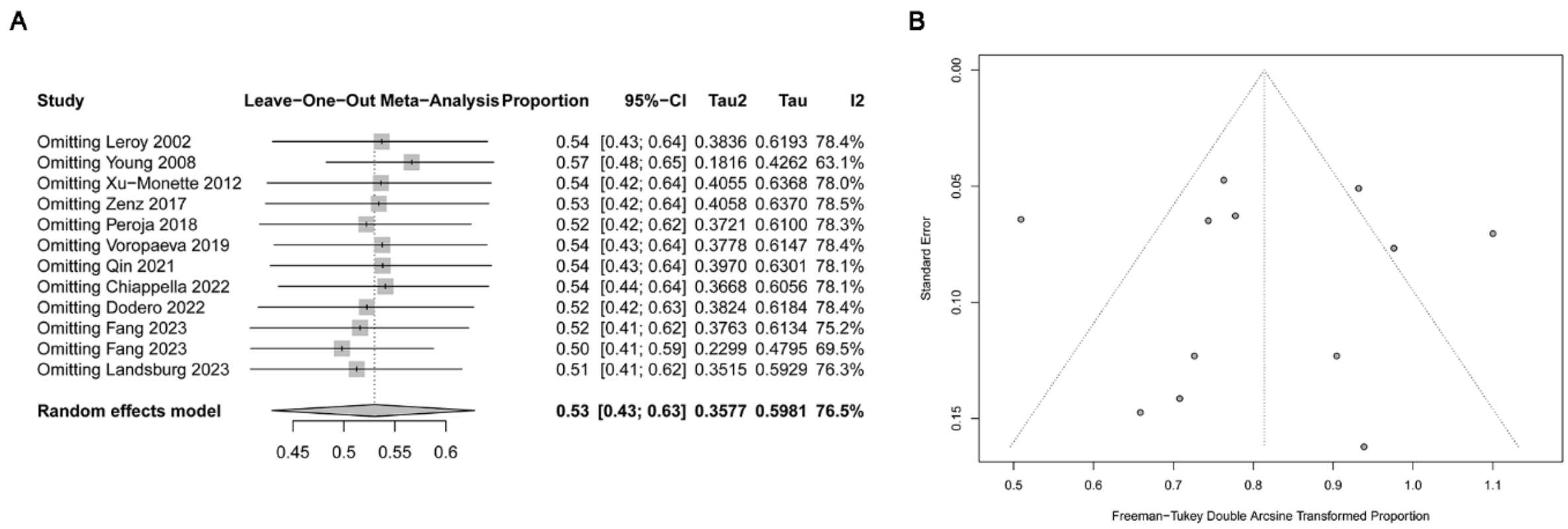

Fig. S20 Sensitivity analysis and publication bias assessment for the pooled overall survival rate in newly diagnosed TP53-mutated DLBCL. (A) Sensitivity analysis of the pooled OS rate by removing one study at a time. (B) Funnel plot assessing publication bias for the pooled OS rate. Each dot represents an individual study.

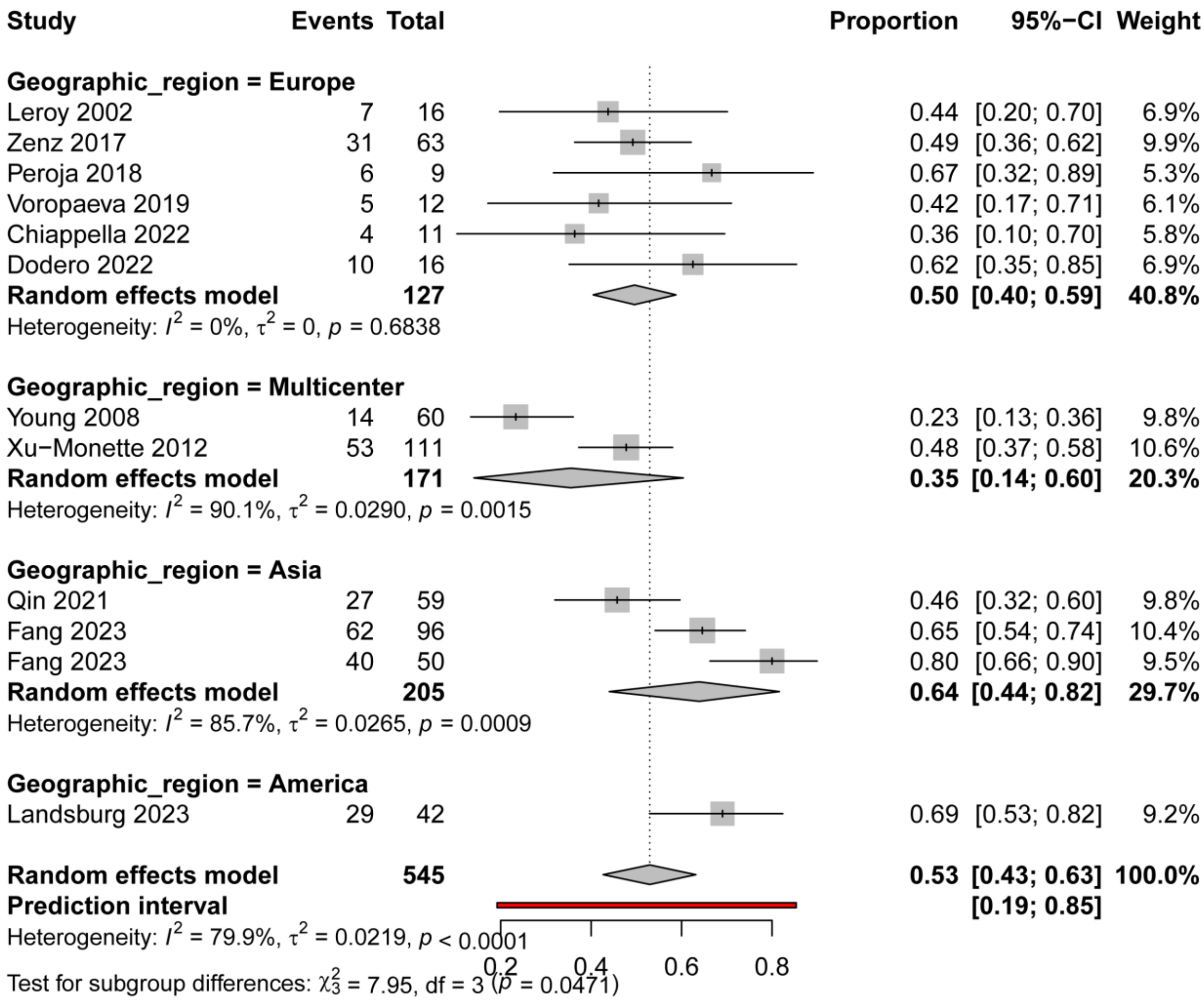

Fig. S21 Subgroup analysis of OS rate in nd DLBCL according to current remission strategies, stratified by region.

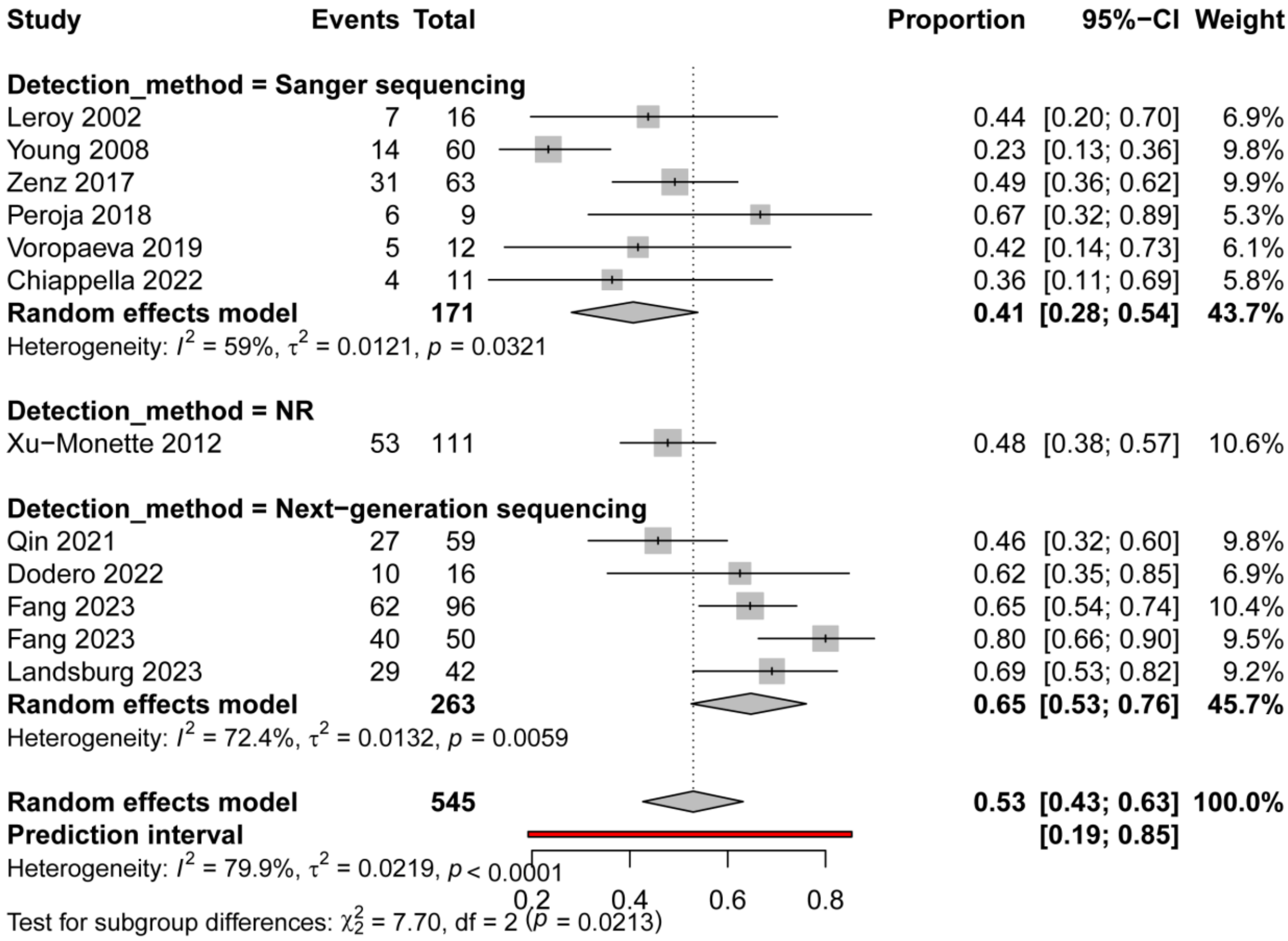

Fig. S22 Subgroup analysis of OS rate in nd DLBCL according to current remission strategies, stratified by detection method.

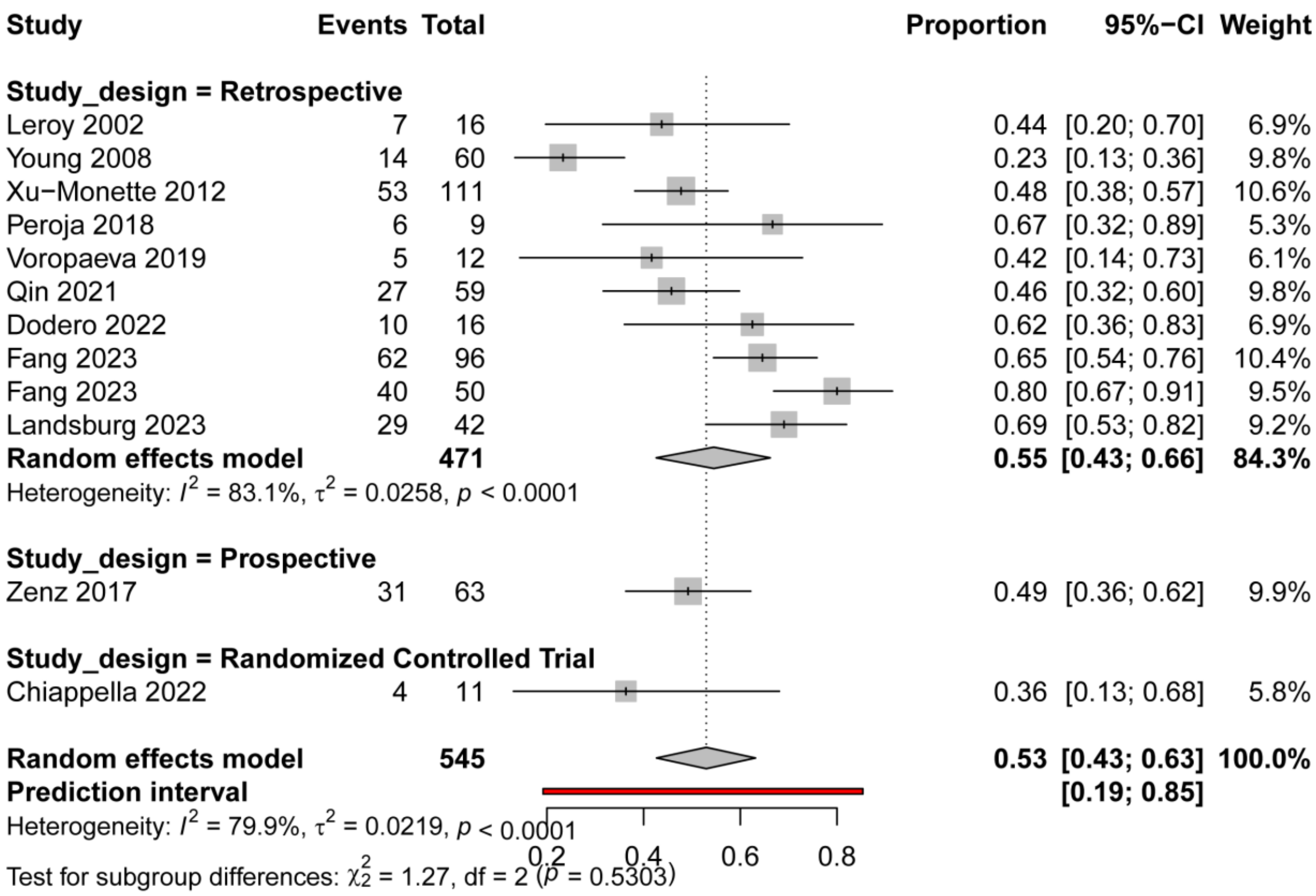

Fig. S23 Subgroup analysis of OS rate in nd DLBCL according to current remission strategies, stratified

by study design.

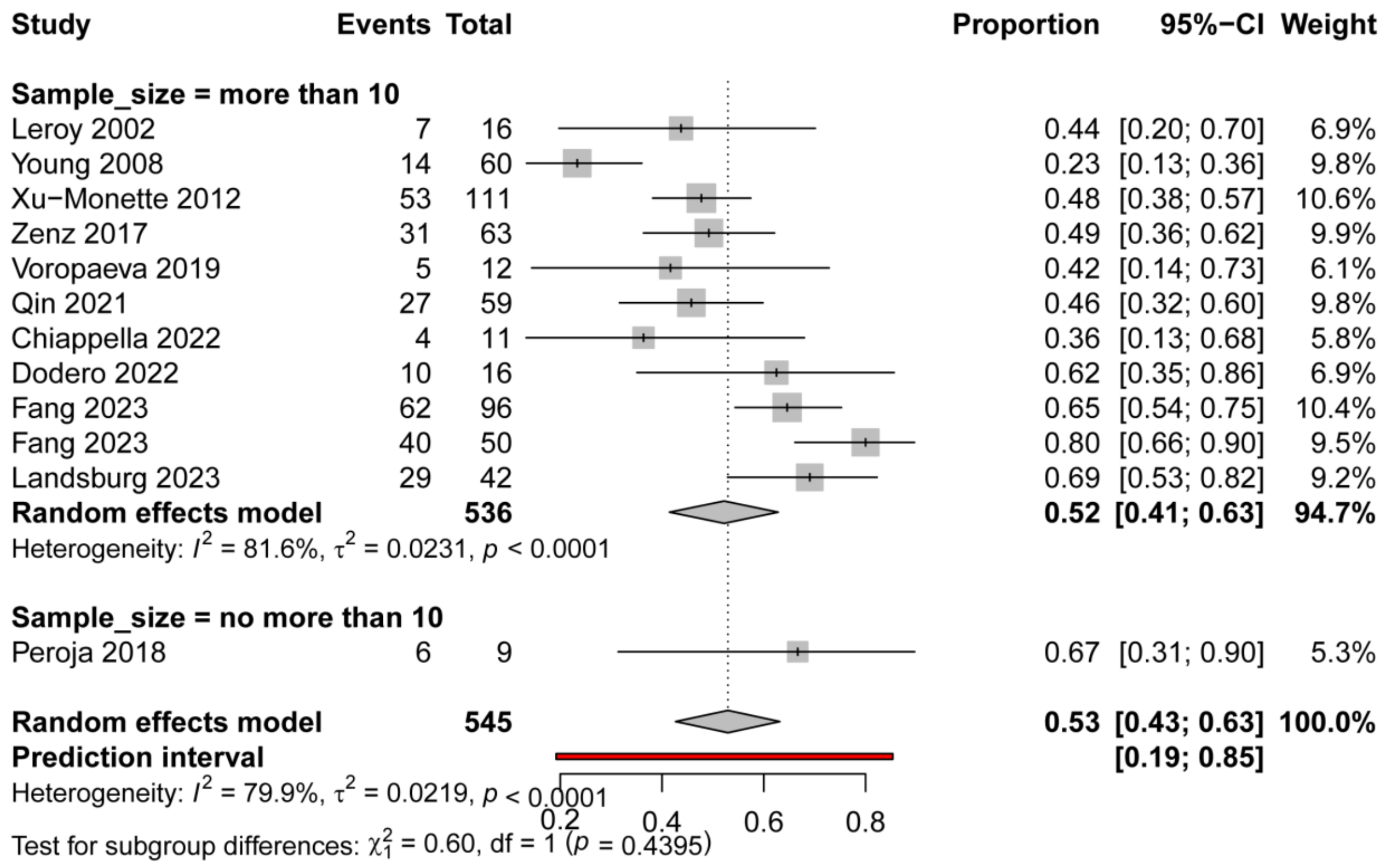

Fig. S24 Subgroup analysis of OS rate in nd DLBCL according to current remission strategies, stratified by sample size.

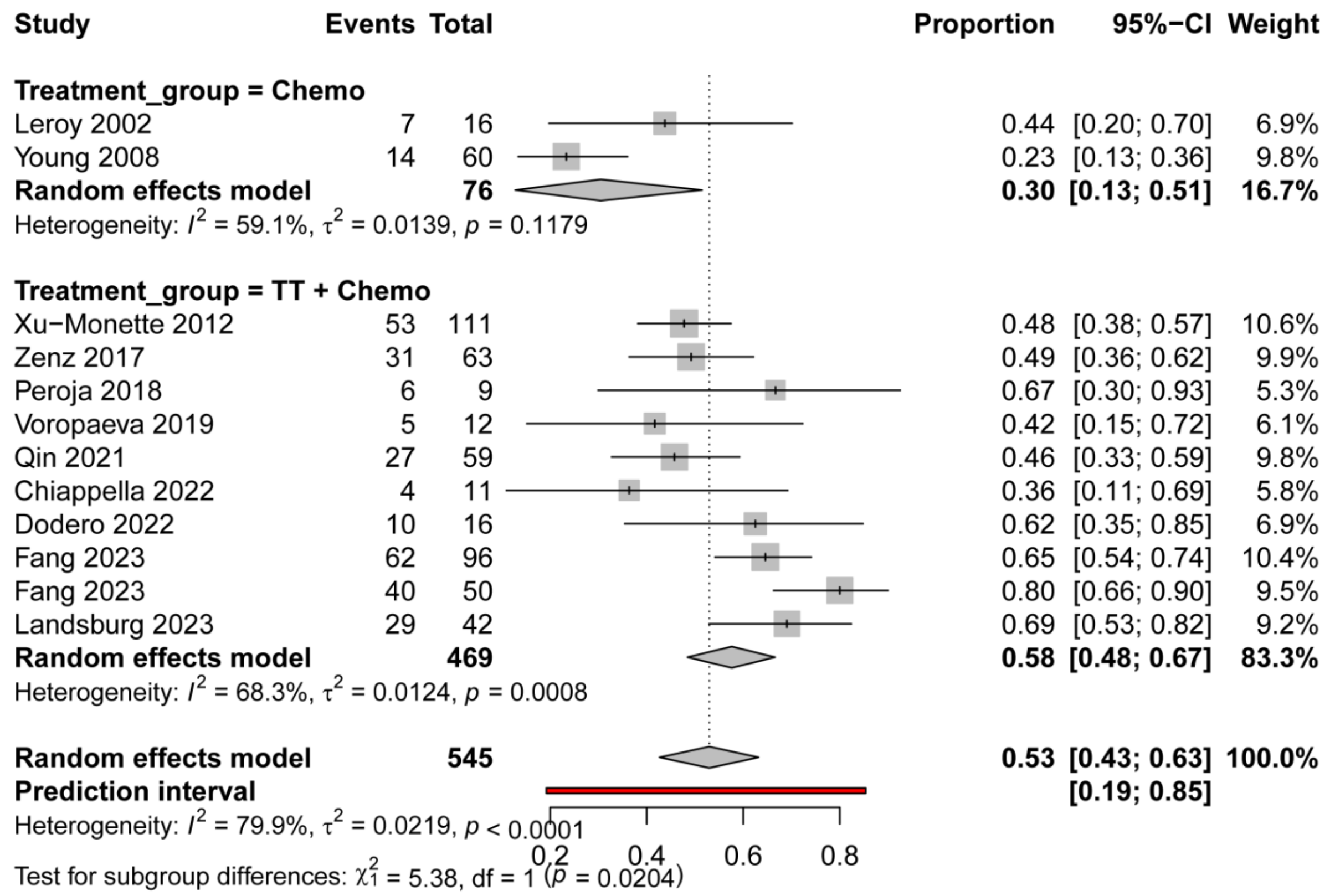

Fig. S25 Subgroup analysis of OS rate in nd DLBCL according to current remission strategies, stratified by therapeutic regimens.

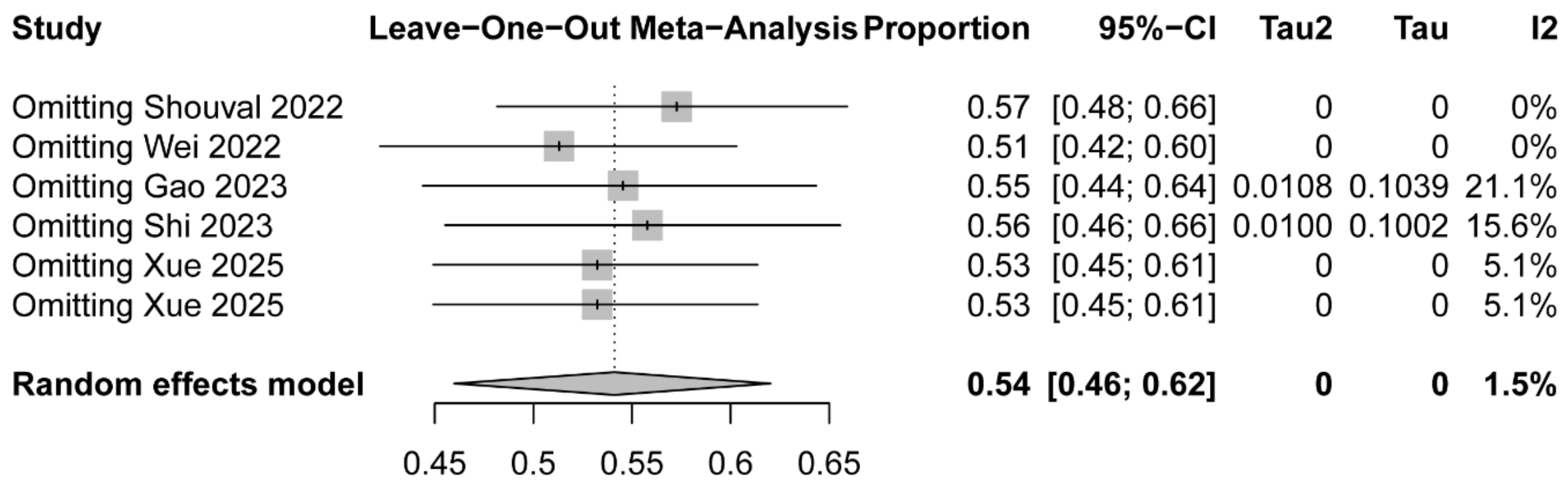

Fig. S26 Sensitivity analysis of OS rate in rr DLBCL by removing one study at a time.

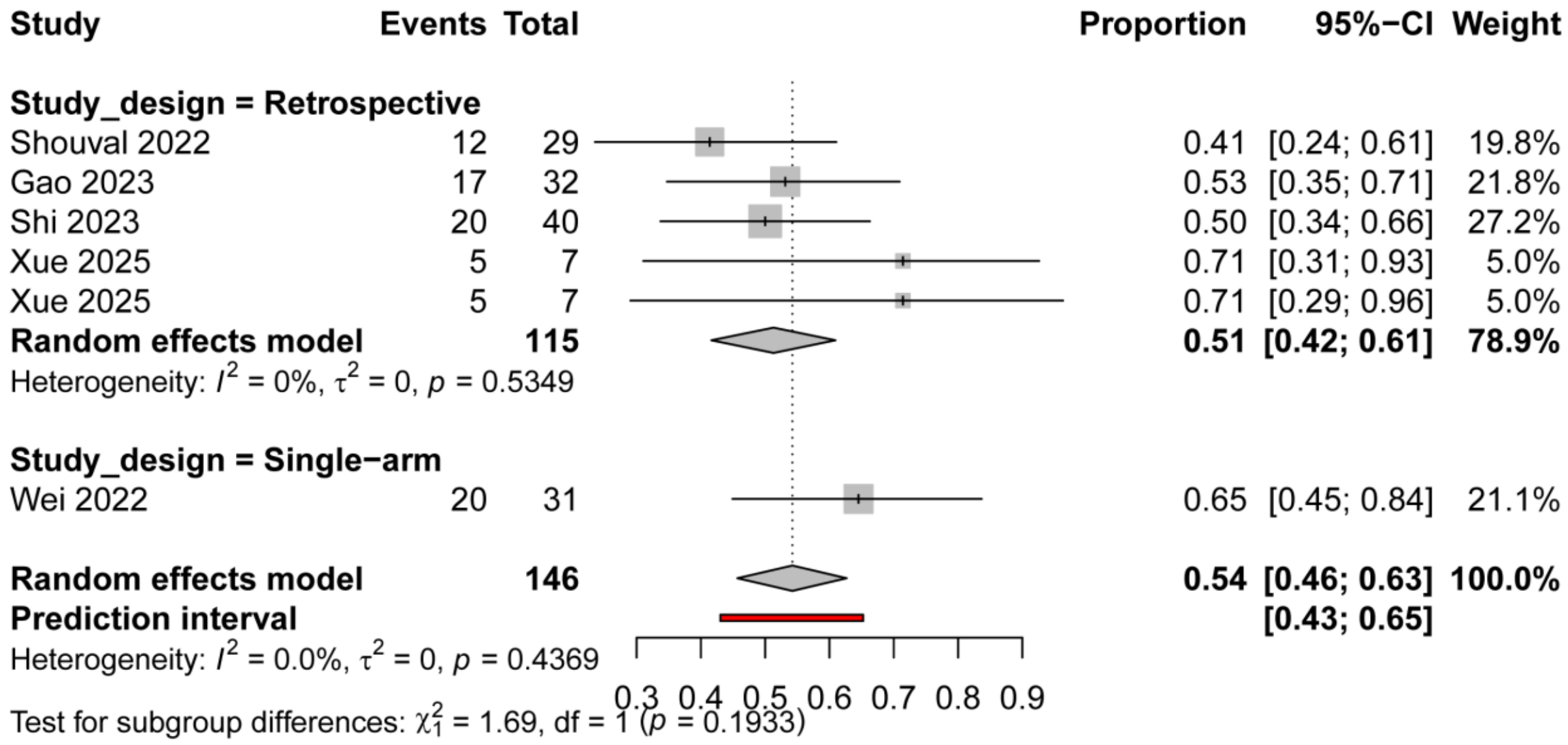

Fig. S27 Subgroup analysis of OS rate in rr DLBCL according to current remission strategies, stratified by study design.

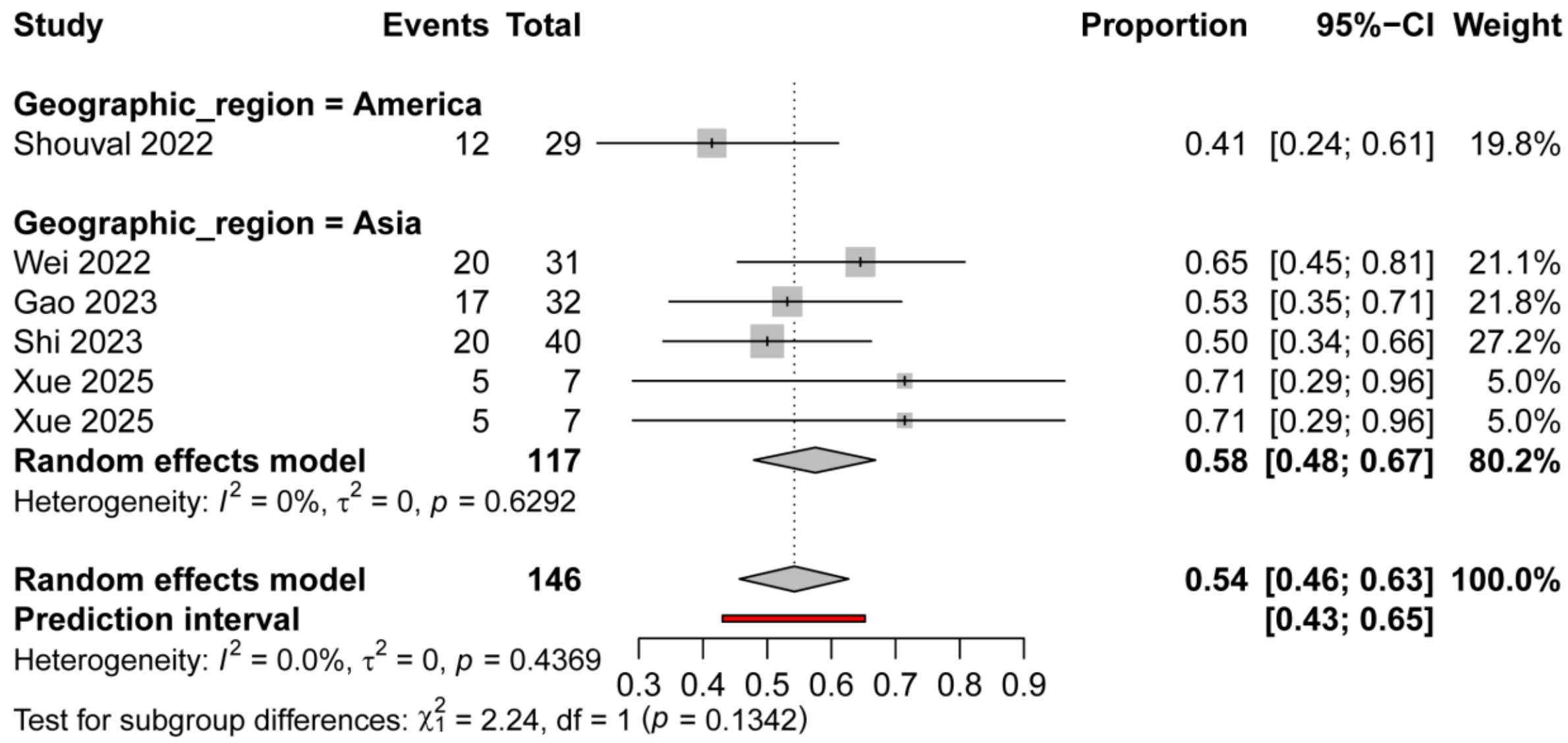

Fig. S28 Subgroup analysis of OS rate in rr DLBCL according to current remission strategies, stratified by region.

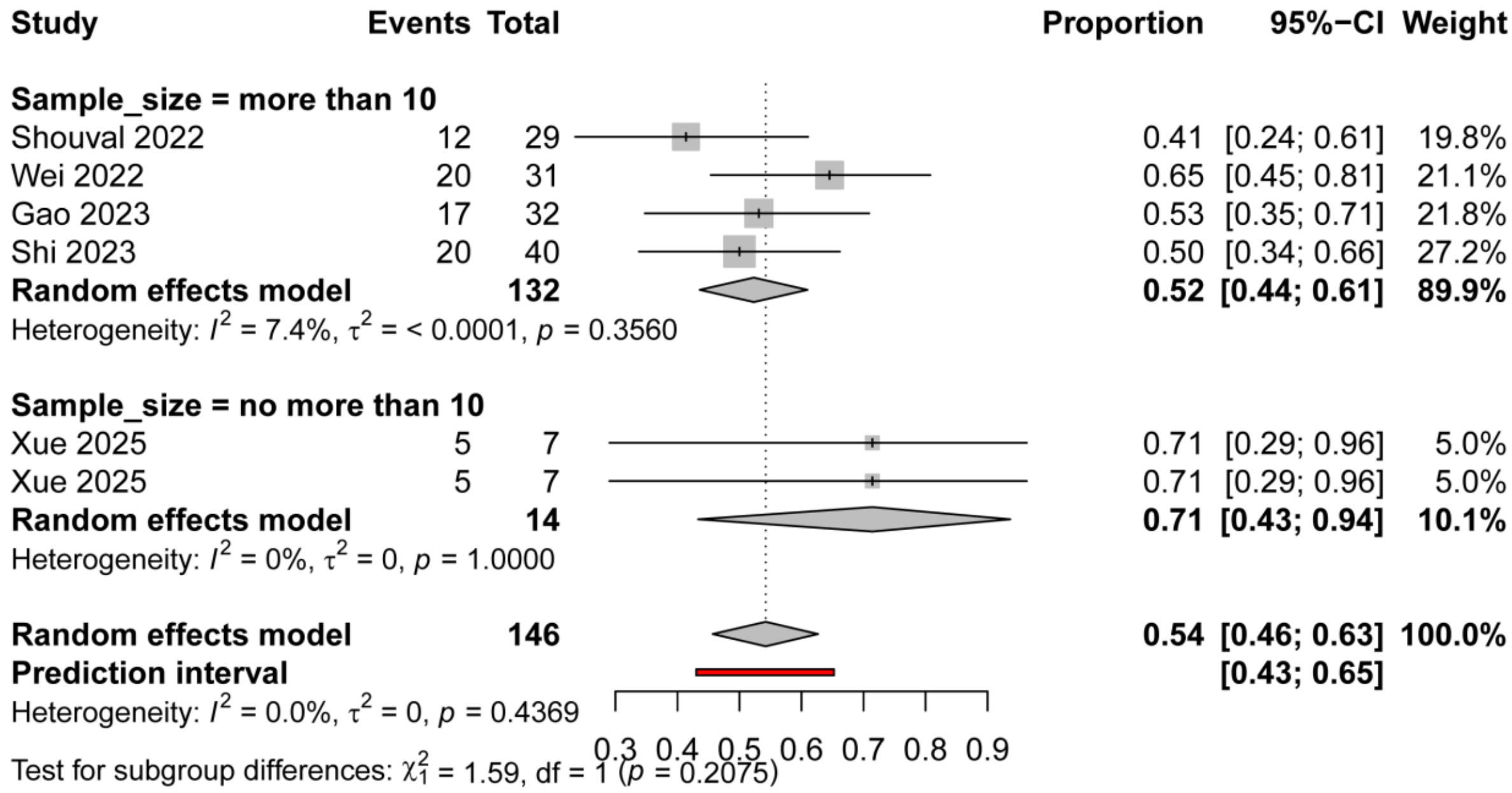

Fig. S29 Subgroup analysis of OS rate in rr DLBCL according to current remission strategies, stratified by sample size.

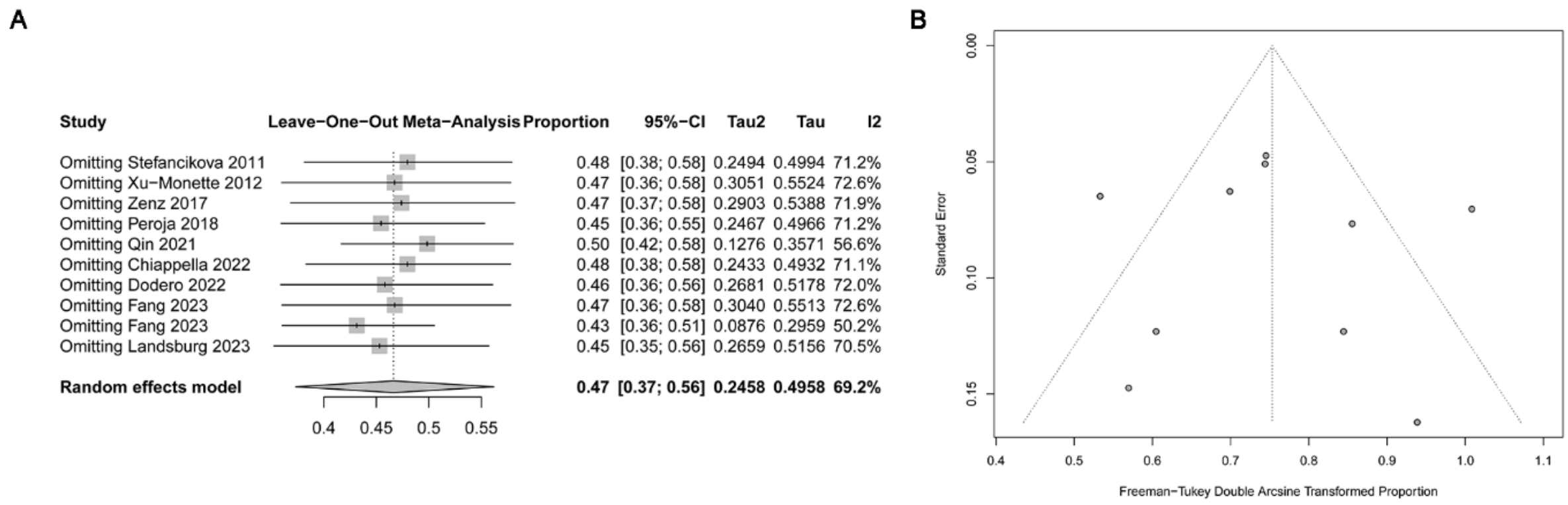

Fig. S30 Sensitivity analysis and publication bias assessment for the pooled progression-free survival rate in newly diagnosed TP53-mutated DLBCL. (A) Sensitivity analysis of the pooled PFS rate by removing one study at a time. (B) Funnel plot assessing publication bias for the pooled PFS rate. Each dot represents an individual study.

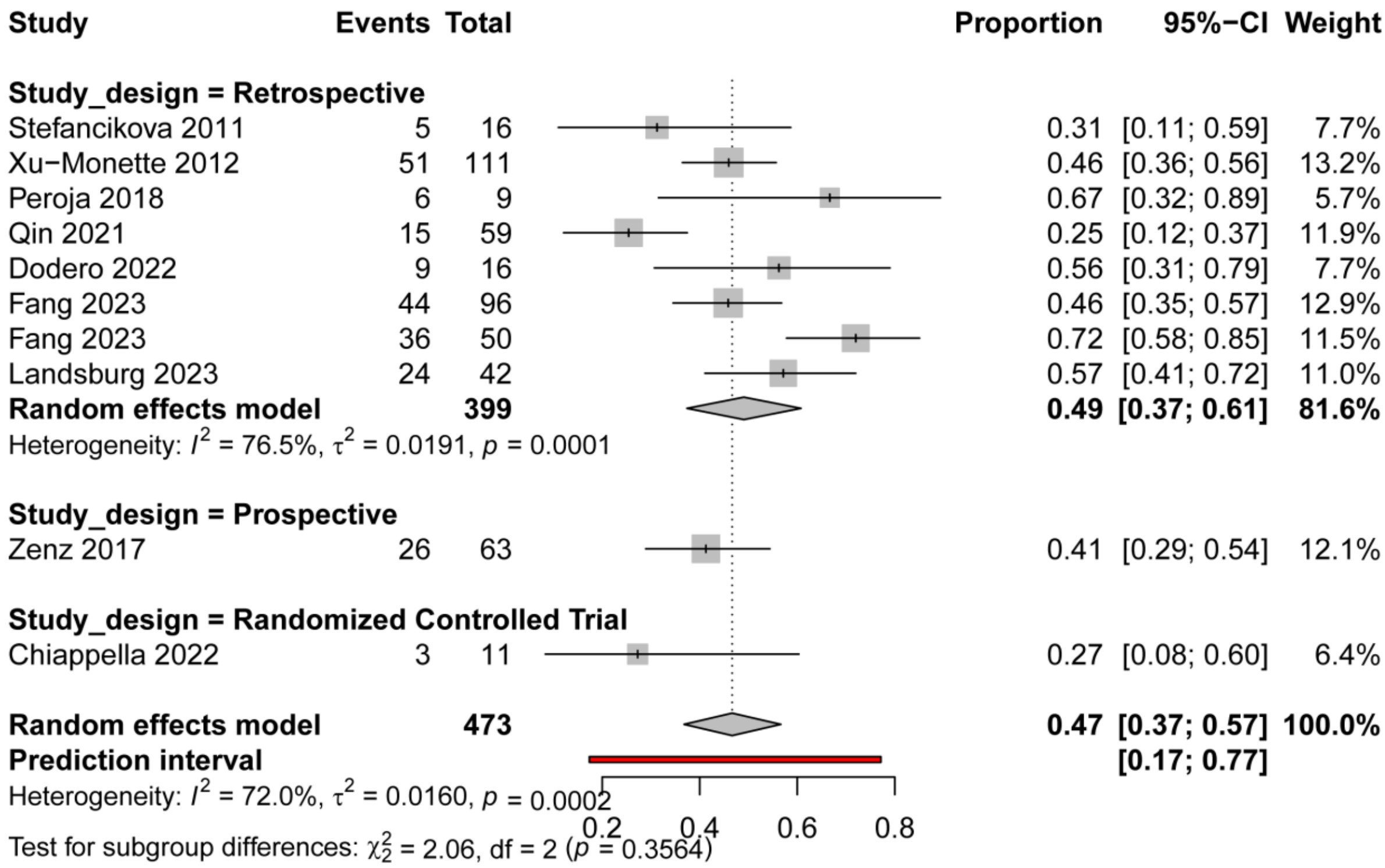

Fig. S31 Subgroup analysis of PFS rate in nd DLBCL according to current remission strategies, stratified by study design.

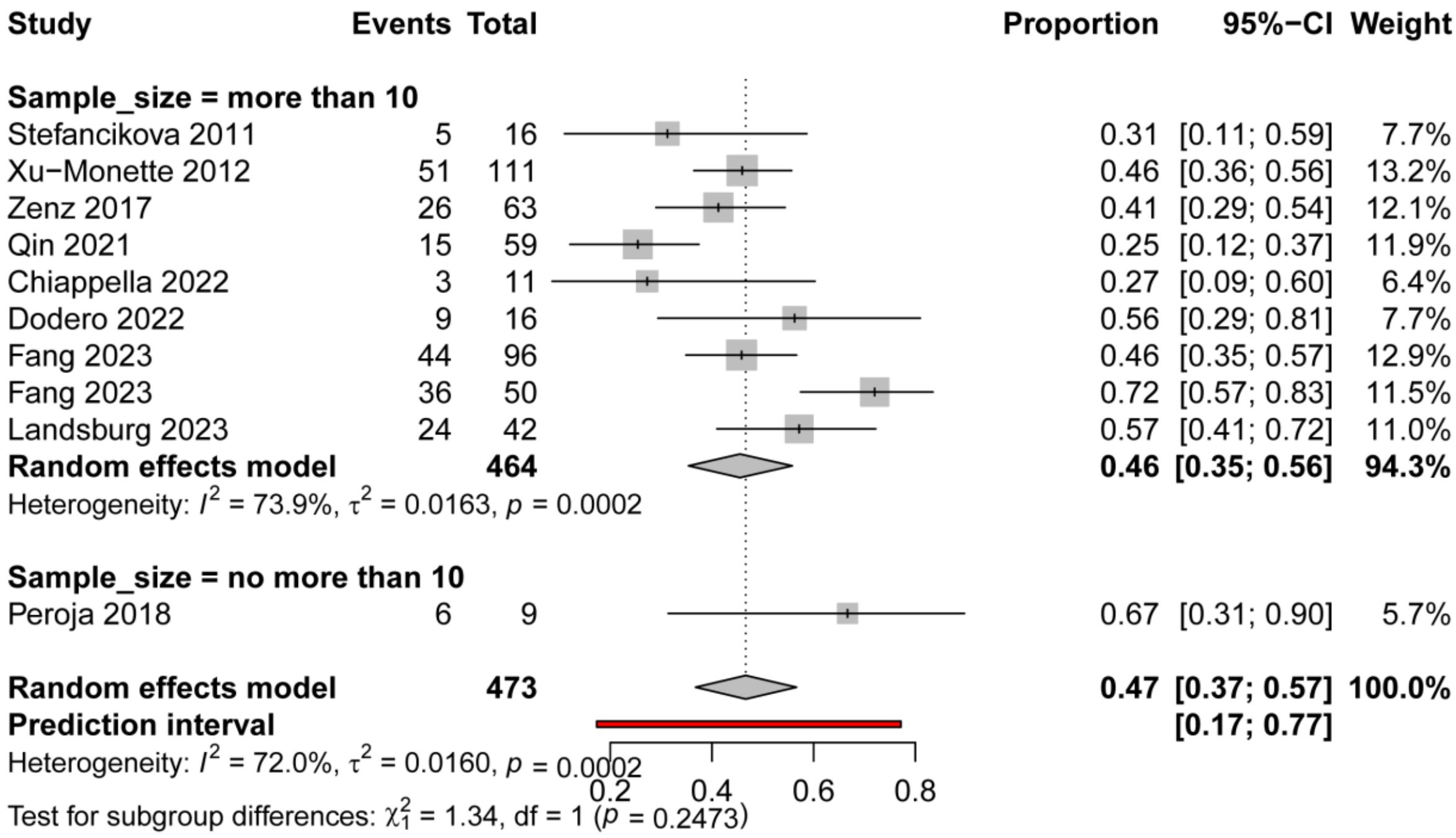

Fig. S32 Subgroup analysis of PFS rate in nd DLBCL according to current remission strategies, stratified by sample size.

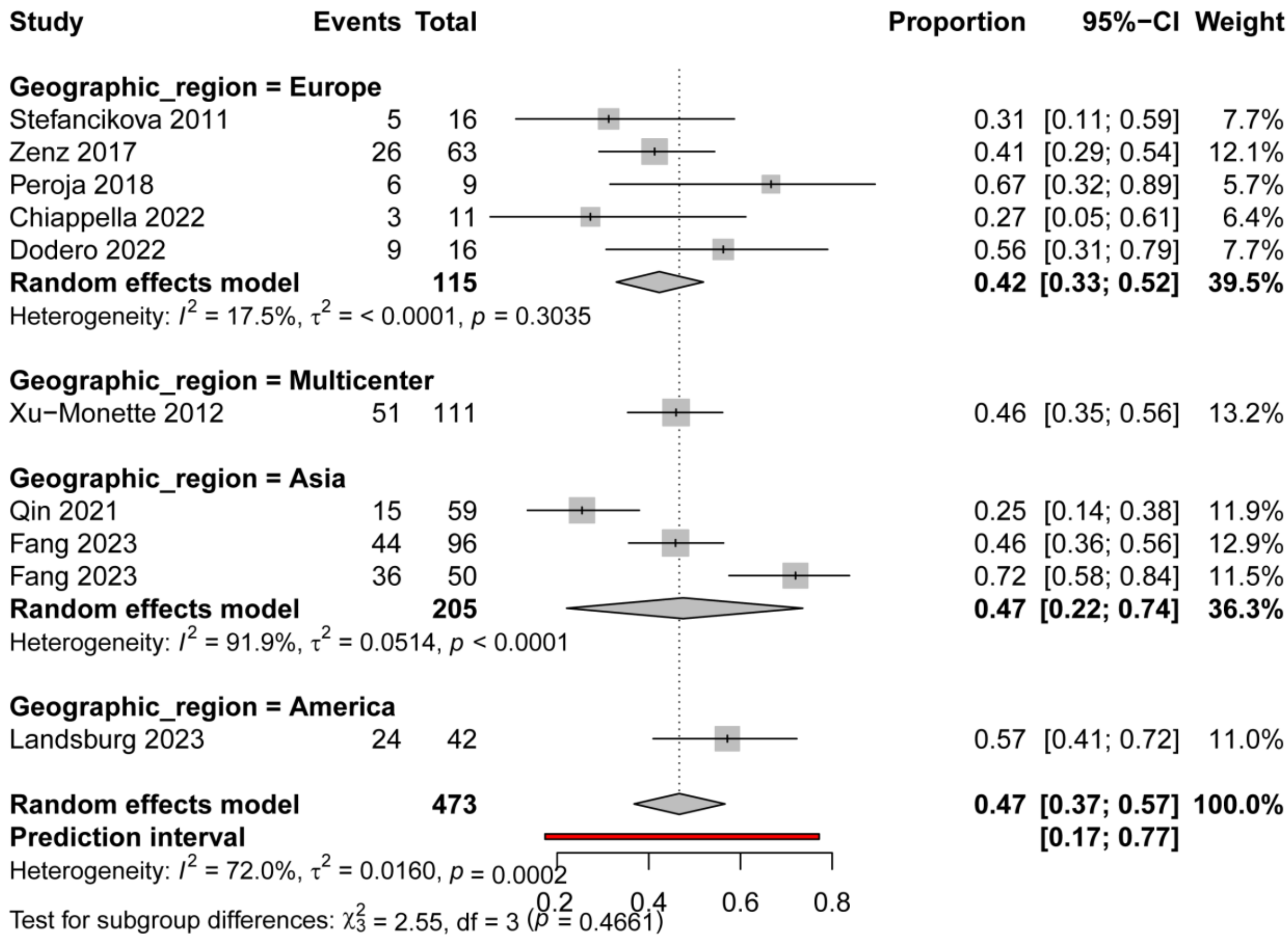

Fig. S33 Subgroup analysis of PFS rate in nd DLBCL according to current remission strategies, stratified by region.

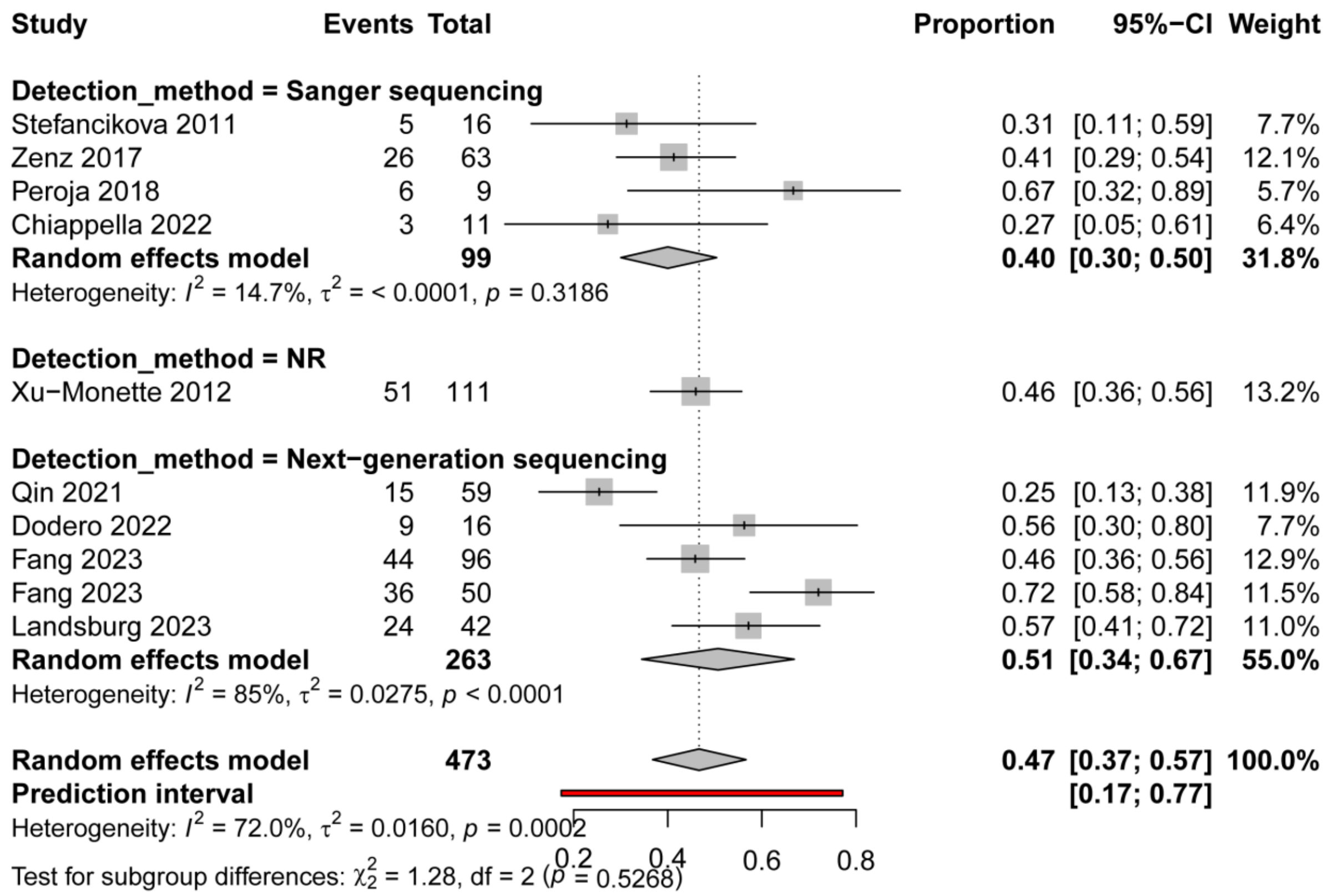

Fig. S34 Subgroup analysis of PFS rate in nd DLBCL according to current remission strategies, stratified by detection method.

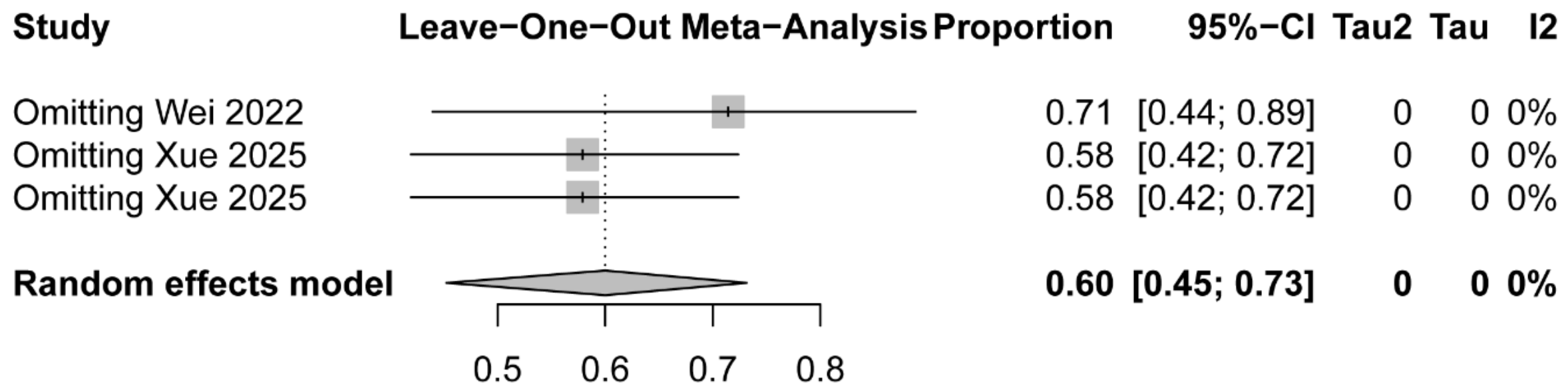

Fig. S35 Sensitivity analysis of PFS rate in rr DLBCL by removing one study at a time.

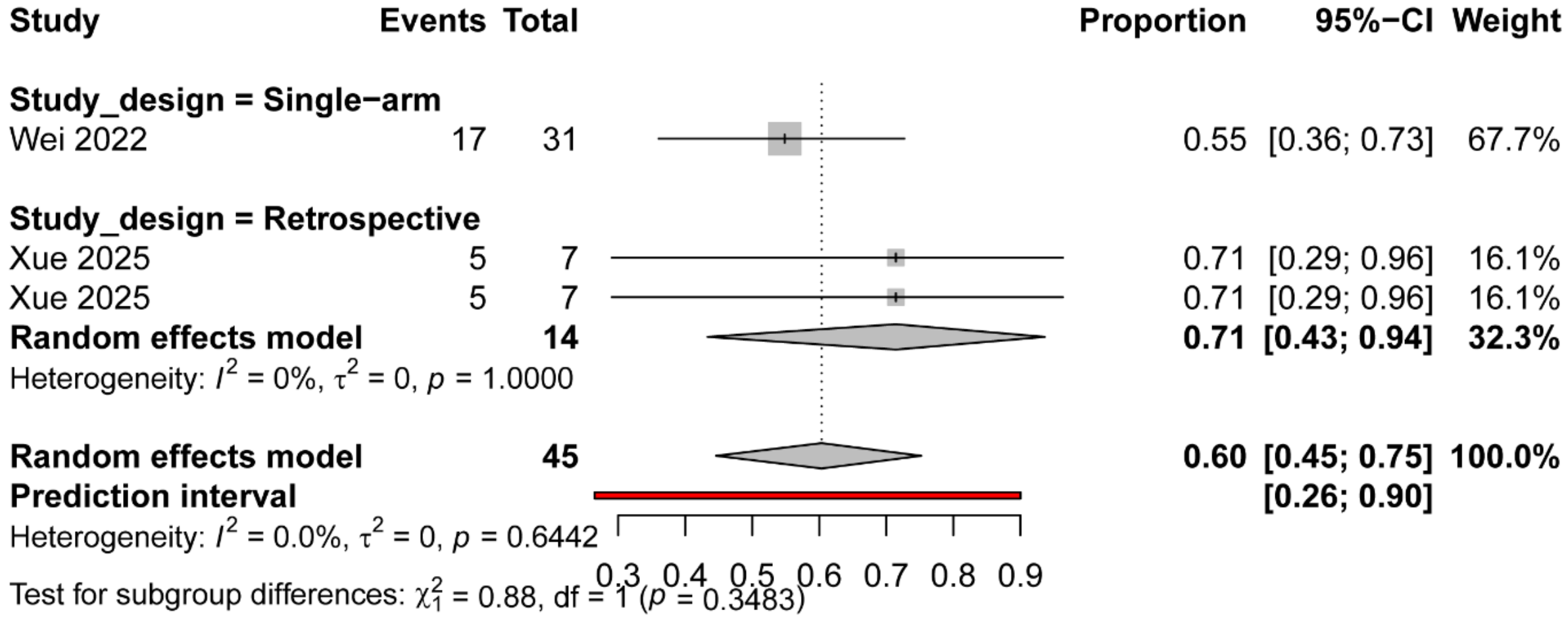

Fig. S36 Subgroup analysis of PFS rate in rr DLBCL according to current remission strategies, stratified by study design.

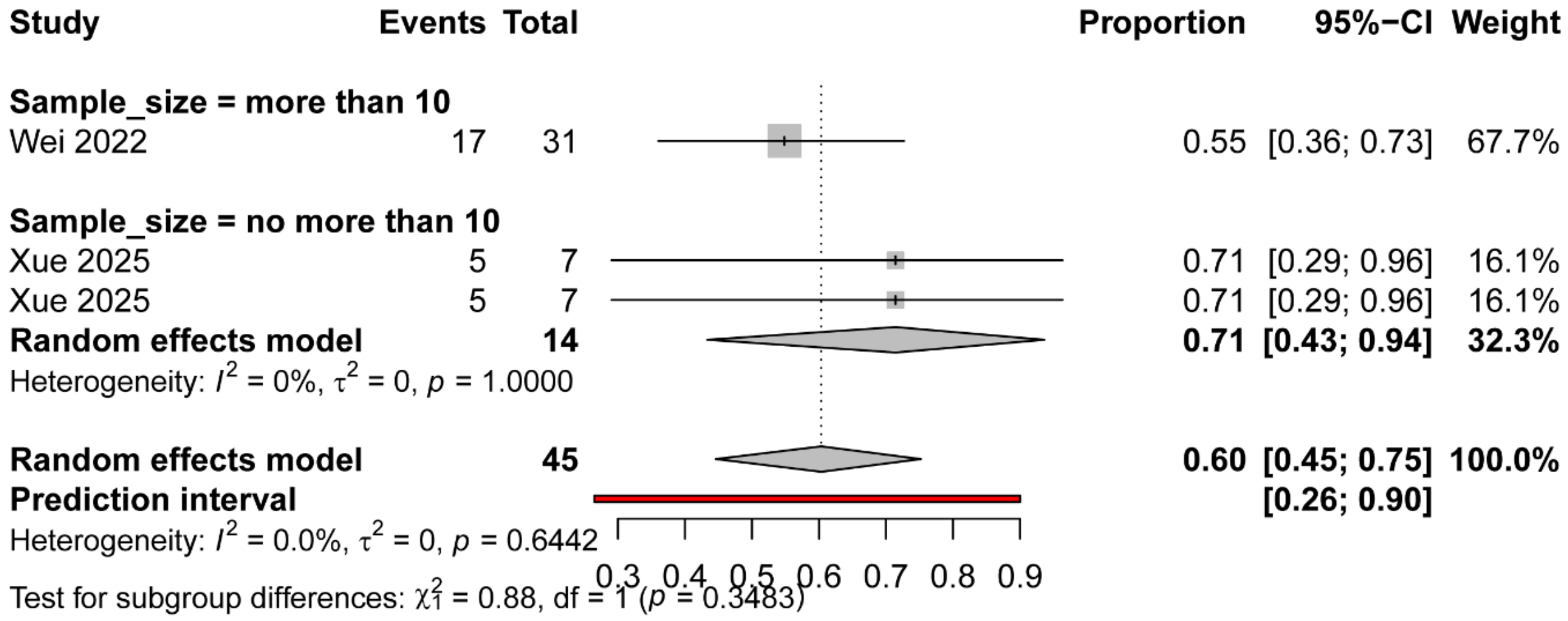

Fig. S37 Subgroup analysis of PFS rate in rr DLBCL according to current remission strategies, stratified by sample size.

Table S2. Quality assessment results of each included non-randomized study.

Article	1 Bias due to confounding	2 Bias in selection of participants into the study	3 Bias in classification of interventions	4 Bias due to deviations from intended interventions	5 Bias due to missing data	6 Bias in measurement of outcomes	7 Bias in selection of the reported result	overall
Leroy 2002	M	M	L	L	M	L	M	M
Young 2008	M	M	M	L	M	L	M	M
Stefancikova 2011	M	M	L	L	M	L	M	M
Xu-Monette 2012	M	M	L	L	L	L	M	M
Schiefer 2015	M	H	M	L	M	L	M	H
Zenz 2017	M	M	M	L	M	L	M	M
Peroja 2018	M	M	M	L	M	L	M	M
Voropaeva 2019	H	M	M	L	M	L	M	H
Qin 2020	M	M	L	M	M	L	M	M
Qin 2021	M	H	L	L	M	L	M	H
Zhang 2021	H	M	L	M	M	L	M	H
Dodero 2022	M	M	M	M	M	L	M	M
Fang 2023	M	M	L	M	M	L	M	M
Landsburg 2023	M	M	L	L	L	L	M	M
Jiang 2025	M	M	L	M	L	L	M	M
Jin 2025	M	M	L	L	M	L	M	M
Chen 2025	M	M	L	L	M	L	M	M
Porpaczy 2021	M	M	M	M	M	L	M	M
Wilson 2021	M	M	L	M	M	L	M	M
Yuan 2022	M	M	L	M	M	L	M	M

Shouval 2022	M	M	L	L	M	L	M	M
Wei 2022	M	M	L	M	M	L	M	M
Gao 2023	M	M	L	M	M	L	M	M
Qin 2023	M	M	L	M	M	L	M	M
Shi 2023	M	M	L	M	M	L	M	M
Xue 2024	M	M	L	M	L	L	M	M
Xu 2025	M	M	L	M	L	L	M	M
Xue 2025	M	M	L	M	L	L	M	M

Table footnote:

L, low risk of bias; M, moderate risk of bias; H, high risk of bias.

PRISMA 2020 Checklist

Data S1. PRISMA checklist

Section and Topic	Item #	Checklist item	Location where item is reported
**TITLE**			
Title	1	Identify the report as a systematic review.	Lines 3
**ABSTRACT**			
Abstract	2	See the PRISMA 2020 for Abstracts checklist.	Lines 23
**INTRODUCTION**			
Rationale	3	Describe the rationale for the review in the context of existing knowledge.	Lines 44-109
Objectives	4	Provide an explicit statement of the objective(s) or question(s) the review addresses.	Lines113-117
**METHODS**			
Eligibility criteria	5	Specify the inclusion and exclusion criteria for the review and how studies were grouped for the syntheses.	Lines130-147
Information sources	6	Specify all databases, registers, websites, organisations, reference lists and other sources searched or consulted to identify studies. Specify the date when each source was last searched or consulted.	Lines121-129
Search strategy	7	Present the full search strategies for all databases, registers and websites, including any filters and limits used.	Lines121-129
Selection process	8	Specify the methods used to decide whether a study met the inclusion criteria of the review, including how many reviewers screened each record and each report retrieved, whether they worked independently, and if applicable, details of automation tools used in the process.	Lines130-147
Data collection process	9	Specify the methods used to collect data from reports, including how many reviewers collected data from each report, whether they worked independently, any processes for obtaining or confirming data from study investigators, and if applicable, details of automation tools used in the process.	Lines148-165
Data items	10a	List and define all outcomes for which data were sought. Specify whether all results that were compatible with each outcome domain in each study were sought (e.g. for all measures, time points, analyses), and if not, the methods used to decide which results to collect.	Lines148-165
	10b	List and define all other variables for which data were sought (e.g. participant and intervention characteristics, funding sources). Describe any assumptions made about any missing or unclear information.	Lines148-165
Study risk of bias assessment	11	Specify the methods used to assess risk of bias in the included studies, including details of the tool(s) used, how many reviewers assessed each study and whether they worked independently, and if applicable, details of automation tools used in the process.	Lines153-165
Effect measures	12	Specify for each outcome the effect measure(s) (e.g. risk ratio, mean difference) used in the synthesis or presentation of results.	Lines171-190
Synthesis methods	13a	Describe the processes used to decide which studies were eligible for each synthesis (e.g. tabulating the study intervention characteristics and comparing against the planned groups for each synthesis (item #5)).	Lines204-214
	13b	Describe any methods required to prepare the data for presentation or synthesis, such as handling of missing summary statistics, or data conversions.	Lines204-214
	13c	Describe any methods used to tabulate or visually display results of individual studies and syntheses.	Lines204-214
	13d	Describe any methods used to synthesize results and provide a rationale for the choice(s). If meta-analysis was performed, describe the model(s), method(s) to identify the presence and extent of statistical heterogeneity, and software package(s) used.	Lines204-214

Section and Topic	Item #	Checklist item	Location where item is reported
	13e	Describe any methods used to explore possible causes of heterogeneity among study results (e.g. subgroup analysis, meta-regression).	Lines204-214
	13f	Describe any sensitivity analyses conducted to assess robustness of the synthesized results.	Lines191-200
Reporting bias assessment	14	Describe any methods used to assess risk of bias due to missing results in a synthesis (arising from reporting biases).	Lines216-224
Certainty assessment	15	Describe any methods used to assess certainty (or confidence) in the body of evidence for an outcome.	Lines171-178
**RESULTS**			
Study selection	16a	Describe the results of the search and selection process, from the number of records identified in the search to the number of studies included in the review, ideally using a flow diagram.	Lines 204
	16b	Cite studies that might appear to meet the inclusion criteria, but which were excluded, and explain why they were excluded.	Lines204-214
Study characteristics	17	Cite each included study and present its characteristics.	Lines213-214
Risk of bias in studies	18	Present assessments of risk of bias for each included study.	Lines216-224
Results of individual studies	19	For all outcomes, present, for each study: (a) summary statistics for each group (where appropriate) and (b) an effect estimate and its precision (e.g. confidence/credible interval), ideally using structured tables or plots.	Lines226-352
Results of syntheses	20a	For each synthesis, briefly summarise the characteristics and risk of bias among contributing studies.	Lines226-352
	20b	Present results of all statistical syntheses conducted. If meta-analysis was done, present for each the summary estimate and its precision (e.g. confidence/credible interval) and measures of statistical heterogeneity. If comparing groups, describe the direction of the effect.	Lines226-352
	20c	Present results of all investigations of possible causes of heterogeneity among study results.	Lines226-352
	20d	Present results of all sensitivity analyses conducted to assess the robustness of the synthesized results.	Lines226-352
Reporting biases	21	Present assessments of risk of bias due to missing results (arising from reporting biases) for each synthesis assessed.	Lines226-352
Certainty of evidence	22	Present assessments of certainty (or confidence) in the body of evidence for each outcome assessed.	Lines226-352
**DISCUSSION**			
Discussion	23a	Provide a general interpretation of the results in the context of other evidence.	Lines 354
	23b	Discuss any limitations of the evidence included in the review.	Lines422-442
	23c	Discuss any limitations of the review processes used.	Lines422-442
	23d	Discuss implications of the results for practice, policy, and future research.	Lines445-

PRISMA 2020 Checklist

Section and Topic	Item #	Checklist item	Location where item is reported
			464
**OTHER INFORMATION**			
Registration and protocol	24a	Provide registration information for the review, including register name and registration number, or state that the review was not registered.	Lines 124
	24b	Indicate where the review protocol can be accessed, or state that a protocol was not prepared.	Lines 124
	24c	Describe and explain any amendments to information provided at registration or in the protocol.	N/A
Support	25	Describe sources of financial or non-financial support for the review, and the role of the funders or sponsors in the review.	Lines 497
Competing interests	26	Declare any competing interests of review authors.	Lines 496
Availability of data, code and other materials	27	Report which of the following are publicly available and where they can be found: template data collection forms; data extracted from included studies; data used for all analyses; analytic code; any other materials used in the review.	Lines 492

From: Page MJ, McKenzie JE, Bossuyt PM, Boutron I, Hoffmann TC, Mulrow CD, et al. The PRISMA 2020 statement: an updated guideline for reporting systematic reviews. BMJ 2021;372:n71. doi: 10.1136/bmj.n71
